# Naphthalimide-phenothiazine dyads: effect of conformational flexibility and matching of the energy of the charge-transfer state and the localized triplet excited state on the thermally activated delayed fluorescence

**DOI:** 10.3762/bjoc.18.149

**Published:** 2022-10-11

**Authors:** Kaiyue Ye, Liyuan Cao, Davita M E van Raamsdonk, Zhijia Wang, Jianzhang Zhao, Daniel Escudero, Denis Jacquemin

**Affiliations:** 1 State Key Laboratory of Fine Chemicals, Frontiers Science Center for Smart Materials, School of Chemical Engineering, Dalian University of Technology, Dalian 116024, P. R. China; 2 Department of Chemistry, KU Leuven, B-3001 Leuven, Belgium; 3 State Key Laboratory of Chemistry and Utilization of Carbon Based Energy Resources, College of Chemistry, Xinjiang University, Urumqi 830017, P. R. China; 4 Nantes Université, CNRS, CEISAM UMR-6230, Nantes F-44000, France

**Keywords:** charge-transfer, electron donor, intersystem crossing, TADF, triplet state

## Abstract

In order to investigate the joint influence of the conformation flexibility and the matching of the energies of the charge-transfer (CT) and the localized triplet excited (^3^LE) states on the thermally activated delayed fluorescence (TADF) in electron donor–acceptor molecules, a series of compact electron donor–acceptor dyads and a triad were prepared, with naphthalimide (NI) as electron acceptor and phenothiazine (PTZ) as electron donor. The NI and PTZ moieties are either directly connected at the 3-position of NI and the *N*-position of the PTZ moiety via a C–N single bond, or they are linked through a phenyl group. The tuning of the energy order of the CT and LE states is achieved by oxidation of the PTZ unit into the corresponding sulfoxide, whereas conformation restriction is imposed by introducing *ortho*-methyl substituents on the phenyl linker, so that the coupling magnitude between the CT and the ^3^LE states can be controlled. The singlet oxygen quantum yield (Φ_Δ_) of **NI-PTZ** is moderate in *n*-hexane (HEX, Φ_Δ_ = 19%). TADF was observed for the dyads, the biexponential luminescence lifetime are 16.0 ns (99.9%)/14.4 μs (0.1%) for the dyad and 7.2 ns (99.6%)/2.0 μs (0.4%) for the triad. Triplet state was observed in the nanosecond transient absorption spectra with lifetimes in the 4–48 μs range. Computational investigations show that the orthogonal electron donor–acceptor molecular structure is beneficial for TADF. These calculations indicate small energetic difference between the ^3^LE and ^3^CT states, which are helpful for interpreting the ns-TA spectra and the origins of TADF in **NI-PTZ**, which is ultimately due to the small energetic difference between the ^3^LE and ^3^CT states. Conversely, **NI-PTZ-O**, which has a higher CT state and bears a much more stabilized ^3^LE state, does not show TADF.

## Introduction

Thermally activated delayed fluorescence (TADF) has attracted much attention in recent years, not only for its application in organic light emitting diodes (OLED) [1–3] but also as a mean for studying of charge-transfer (CT) and intersystem-crossing (ISC) phenomena [4–5]. Compounds showing TADF are usually presenting an orthogonal electron donor–acceptor molecular structure, i.e., the π-planes of the electron donor and acceptor adopt an orthogonal geometry [6–7]. Such an architecture is beneficial to spatially split HOMO and LUMO orbitals, thus reducing the electron-exchange integral (*J*) for the two electrons in the frontier molecular orbitals which reduced the energy gap (2*J*) between these two states. lt is widely considered that this small energy gap (a few dozens of meV) is beneficial for both ISC and the reverse ISC (rISC) in TADF [1–3,8–13].

However, it is noted that in some electron donor–acceptor dyads, TADF is not observed even when the CT state is accessible [14–15]. This is typically because the direct ISC between ^1^CT and ^3^CT is forbidden and non-efficient, and this hyperfine interaction-driven ISC is slow. Recently, it was proposed that an intermediate localized triplet state (^3^LE) is essential to enhance the ISC and rISC, through the so-called spin-vibronic coupling effect [8,16–19]. However, the effect of the molecular geometry on the ISC and rlSC is complicated, and additional investigations are required to verify the above postulate and to unravel the TADF mechanism.

Recently, we and others found that the orthogonal donor–acceptor dyad derived from 1,8-naphthalimide (NI) and phenothiazine (PTZ) shows TADF in the red spectral range [20–22]. Our purpose of designing that dyad was to study the spin–orbit charge-transfer ISC (SOCT-ISC), i.e., to determine, if the ISC is enhanced by the charge recombination (CR) in the orthogonal dyad. Indeed, CR is accompanied by orbital angular momentum change, which off-sets the electron spin angular momentum change, allowing the angular momentum to be conserved and, consequently, ISC to be enhanced [23–31]. We underline that the orthogonal geometry of this dyad reduces the ^1^CT/^3^CT states energy gap while simultaneously enhancing the ISC for the ^1^CT→^3^LE process. Therefore, we propose that the TADF is actually a special case of SOCT-ISC, when the three ^1^CT/^3^CT/^3^LE states have similar energies. Nevertheless, in most orthogonal dyads showing SOCT-ISC, the ^3^LE state has a much lower energy than the CT states, especially in triplet photosensitizers, for which a final ^3^LE state is desired [32–33]. As explained above, an orthogonal geometry is beneficial to achieve SOCT-ISC. However, for TADF, it was proposed that the rotational freedom is beneficial for the rISC, and that too rigid molecular structures may favor phosphorescence and therefore limit rISC and TADF [34–35]. Studies with time-resolved electron paramagnetic resonance (TREPR) spectra and theoretical methods also support that conformation fluctuations are beneficial to TADF [16]. This is in stark contrast with the SOCT-ISC mechanism.

To further explore these contradictory requirements for SOCT-ISC and TADF, we designed herein a series of **NI-PTZ** dyads, and the synthesis route is mentioned in the following section of molecular design and structural confirmation. These dyads are different from the previously reported dyads by the substitution position, and the number of PTZ moieties attached on the NI unit, as well as the redox potential of the PTZ moiety. **NI-PTZ** has a linkage at the 3-position of the NI moiety, for the recently reported analogous dyad, however, the substitution is at the 4-position [20]. For the current **NI-PTZ** dyad, the torsion between the NI and PTZ has a larger freedom, due to the reduced steric hindrance originating from the *peri*-H atoms on the two chromophores. We also tuned the redox potentials by oxidation of the electron donor PTZ (**NI-PTZ-O**). Thus, the energy of CT states and the matching with their ^3^LE counterpart can be altered. We underline that the approach of oxidation of the PTZ unit, which has minimal impacts on the geometry and the ^3^LE state energy in the dyad, was rarely explored [8]. We also modified the energy of the CT states by increasing the distance between the electron donor and acceptor by using an intervening phenyl linker between the NI and the PTZ moieties (**NI-Ph-PTZ** and **NI-PhMe_2_-PTZ**) [36]. The electronic coupling between the NI and PTZ units differ these two dyads. Finally, in **NI-PhMe_2_-PTZ** with methyl substituents, the phenyl linker adopts an orthogonal geometry with respect to the NI moiety, inducing a weaker coupling than that in **NI-Ph-PTZ**. The photophysical properties of the dyads were studied with steady-state and time-resolved spectroscopic methods, as well as theoretical calculations.

## Results and Discussion

### Molecular design and structure confirmation

**Scheme 1:**
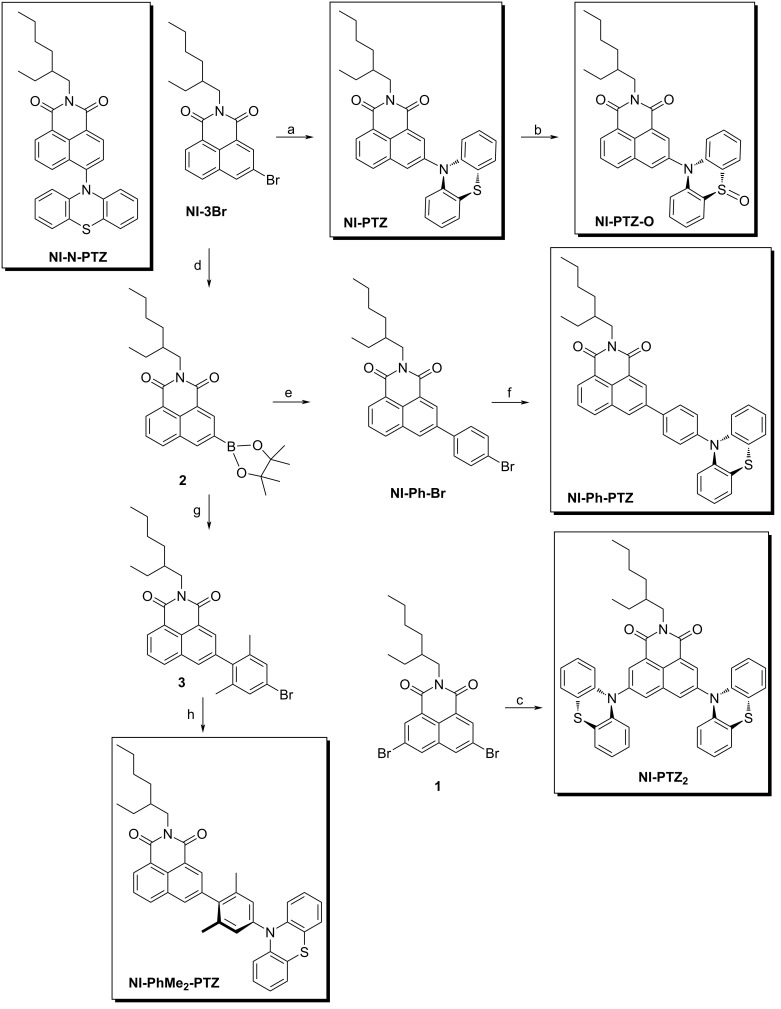
Synthesis of the compounds^a^. ^a^Key: (a) phenothiazine, sodium *tert*-butoxide, dried toluene (TOL), tri-*tert*-butylphosphine tetrafluoroborate, Pd(OAc)_2_, 120 °C, 8 h, 93.1%; (b) H_2_O_2_ (30%), CH_3_COOH, 40 °C, 1 h, yield: 87.2%; (c) similar to step (a), yield: 80.0%; (d) bis(pinacolato)diboron, KOAc, Pd(dppf)Cl_2_, toluene, N_2_, 110 °C, 16 h, yield: 11.9%.; (e) 1-bromo-4-iodobenzene, Pd(PPh_3_)_4_, K_2_CO_3_, TOL, EtOH, H_2_O, N_2_, reflux, 8 h, yield: 92.9%; (f) similar to step (a), yield: 62.4%; (g) 5-bromo-2-iodo-1,3-dimethylbenzene, Pd(PPh_3_)_4_, K_2_CO_3_, TOL, EtOH, H_2_O, N_2_, 110 °C, 9 h, yield: 60.6%; (h) similar to step (a), yield: 28.3%.

In order to study the effect of conformational flexibility on TADF, **NI-PTZ** was designed (Scheme 1). As dicussed above, various approaches (different connection, linkers, oxidation of the PTZ, and addition of methyl groups) have been used to tune the relative energies of the key states and the geometry. Scheme 1 summarizes the synthetic routes used to obtain the various compounds and shows the molecular structures. The synthesis of the dyads is based on the ordinary derivatization of the NI and PTZ chromophores [20]. The molecular structures were confirmed by ^1^H NMR, ^13^C NMR, and HRMS methods (Experimental section).

### UV–vis absorption and fluorescence emission spectra

**Figure 1:**
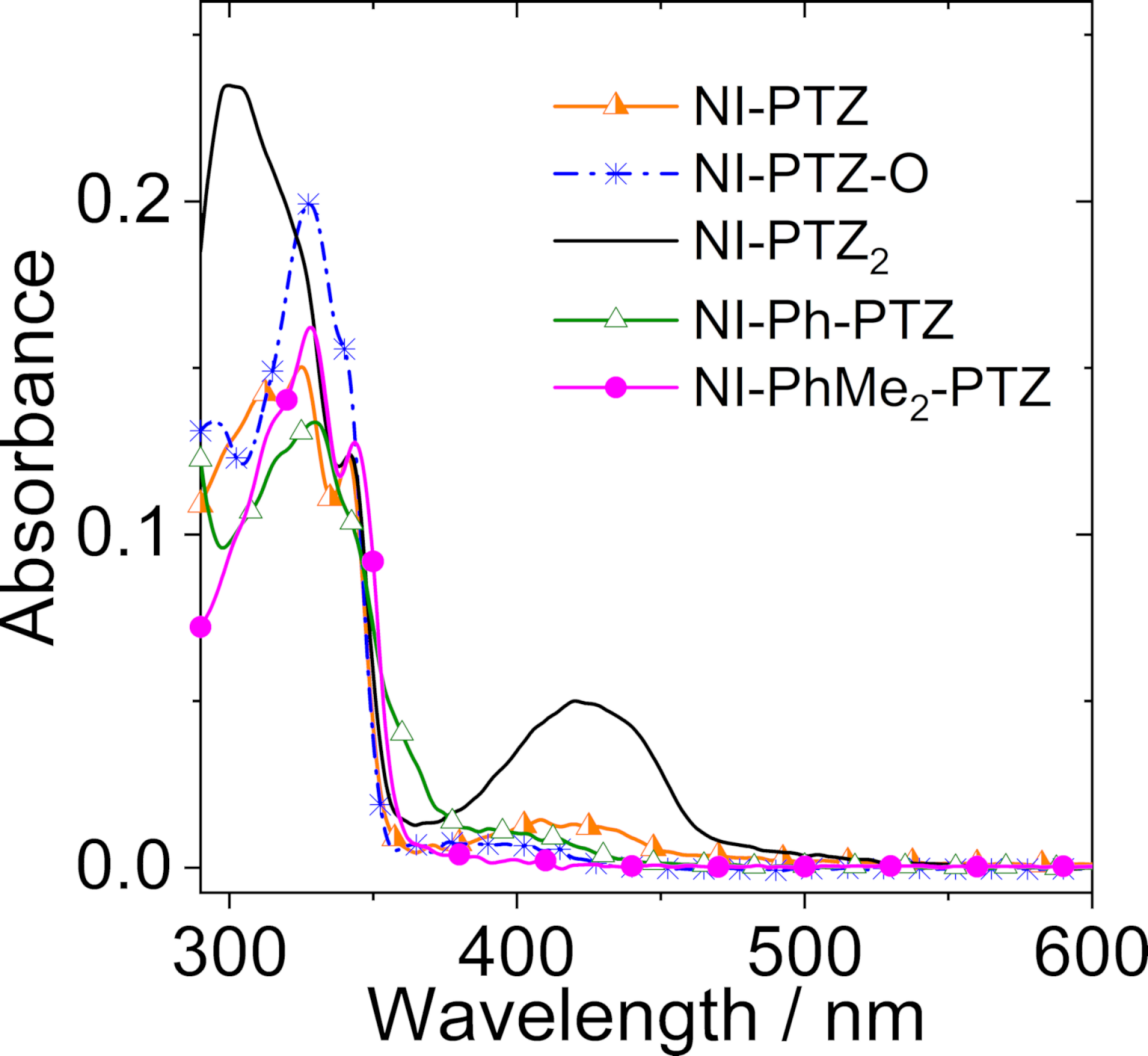
UV–vis absorption spectra of **NI-PTZ**, **NI-PTZ-O**, **NI-PTZ_2_**, **NI-Ph-PTZ**, and **NI-PhMe_2_-PTZ** in HEX. *c* = 1.0 × 10^−5^ M at 20 °C.

The UV–vis absorption spectra of the compounds were studied (Figure 1 and Figure S25 in Supporting Information File 1). **NI-PTZ** shows structured absorption bands in the 300–350 nm range, which are attributed to the NI moiety [20]. Moreover, there is a broad, structureless absorption band centered at 412 nm (ε = 1.30 × 10^3^ M^−1^ cm^−1^), which is assigned to a CT absorption band, i.e., to the S_0_→^1^CT transition. This is an indication of the strong electronic coupling between the electron donor (PTZ) and acceptor (NI). Indeed, in the absence of such coupling, the S_0_→^1^CT transition would be forbidden, and no CT absorption band would be observed [14,37–41].

Interestingly, the CT absorption is similar to the previously reported **NI-N-PTZ** dyad with a linkage at the 4-position of the NI moiety [20], for which the CT absorption band is centered at 411 nm. Interesting in the **NI-PTZ_2_** triad, the absorption band at 300–350 nm is different from that of **NI-PTZ**, and the CT absorption band centered at 423 nm is much more intense than that of **NI-PTZ**, indicating that the electronic effect of the substituents does not have a simple additive effect on the photophysical properties [42]. The CT absorption band of **NI-PTZ-O** is much weaker, confirming that the CT absorption band strongly depends on the electron-donating ability of the donor. In both **NI-Ph-PTZ** and **NI-PhMe_2_-PTZ**, the CT band is negligible, due to the large separation between the NI and PTZ moieties. Note that in **NI-Ph-PTZ**, the electronic coupling between the NI and the phenyl linker is non-negligible, which results in a CT absorption in which the phenyl moiety acts as the donor. This analysis is supported by the UV–vis absorption spectrum of the 3-phenyl NI [43]. A careful examination of the UV–vis absorption spectra indicates that the PTZ moiety in **NI-Ph-PTZ** induces a slight redshift of the CT absorption band (centered at 405 nm) as compared to that of 3-phenyl NI [43].

These results show that our methods for tuning the electronic coupling between the donor and acceptor groups by alternation of the redox potentials of the donor (or acceptor), variation of the distance between the donor and acceptor, and conformational restriction, are all successful [44–46].

**Figure 2:**
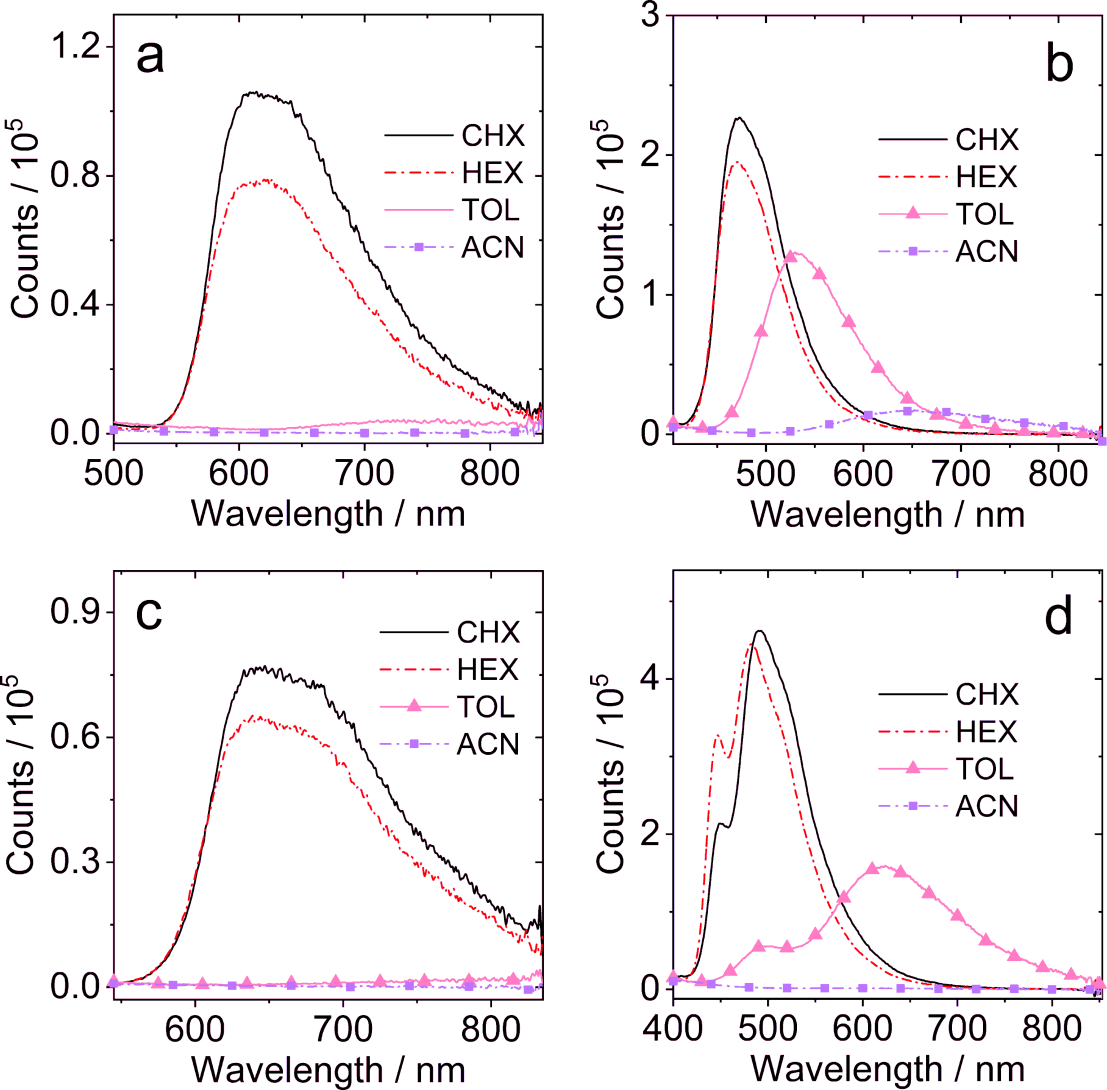
Fluorescence spectra of the compounds (a) **NI-PTZ**; (b) **NI-PTZ-O**; (c) **NI-PTZ_2_**, and (d) **NI-Ph-PTZ** in different solvents. The solvents used are: CHX, HEX, TOL and acetonitrile (ACN). Optically matched solutions were used, *A* = 0.100, λ_ex_ = 330 nm, 20 °C.

The fluorescence of the dyads was studied (Figure 2 and Figure S26 in Supporting Information File 1). As compared to that in cyclohexane (CHX) and HEX, the fluorescence of **NI-PTZ** is strongly quenched in TOL and solvents with higher polarity. This trend is similar to the one previously reported for the **NI-N-PTZ** analog [20]. We note that the CT emission band of **NI-PTZ** is slightly red-shifted as compared to that of the previously reported dyad. Upon oxidation of the PTZ moiety, i.e., for **NI-PTZ-O**, the fluorescence quenching in polar solvents is less significant than that of **NI-PTZ** (Figure 2b), and the CT emission band is blue-shifted as compared to that of **NI-N-PTZ** [20], a likely consequence of the reduced electron-donating ability of the PTZ moiety.

For **NI-PTZ_2_**, a solvent polarity-dependent fluorescence band was observed (Figure 2c), which is similar to that of **NI-PTZ**. For **NI-Ph-PTZ**, a structured fluorescence band was observed in the 400–600 nm range (Figure 2d), which is assigned to LE emission. In toluene, however, a broad emission band centered at 624 nm was observed, which we attribute to the CT emission (with the phenyl moiety as donor group). The emission maximum (624 nm) is blue-shifted as compared to that of **NI-PTZ** (731 nm in toluene), indicating that the CT state energy of **NI-Ph-PTZ** is higher than that of **NI-PTZ** [47]. Similar results were observed for **NI-PhMe_2_-PTZ** (Supporting Information File 1, Figure S26).

**Figure 3:**
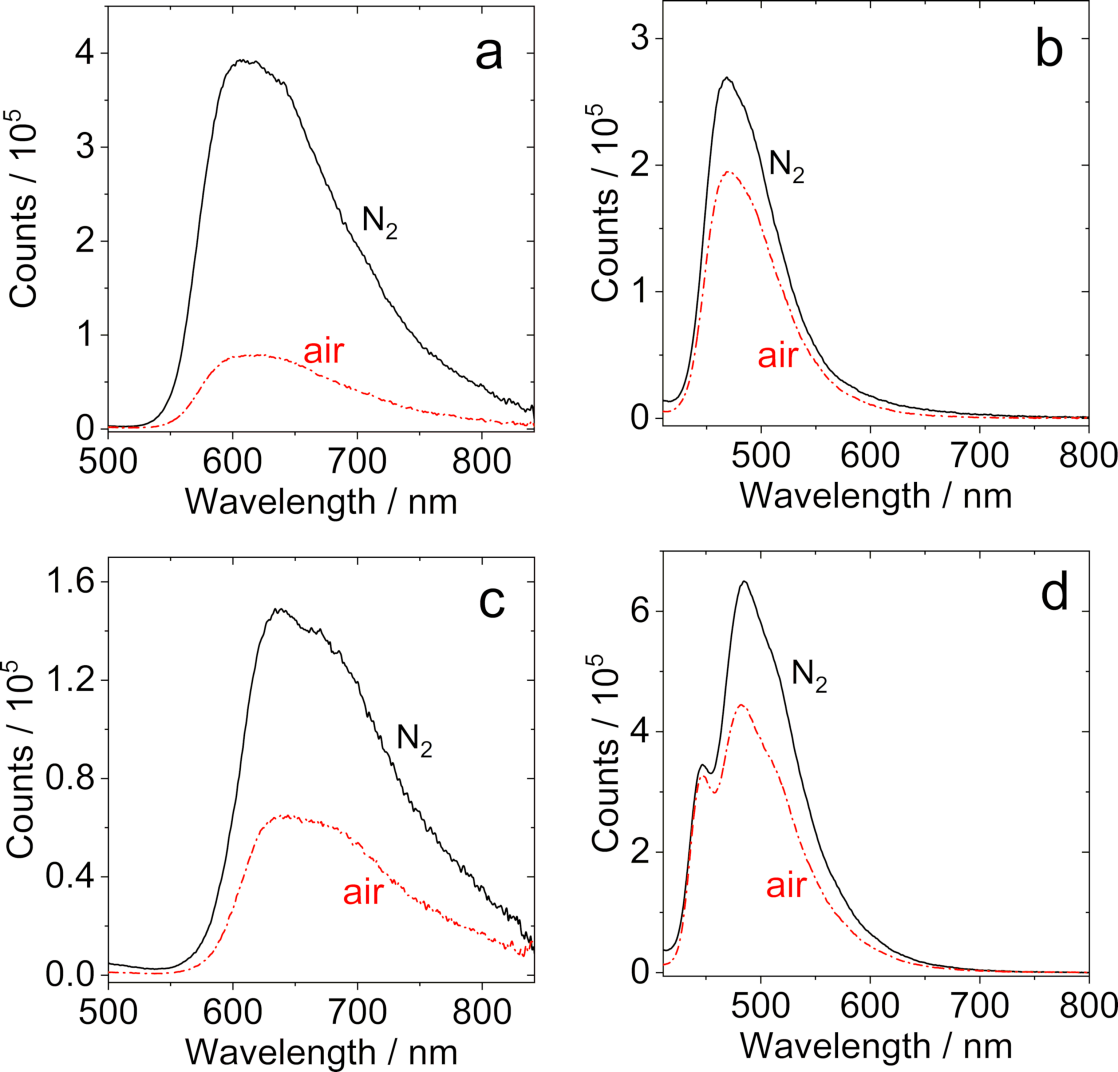
Fluorescence spectra of (a) **NI-PTZ**, (b) **NI-PTZ-O**, (c) **NI-PTZ_2_**, and (d) **NI-Ph-PTZ** in HEX under different atmospheres (N_2_, air). Optically matched solutions were used, *A* = 0.100, λ_ex_ = 330 nm, *c* = 1.0 × 10^−5^ M, 20 °C.

As a preliminary study to assess the existence of TADF for the dyads and the triad, the fluorescence spectra of the compounds in N_2_-saturated and air-saturated solution were recorded (Figure 3 and Figure S27 in Supporting Information File 1). For both **NI-PTZ** and **NI-PTZ_2_**, the fluorescence intensity was quenched significantly in aerated solution as compared to that in deaerated solution (Figure 3a and 3c and Figure S27a and S27c in Supporting Information File 1). For **NI-PTZ-O** and **NI-Ph-PTZ** (Figure 3b and 3d), however, the fluorescence intensity is less dependent on the atmosphere.

However, one should be careful with the interpretation of such data, as the quenching of the fluorescence in aerated solution does not necessarily imply TADF, since fluorescence species with long fluorescence lifetime can be also quenched by O_2_ (a paramagnetic species). This is in particular relevant for the present compounds, since the fluorescence of **NI-PTZ** and **NI-PTZ_2_** originate from CT states, whereas the emissions of **NI-PTZ-O** and **NI-Ph-PTZ** come from an emissive ^1^LE state (due to the oxidation of the PTZ unit, or the phenyl linker, the CT state energy increases, and the ^1^LE state becomes the lowest-lying state). Longer lifetimes are typically found for CT emission than LE emission, because of the forbidden feature of the ^1^CT→S_0_ transition.

**Figure 4:**
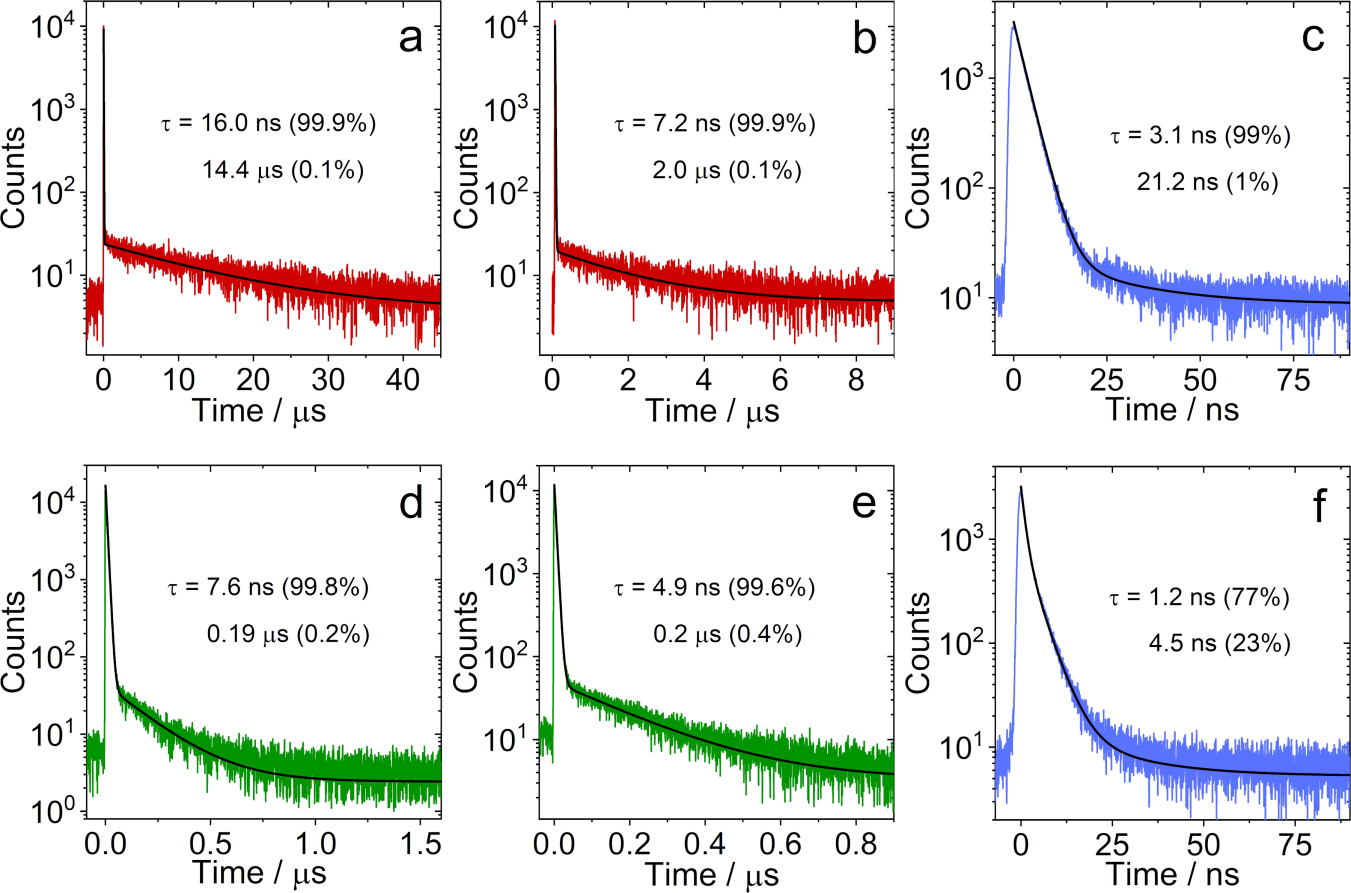
Fluorescence lifetime of **NI-PTZ** under (a) N_2_ atmosphere and (d) air atmosphere (λ_em_ = 610 nm, *c* = 5.0 × 10^−5^ M). Fluorescence lifetime of **NI-PTZ_2_** under (b) N_2_ atmosphere and (e) air atmosphere (λ_em_ = 610 nm, *c* = 5.0 × 10^−5^ M). Fluorescence lifetime of (c) **NI-PTZ-O** (λ_em_ = 472 nm, *c* = 1.0 × 10^−5^ M) and (f) **NI-Ph-PTZ** (λ_em_ = 482 nm, *c* = 1.0 × 10^−5^ M). Excited with a picoseconds pulsed laser (λ_ex_ = 340 nm), in HEX, 20 °C.

Therefore, the fluorescence decay trace of the compounds was recorded (Figure 4). The fluorescence decay trace of **NI-PTZ** shows a distinct biexponential signature, the lifetime is 16.0 ns (99.9%)/14.4 μs (0.1%) in deaerated *n-*hexane (Figure 4a). In aerated solution, the luminescence lifetime is reduced to 7.6 ns (99.8%)/0.19 μs (0.2%) (Figure 4d). These are footprints for TADF. Similar features were reported for an analogous **NI-PTZ** dyad [20]. **NI-PTZ_2_** displays similar characteristic TADF lifetimes (Figure 4b and 4e). Clearly, besides the conformational flexibility, other factors do play a role in the photophysical properties of the dyad, i.e., the magnitudes of CT/^3^LE energy gap, and related spin–vibronic couplings. Increasing the CT state energy either through oxidation of the PTZ moiety (for **NI-PTZ-O**) or by using a longer linker (**NI-Ph-PTZ** and **NI-PhMe_2_-PTZ**), leads to a normal fluorescence decay. Specifically, the luminescence lifetimes of **NI-PTZ-O** and **NI-Ph-PTZ** are 3.1 ns (99%)/22.1 ns (1%) and 1.2 ns (77%)/4.5 ns (23%), respectively (Figure 4c and 4f). The fluorescence lifetimes of **NI-PhMe_2_-PTZ** in CHX, HEX, and TOL were determined to be 1.6 ns (56%)/12.9 ns (44%), 1.2 ns (51%)/7.2 ns (49%), and 2.7 ns (22%)/18.7 ns (78%), respectively (Figure S28 in Supporting Information File 1).

**Figure 5:**
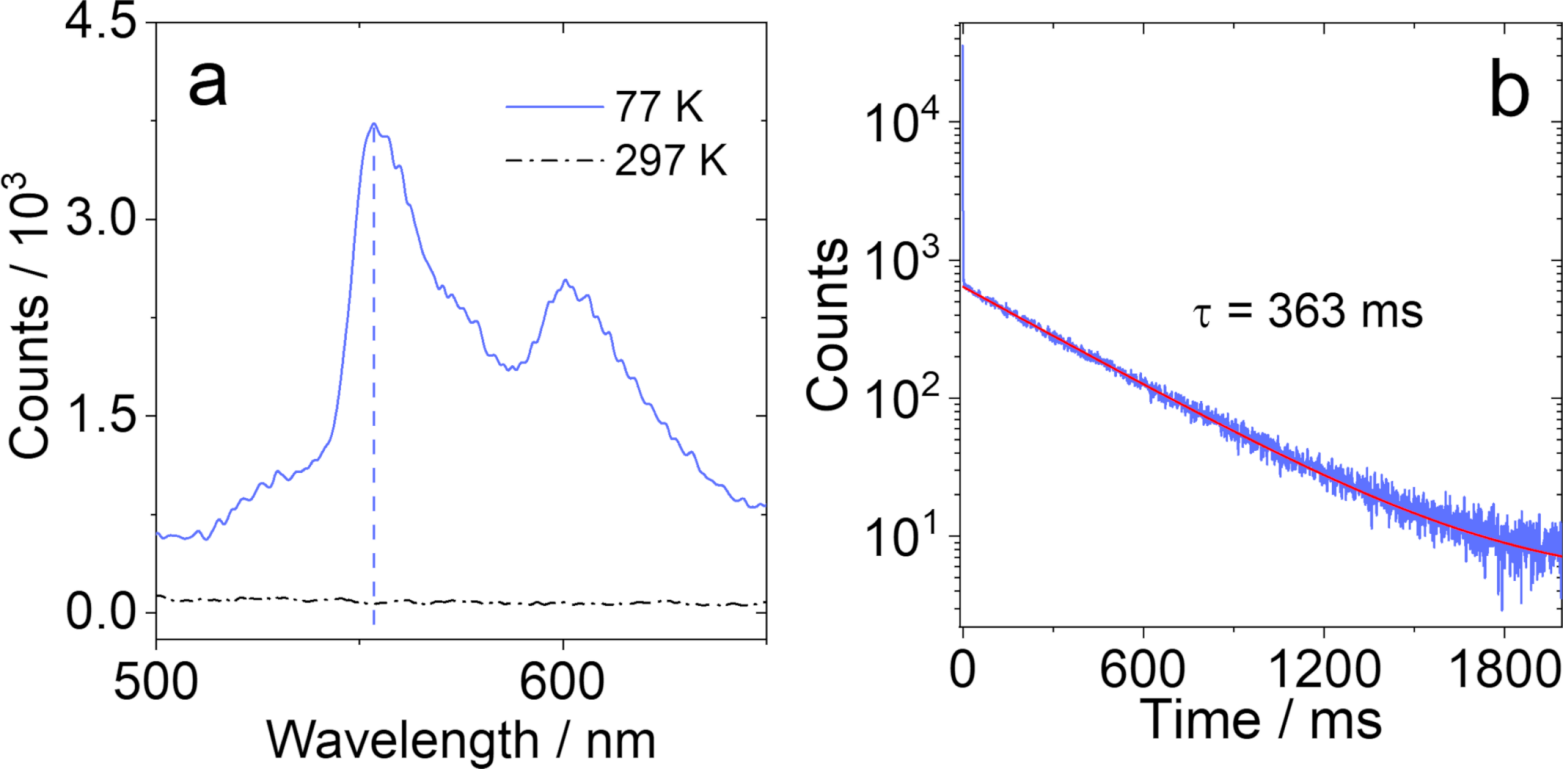
(a) Phosphorescence spectra of **NI-PTZ-O**; (b) decay traces of the phosphorescence of the compounds **NI-PTZ-O**. λ_ex_ = 340 nm, at 77 K, in 2-methyltetrahydrofuran, *c* = 1.0 × 10^−5^ M.

In order to determine the ^3^NI energy in the dyads and in the triad, the phosphorescence emission spectra of **NI-PTZ-O** in frozen solution at 77 K were recorded (Figure 5 and Figure S29 in Supporting Information File 1). No phosphorescence was detected for **NI-PTZ** nor **NI-PTZ_2_**. For **NI-PTZ-O** (Figure 5a), a structured emission with significant vibrational progression was observed in the 520–650 nm range, which is attributed to the ^3^LE state, as the emission band is similar to the one of 4-bromo NI [48]. Thus, the ^3^NI state energy can be approximated to be 2.29 eV from the 0–0 band of the phosphorescence. The ^3^NI energy of **NI-Ph-PTZ**, **NI-PhMe_2_-PTZ**, and **NI-3Br** were determined to be 2.24 eV, 2.27 eV, and 2.27 eV, respectively (see Supporting Information File 1, Figure S29). For **NI-PTZ-O** (Figure 5b), the phosphorescence lifetime of the frozen solution attains 363 ms, which is similar to the phosphorescence lifetime of unsubstituted NI (ca. 410.3 ms) [48]. The phosphorescence lifetime of **NI-Ph-PTZ**, **NI-PhMe_2_-PTZ**, and **NI-3Br** are 432 ms (Figure S30a, Supporting Information File 1), 376 ms (Figure S30b), and 4 ms (Figure S30c), respectively.

The photophysical properties of all compounds are compiled in Table 1. The fluorescence quantum yields of the dyads (1.0% to ≈4.5%) are generally much lower than those of the amino-NI derivatives (60% to ≈70%) [20]. In order to have a preliminary evaluation of the ISC of the compounds, the singlet oxygen quantum yields (Φ_Δ_) were studied in several solvents (Table 1 and Table 2). For **NI-PTZ**, Φ_Δ_ is high in HEX (19%), which is similar to the value previously reported for the analogous dyad **NI-N-PTZ** (Φ_Δ_ = 16%) [20]. However, Φ_Δ_ is lower in CHX (Φ_Δ_ = 8%) and negligible in other solvents with higher polarity. In contrast, **NI-PTZ-O**, **NI-PTZ_2_**, **NI-Ph-PTZ**, and **NI-PhMe_2_-PTZ** show much larger Φ_Δ_ values in HEX (20% to ≈50%), respectively. For **NI-3Br** and **NI-Ph-Br**, Φ_Δ_ are much larger, up to 100% in dichloromethane (DCM) and ACN, likely due to the heavy-atom effect.

**Table 1:** Photophysical parameters of the compounds.

Compound	Solvent	λ_abs_ (nm)^a^	ε^b^	λ_em_ (nm)^c^	*τ*_F_ (ns)^d^	*τ*_P_ (ms)^e^	Φ_F_ (%)^f^	Φ_Δ_ (%)^g^

**NI-PTZ**	CHX	327	1.5	605	9.9 (99.7%) 250 (0.3%)	–^h^	1.9	8
	HEX	325	1.5	644	7.6 (99.8%) 190 (0.2%)		1.7	19
**NI-PTZ-O**	CHX	329	2.0	468	3.4 (99%) 18.1 (1%)	363	2.0	33
	HEX	327	2.0	472	3.1 (99%) 21.2 (1%)		1.2	37
	TOL	335	2.0	542	4.3 (99%) 19.8 (1%)		2.0	53
**NI-PTZ** **_2_**	CHX	295	2.7	630	5.8 (99.6%) 260 (0.4%)	–^h^	1.9	1
	HEX	301	2.4	644	4.9 (99.6%) 200 (0.4%)		1.7	38
**NI-Ph-PTZ**	CHX	332	1.4	490	1.3 (54%) 5.4 (46%)	432	4.2	31
	HEX	332	1.4	482	1.2 (77%) 4.5 (23%)		4.1	28
	TOL	336	1.3	585	0.9 (31%) 14.1 (69%)		4.5	34
**NI-PhMe** **_2_** **-PTZ**	CHX	328	1.6	479	1.6 (56%) 12.9 (44%)	376	1.3	35
	HEX	329	1.6	478	1.2 (51%) 7.2 (49%)		1.0	42
	TOL	333	1.6	595	2.7 (22%) 18.7 (78%)		1.3	40

^a^Maximal UV–vis absorption wavelength, *c* =1.0 × 10−^5^ M, 20 °C; ^b^molar absorption coefficient at absorption maxima, ε: 10^4^ M^−1^ cm^−1^; ^c^emission wavelength; ^d^fluorescence lifetime, λ_ex_ = 340 nm; ^e^phosphorescence lifetime, λ_ex_ = 340 nm, in 2-methyltetrahydrofuran; ^f^fluorescence quantum yields determined, λ_ex_ = 330 nm; ^g^singlet oxygen quantum yields, Ru(bpy)_3_[PF_6_]_2_ was used as standard compound (Φ_Δ_ = 57% in DCM); ^h^not observed.

**Table 2:** Singlet oxygen quantum yields (Φ_Δ_, in%) in different solvents^a^.

Compound	CHX	HEX	TOL	DCM	ACN

**NI-PTZ**	8	19	–^b^	–^b^	–^b^
**NI-PTZ-O**	33	37	53	90	9
**NI-PTZ** **_2_**	1	38	–^b^	–^b^	–^b^
**NI-Ph-PTZ**	31	28	34	–^b^	–^b^
**NI-PhMe** **_2_** **-PTZ**	35	42	40	–^b^	–^b^
**NI-3Br**	33	38	46	100	100
**NI-Ph-Br**	30	39	50	100	100

^a^The *E*_T_ (30) values of the solvents are 30.9 (CHX), 31.0 (HEX), 33.9 (TOL), 40.7 (DCM), and 45.6 (ACN), respectively, in kcal mol^−1^. Singlet oxygen quantum yield (Φ_Δ_) with Ru(bpy)_3_[PF_6_]_2_ as standard (Φ_Δ_ = 0.57 in DCM) in different solvents, λ_ex_ = 437 nm; ^b^not observed.

### Electrochemistry study

**Figure 6:**
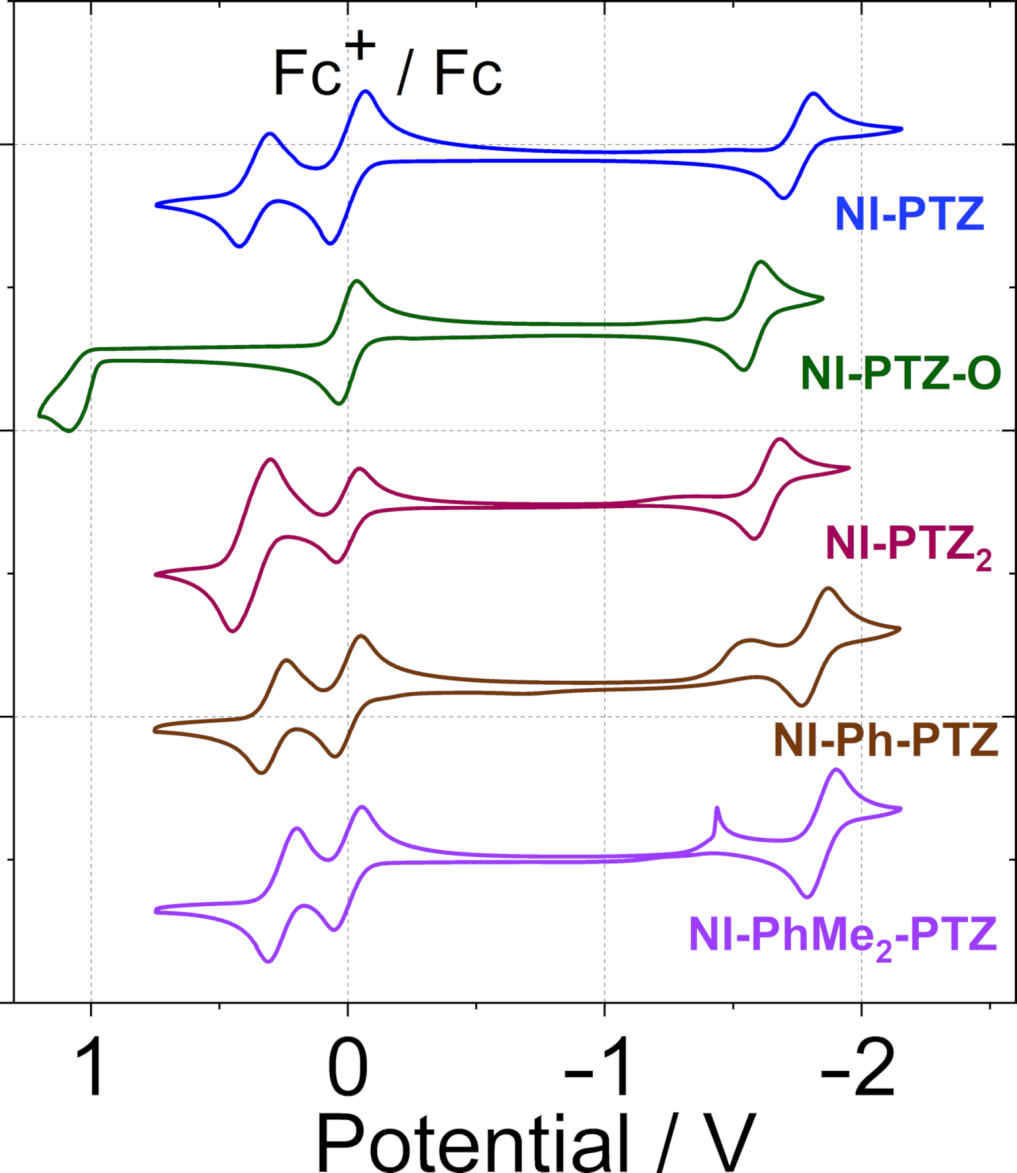
Cyclic voltammograms of the compounds. **NI-PTZ**, **NI-PTZ_2_**, **NI-Ph-PTZ**, and **NI-PhMe_2_-PTZ** were studied in deaerated DCM; **NI-PTZ-O** in deaerated ACN. Ferrocene (Fc) was used as internal reference (set as 0 V in the cyclic voltammograms). 0.10 M Bu_4_NPF_6_ as supporting electrolyte. Scan rates: 100 mV/s, *c* = 1.0 × 10^−3^ M, 20 °C.

The redox potentials of the dyads were studied with cyclovoltammetry (Figure 6, Table 3), and the Gibbs free energy changes of the charge separation (Δ*G*_CS_) and charge separation states energy levels (*E*_CS_) of the compounds were calculated (Table 4). A reversible oxidation wave at +0.36 V (vs Fc/Fc^+^) was observed for **NI-PTZ**, which is attributed to the oxidation of the PTZ units. A reversible reduction wave at −1.75 V (vs Fc/Fc^+^) was observed, which is attributed to the reduction of the NI moiety. These reduction potentials are similar to the ones previously reported for the **NI-N-PTZ** dyad (+0.39, −1.72). However, for **NI-PTZ-O**, an irreversible oxidation wave at +1.09 V (vs Fc/Fc^+^) was observed, which indicates that, upon oxidation, the PTZ moiety becomes a poor electron donor. A reversible reduction wave at −1.57 V was observed, which is cathodically shifted as compared to that of **NI-PTZ**, an expected trend considering the poor electron-donating ability of the oxidized PTZ moiety.

**Table 3:** Electrochemical redox potentials of the compounds.^a^

Compound	*E*(ox)/V	*E*(red)/V

**NI-PTZ** ^b^	+0.36	−1.75
**NI**-**PTZ-O**^c^	+1.09	−1.57
**NI-PTZ** **_2_** ^b^	+0.38	−1.63
**NI-Ph-PTZ** ^b^	+0.29	−1.82
**NI-PhMe** **_2_** **-PTZ** ^b^	+0.25	−1.84
**NI-3Br** ^b^	–^d^	−1.56
**NI-N-PTZ** ^b^	+0.39	−1.72

^a^Cyclic voltammetry in N_2_-saturated solvents containing 0.10 M Bu_4_NPF_6_. Pt electrode as the counter electrode, glassy carbon electrode as the work electrode, ferrocene (Fc/Fc^+^) as the internal reference (set as 0 V in the cyclic voltammograms), and Ag/AgNO_3_ couple as the reference electrode; ^b^in DCM; ^c^in ACN; ^d^not observed.

**Table 4:** Gibbs free energy changes of the charge separation (Δ*G*_CS_) and charge separation states energy (*E*_CS_) of the compounds^a^.

Compound	Δ*G*_CS_ (eV)					*E*_CS_ (eV)			
			
	HEX	TOL	DCM	ACN		HEX	TOL	DCM	ACN

**NI-PTZ** ^b^	−0.38	−0.51	−0.85	−0.95		2.28	2.15	1.81	1.71
**NI-PTZ-O** ^c^	−0.23	−0.29	−0.47	−0.52		2.88	2.81	2.63	2.58
**NI-PTZ** **_2_** ^d^	−0.29	−0.34	−0.93	−1.06		2.39	2.24	1.75	1.62
**NI-Ph-PTZ** ^e^	−0.04	−0.28	−1.10	−1.31		2.98	2.74	1.92	1.71
**NI-PhMe** **_2_** **-PTZ** ^f^	−0.07	−0.32	−1.16	−1.37		3.00	2.75	1.91	1.70

^a^Cyclic voltammetry in deaerated solutions containing 0.10 M Bu_4_NPF_6_. Pt electrode as counter electrode, glassy carbon electrode as working electrode, and Ag/AgNO_3_ couple as the reference electrode; ^b^*E*_00_ = 2.66 eV; ^c^*E*_00_ = 3.11 eV; ^d^*E*_00_ = 2.68 eV; ^e^*E*_00_ = 3.02 eV; ^f^*E*_00_ = 3.07 eV. *E*_00_ (*E*_00_ = 1240/λ) is the singlet state energy of compounds, λ is the wavelength of the crossing point of normalized UV–vis absorption spectra and fluorescence emission spectra.

**Figure 7:**
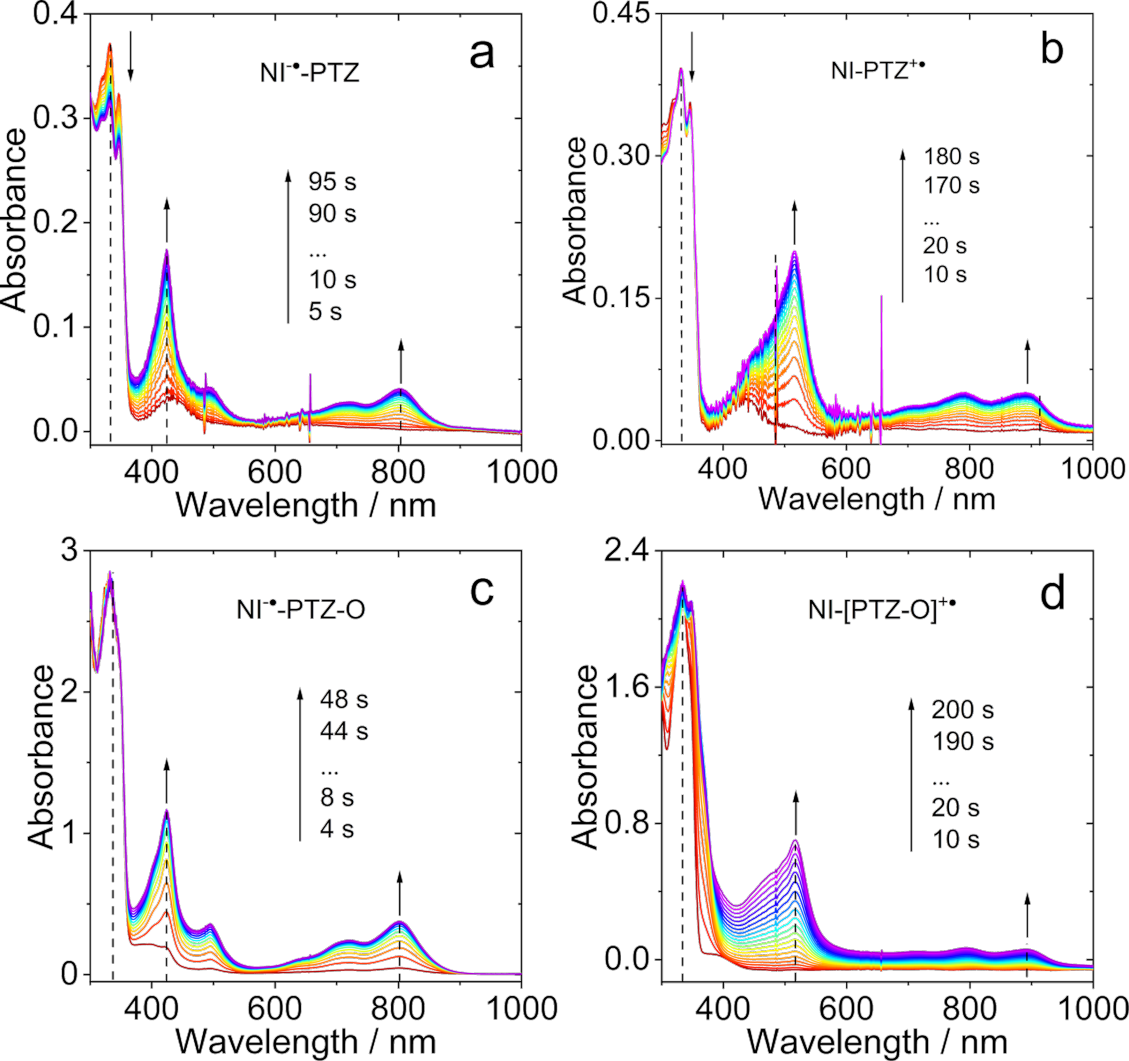
Spectroelectrochemistry traces of the UV–vis absorption spectra for (a) **NI-PTZ** observed from neutral (red) to monoanion (purple) with a potential of −1.83 V applied; (b) **NI-PTZ** observed from neutral (red) to monocationic (purple) at a potential of 0.53 V applied; (c) **NI-PTZ-O** observed from neutral (red) to monoanion (purple) at controlled-potential of −1.80 V; (d) **NI-PTZ-O** observed from neutral (red) to monocationic (purple) at controlled-potential of 2.00 V. In deaerated DCM containing 0.10 M Bu_4_[NPF_6_] as supporting electrolyte and with Ag/AgNO_3_ as reference electrode, 20 °C.

However, the data of **NI-PTZ_2_** shows less intuitive trends, as a reversible reduction wave is observed at −1.63 V (vs Fc/Fc^+^), which is not in line with the presence of two electron-donating PTZ moieties – one would expect, the reduction potential of **NI-PTZ_2_** should be more negative than the one for **NI-PTZ**. Slightly lower oxidation potentials were observed for **NI-Ph-PTZ** and **NI-PhMe_2_-PTZ**. However, the 0.04 eV difference in the oxidation potentials of these two dyads indicates that the different conformational restriction affects the electronic coupling between the NI and PTZ moieties. To help the assignment of the possible CT states in the time-resolved spectra (see later section), the spectroelectrochemistry of the compounds was studied (Figure 7).

For **NI-PTZ**, when a positive potential of +0.53 V (vs Ag/AgNO_3_) was applied, the hallmark absorption bands of the PTZ^•+^ radical cation centered at 516, 794, and 891 nm are observed [20]. These bands are similar to the ones observed for the previously reported **NI-N-PTZ** dyad. Upon a negative potential at −1.83 V (vs Ag/AgNO_3_) applied, the absorption bands of the NI^•−^ radical anion at 424, 492, 720, and 801 nm are observed, which are also similar to the ones of the analogous dyad [20]. For **NI-PTZ-O**, similar NI^•−^ absorption bands were observed. However, the PTZ^•+^ absorption bands of **NI-PTZ-O** are less resolved as compared to those of **NI-PTZ** (Figure 7a). These results indicate the effect of oxidation of the PTZ moiety.

The spectroelectrochemistry traces of **NI-Ph-PTZ**, **NI-PTZ_2_**, and **NI-PhMe_2_-PTZ** were also studied (Supporting Information File 1, Figure S31). For **NI-Ph-PTZ**, the NI^•−^ absorption bands in the 350–600 nm range are less resolved than for **NI-PTZ**. This likely comes from the effect of the π-conjugation of the phenyl ring with the NI moiety in **NI-Ph-PTZ**. In contrast, the PTZ^•+^ absorption band of **NI-Ph-PTZ** resembles the one of **NI-PTZ**, indicating that the spin density of PTZ^•+^ in **NI-Ph-PTZ** is confined on the PTZ moiety, and does not significantly spread on the phenyl linker. The spectroelectrochemistry of **NI-PhMe_2_-PTZ** shows that the NI^•−^ absorption band in this dyad is similar to that of **NI-PTZ**, but not to the one of **NI-Ph-PTZ**. This illustrates the impact of the conformational restriction on the photophysical properties of **NI-PhMe_2_-PTZ**.

We underline that the absorption of the CT states of the dyads may not be the “*simple sum*” of the absorption of the radical cation and the radical anion of the dyads, obtained by the spectroelectrochemistry (Figure 7). The reason is that, in spectroelectrochemistry, one forms either D^•+^–A or D–A^•−^, but not D^•+^–A^•−^. When photoexciting the dyads, however, the CT (D^•+^–A^•−^) state is formed resulting in a different exciton binding energy related to the interaction between the radical anion and cation; this interaction being far from negligible in *compact* dyads.

### Nanosecond transient absorption (ns-TA) spectra

**Figure 8:**
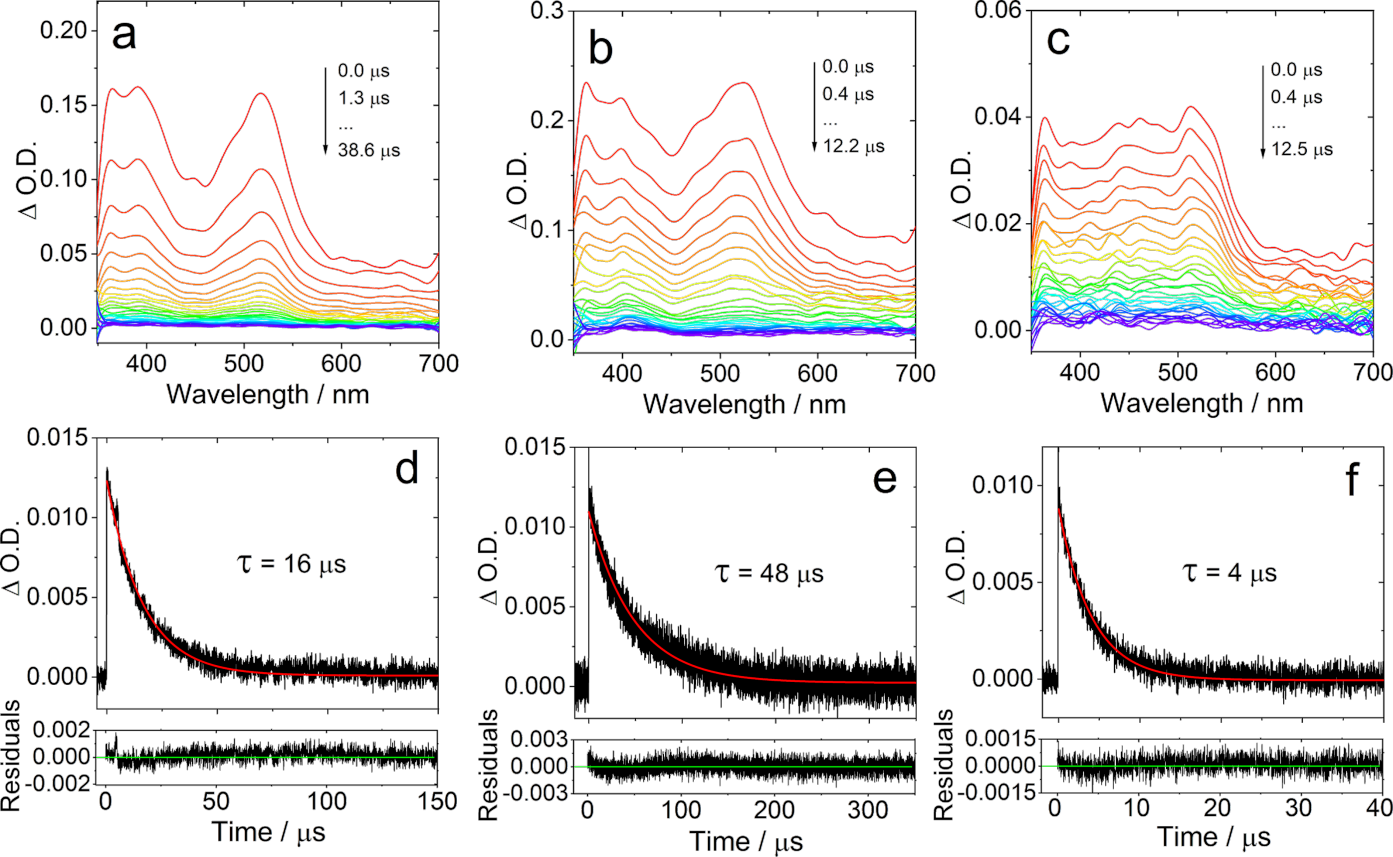
Nanosecond transient absorption spectra of (a) **NI-PTZ** (*c* = 1.5 × 10^−4^ M), (b) **NI-PTZ-O** (*c* = 2.5 × 10^−4^ M), and (c) **NI-PTZ_2_** (*c* = 5.0 × 10^−5^ M). The corresponding decay traces are (d) **NI-PTZ** (*c* = 5.0 × 10^−6^ M) at 520 nm, (e) **NI-PTZ-O** (*c* = 2.0 × 10^−6^ M) at 530 nm, and (f) **NI-PTZ_2_** (*c* = 6.0 × 10^−6^ M) at 510 nm. In deaerated HEX, λ_ex_ = 355 nm, 20 °C.

In order to identify the lowest-lying transient species of the dyads and the triad formed upon photoexcitation, the ns-TA spectra of the compounds were recorded (Figure 8). For the reference **NI-3Br** (Figure S33a, Supporting Information File 1), sharp excited state absorption (ESA) bands centered at 360 and 470 nm were observed in HEX, and a 63 μs lifetime was determined. For **NI-PTZ**, positive absorption bands centered at 360, 470, 390, and 520 nm were observed upon pulsed laser in HEX (Figure 8a). This absorption profile drastically differs from the absorption of the radical anion and the cation (see Figure 7a and b) and that of **NI-3Br**. It is also different from the previously studied analogous dyad, for which a CT state was observed [20]. Thus, we tentatively propose that a ^3^LE state was observed for **NI-PTZ**. The lifetime of the transient species was determined as 16 μs. This lifetime is much longer than that observed for the analogous dyad (2.6 μs), which was assigned to a CT state [20]. This conclusion is supported by the fact that the ^3^LE state energy (2.27 eV) of **NI-PTZ** is slightly lower than its CT state (2.34 eV, approximated from the CT emission band, Figure 3a). Discrepancy results were observed for **NI-PTZ_2_** (Figure 8c) and the lifetime was determined to be 4 μs (Figure 8f). In this case the ^3^LE and CT states share similar energy, the CT state energy is 2.25 eV (approximated from the CT emission band, Figure 3c), and the ^3^LE state energy, 2.27 eV. Observation of a long-lived CT state in compact donor–acceptor dyads is rare [49–51], the CR of ^3^CT→S_0_ is spin forbidden, the ^3^CT state should be longer-lived than the ^1^CT state, which is attributed to the electron spin control effect [15,52–57]. These results confirm that the molecules showing TADF can have either a lowest-lying CT state or a lowest-lying ^3^LE state.

The ns-TA spectra of **NI-PTZ-O** were also studied (Figure 8b). Upon oxidation of the PTZ moiety, the CT state energy increases by 0.6 eV as compared to that of **NI-PTZ** (Table 4). However, the ESA bands of **NI-PTZ-O** are close to those of **NI-PTZ**. Thus, we tentatively assign the transient species as ^3^LE state of **NI-PTZ-O**, and that the ^3^LE state stands as the lowest-lying triplet state with an energy of 2.29 eV based on the low temperature phosphorescence, significantly below the estimated value of 2.88 eV for the CT state (approximated from the CT absorption band, Figure 1). The longer lifetime of **NI-PTZ-O** infers that for **NI-PTZ**, CT state sharing similar energy with ^3^LE may drain the excited state and shorten the triplet state lifetime.

The ns-TA spectra of **NI-Ph-PTZ** and **NI-PhMe_2_-PTZ** were also studied (Figure S32 in Supporting Information File 1). For these two dyads, especially **NI-PhMe_2_-PTZ**, the ESA bands and the triplet state lifetimes (43 μs) are similar to those of **NI-3Br**, and the ^3^LE state is therefore observed in these two dyads. Interestingly, the conformation restriction in **NI-PhMe_2_-PTZ** leads to literally the same ESA bands as in **NI-3Br**. Due to the large separation of the electron donor and acceptor, the CT state energy is increased by ca. 0.7 eV as compared to that of **NI-PTZ** (Table 4). In **NI-PhMe_2_-PTZ**, the CT state energy is 3.02 eV (approximated from the CT emission band, Figure S26 in Supporting Information File 1), and the ^3^LE state energy attains 2.27 eV (approximated from the low temperature phosphorescence). For **NI-Ph-PTZ**, the CT state energy is 2.99 eV (approximated from the CT emission band, Figure 3d), and the ^3^LE state energy is 2.24 eV (approximated from the low temperature phosphorescence). Therefore, it is evident that the ^3^LE state is the lowest-lying triplet state in both **NI-Ph-PTZ** and **NI-PhMe_2_-PTZ**.

It is known that the CT state energy decreases substantially when increasing the solvent polarity. For instance, in **NI-PTZ,** the CT state lies at 2.28 eV in HEX (Table 4), but only at 1.71 eV in ACN, whereas the ^3^LE state is much less sensitive to the polarity and remains at ca. 2.27 eV. Therefore, the ns-TA spectra of the dyads in ACN were studied as well (Figure S34 in Supporting Information File 1). For **NI-PTZ**, positive absorption bands centered at 420 nm and a minor band at 510 nm were observed. These absorption bands are different from the ns-TA of **NI-PTZ** in HEX, however, a feature which is similar to the one found in **NI-N-PTZ** [20]. In other words, the transient species of **NI-PTZ** in ACN upon photoexcitation corresponds to be a CT rather than the ^3^LE state. The CT state lifetime was determined to be 0.37 μs (Figure S34b, Supporting Information File 1), and it is ca. twice longer than the CT state lifetime of **NI-N-PTZ** measured in the same experimental conditions (ca. 0.16 μs, Figure S34d). The CT state lifetime of **NI-PTZ** is rather long, considering the compact dyad structure and the low CT energy in ACN (ca. 1.71 eV). For **NI-PTZ_2_**, the CT state was measured in ACN as well, and a 189 ns lifetime was determined (Figure S35a in Supporting Information File), hinting that the CT state observed in the **NI-PTZ_2_** ns-TA experiments is in fact a ^3^CT state, rather than a ^1^CT state. Indeed, the luminescence studies have shown that the lifetime of ^1^CT state is short with prompt fluorescence on ns timescale.

In comparison, we also studied the triplet state ns-TA spectra of the reference compound **NI-3Br** in ACN (see Supporting Information File 1, Figure S33c), and the data were compared to the ns-TA spectra in HEX. The results show that **NI-3Br** has similar ns-TA spectral features in both HEX and ACN, and the triplet state lifetime are similar in both solvents (63 μs and 118 μs, respectively). Similar results were observed for **NI-Ph-Br,** the triplet state lifetimes are 45 μs and 108 μs in HEX and ACN, respectively (Figure S36, Supporting Information File 1). For **NI-PTZ-O**, ^3^LE and ^3^CT states were observed in ACN, and the lifetime was determined as 71 μs (Figure S35c).

### Computational investigations

**Figure 9:**
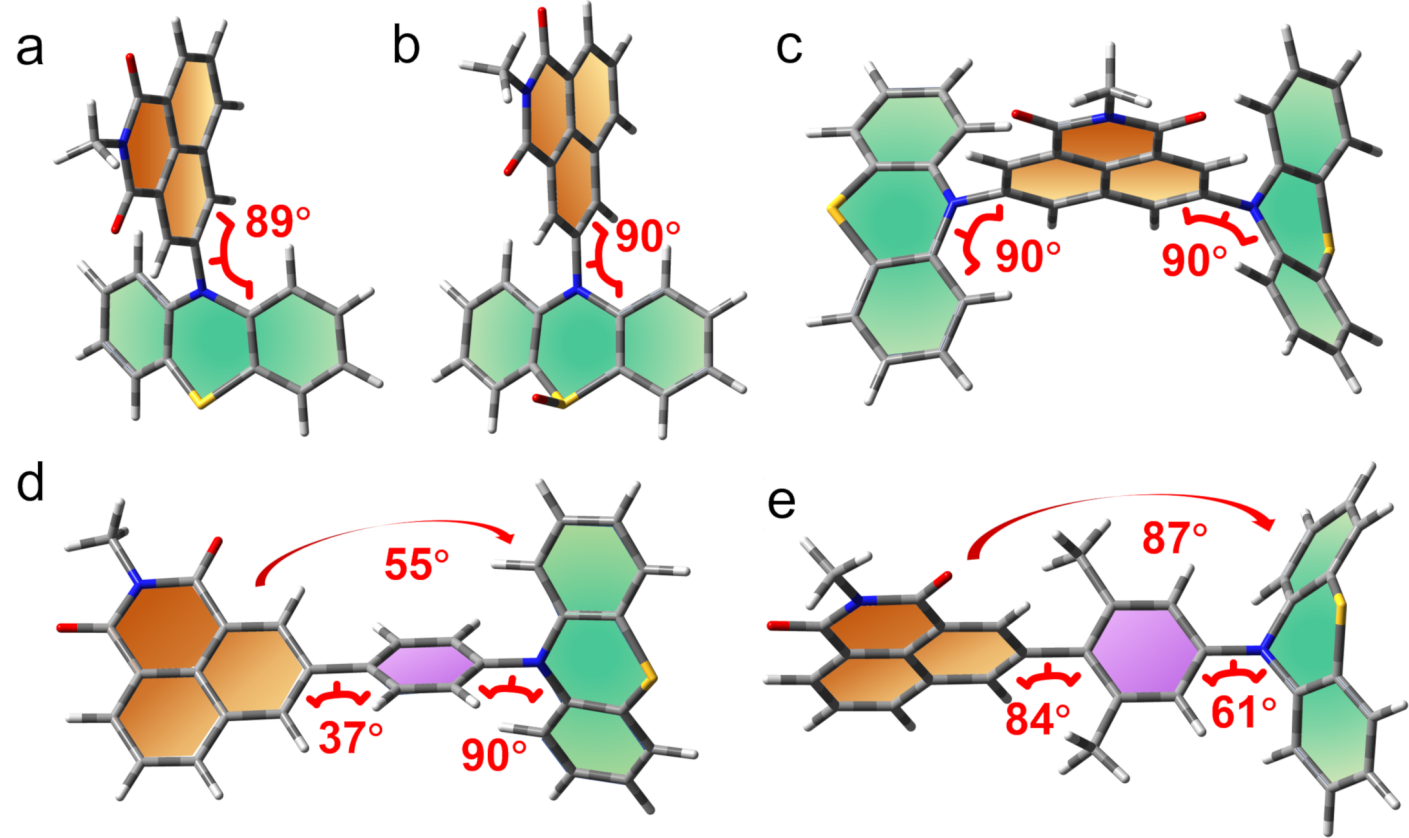
Optimized ground state geometry of (a) **NI-PTZ**, (b) **NI-PTZ-O**, (c) **NI-PTZ_2_**, (d) **NI-Ph-PTZ**, and (e) **NI-PhMe_2_-PTZ**; the green and orange sheets show the planes of the donor and the receptor.

To explain the experimental results, quantum chemical calculations were used to obtain additional insights into both the excited states involved and the photo-deactivation dynamics. First, the ground state geometry of the compounds was optimized (Figure 9). For the compact **NI-PTZ** and **NI-PTZ-O** dyads, the two units adopt almost orthogonal geometry. A similar result was observed for the triad **NI-PTZ_2_**. For the dyads containing a phenyl linker between the NI and the PTZ moieties, the steric hindrance imposed by the methyl substituents on the phenyl linker is significant: the dihedral angle between the NI and the phenyl linker is 37° only in **NI-Ph-PTZ**, but it increases up to 84° in **NI-PhMe_2_-PTZ**. In **NI-Ph-PTZ**, the dihedral angle between the NI and the PTZ is ca. 55°, and it increases up to 87° in **NI-PhMe_2_-PTZ**.

**Figure 10:**
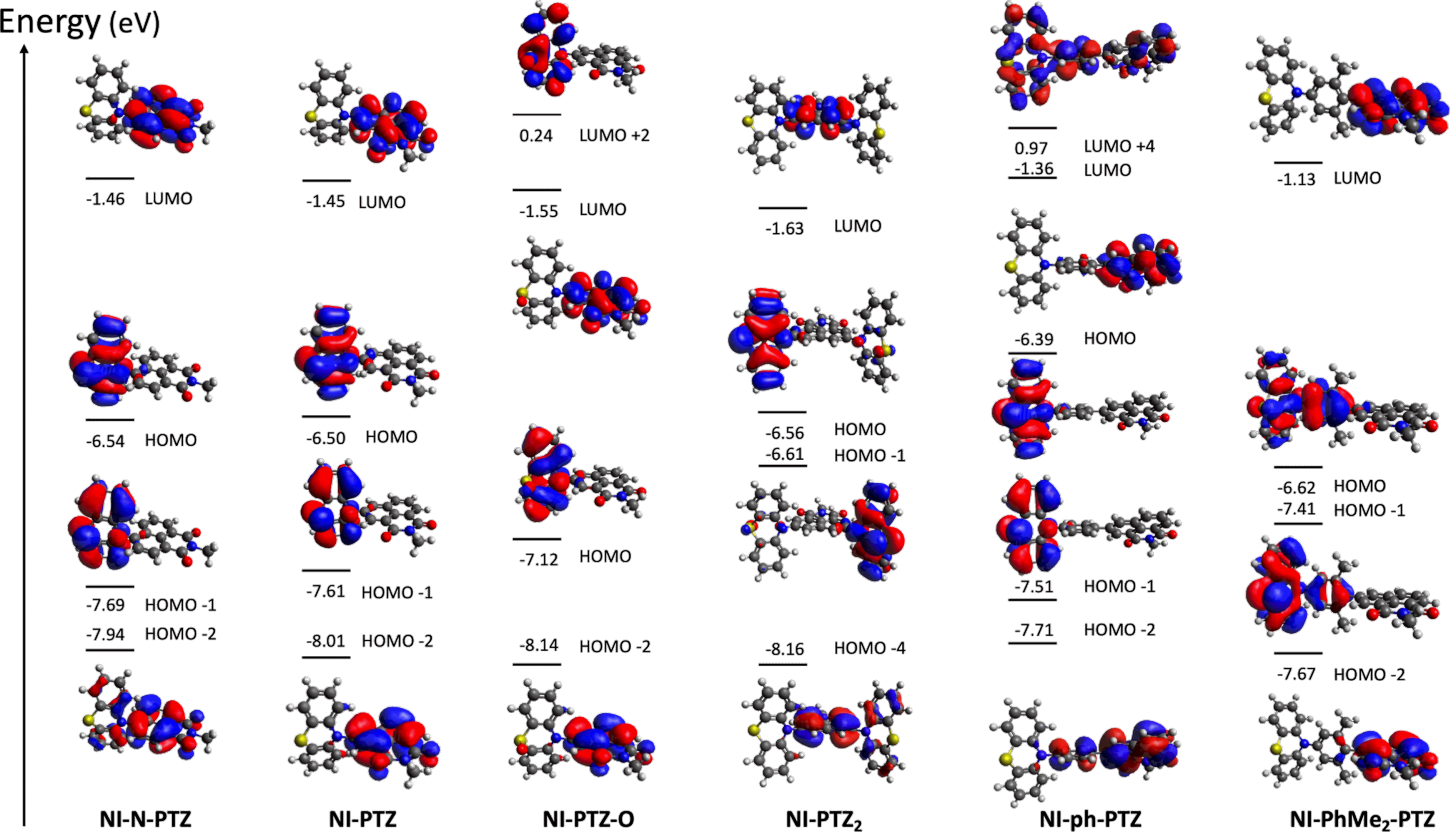
Kohn–Sham frontier molecular orbitals (CAM-B3LYP/6-31G(d) in gas phase) involved in S_1_, T_1_, and T_2_ of **NI-N-PTZ**, **NI-PTZ**, **NI-PTZ-O**, **NI-PTZ_2_**, **NI-Ph-PTZ**, and **NI-PhMe_2_-PTZ**, based on the optimized ground state geometries. An isovalue of 0.02 is used.

As shown in Table S1 (Supporting Information File 1), for all compounds, S_1_ corresponds to a HOMO→LUMO electronic transition (see the molecular orbitals involved in Figure 10) at the Franck–Condon region. The spatial separation between the HOMO and LUMO orbitals along with the very small calculated oscillator strengths (see Table S1 and Table S2, Supporting Information File 1) are clear indicators of a CT character for S_1_. Relaxation on the lowest triplet excited state potential energy surfaces leads to different scenarios for the studied compounds as shown in Table S2: a ^3^CT character is found for the lowest triplet excited state (T_1_) of **NI-PTZ** and a considerable ^3^CT character is found for the T_1_ of **NI-N-PTZ** and **NI-PTZ_2_**. Conversely, for **NI-PhMe_2_-PTZ**, **NI-Ph-PTZ**, and **NI-PTZ-O** a predominant ^3^LE character is found at the T_1_ optimized minima (see Table S2). Note that, at the Frank–Condon region, T_1_ corresponds to a ^3^LE state for all the compounds (see Table S1). Thus, in **NI-PTZ-O**, the relaxation on the T_1_ and T_2_ potential energy surfaces leads to the same state ordering between the ^3^LE and ^3^CT states with respect to the Franck–Condon region, i.e., the ^3^LE state remains the lowest triplet excited state at both the Franck–Condon region and at its optimized geometry. T_1_ corresponds to a ^3^LE state localized on the acceptor ligand and it predominantly involves a HOMO-2-to-LUMO electronic excitation (see Figure 10 for the orbitals). For the same compound, T_2_ corresponds to a HOMO→LUMO transition with a predominant ^3^CT character. The computed triplet energies (see Table S2) are in reasonable agreement with the experimental ones. More in details, the experimental emission maximum for the for ^3^LE band peaks at 2.29 eV, which reasonably matches the computed one (2.02 eV). Note that experimentally, the ^3^LE state was still observed to be the lowest triplet state for **NI-PTZ**. In this respect, the computed energetic difference between T_1_ and T_2_ falls within the typical TD-DFT error bar (ca. 0.3 eV), which explains the difference in state ordering between experiments and calculations. Note also, that the experimental results point to a small energetic difference between the ^3^LE and ^3^CT states, as triplet state lifetime is shorter than the pristine ^3^NI state.

For completeness, Table S3 (Supporting Information File 1) reports the adiabatic and vertical energies of the ^3^LE and ^3^CT states for **NI-PTZ** and **NI-PTZ-O** (as representative cases of the different photophysical scenarios within the series of compounds) along with the state ordering at the ^1^CT optimized geometries. Comparing **NI-PTZ-O** with **NI-PTZ** at their ^1^CT optimized geometries, a different ordering of the triplet excited states is obtained. More in details, for **NI-PTZ-O**, both ^3^LE (2.63 eV) and ^3^CT (3.09 eV) are lower in energy than the ^1^CT state (3.12 eV), but for **NI-PTZ** the ^3^LE state (2.74 eV) is higher in energy than the ^1^CT state (2.60 eV), while ^3^CT remains slightly lower in energy (2.57 eV). This has some important consequences on the TADF mechanisms.

Table 5 lists spin–orbit couplings matrix elements (SOCMEs) between ^1^CT, ^3^LE, and ^3^CT states. The SOCMEs between ^1^CT and ^3^CT are small, 0.03 cm^−1^ at most, which was expected as they involve the same electronic transitions. Conversely, the SOCMEs between ^1^CT and ^3^LE amount up to 0.47 cm^−1^ in the case of **NI-PTZ-O**. For **NI-PTZ** the computed ISC rate from ^1^CT towards ^3^LE attains to 2.92 × 10^7^ s^−1^, which is two orders of magnitude larger than the ISC rate towards ^3^CT (8.51 × 10^5^ s^−1^). The RISC from ^3^CT (2.79 ×10^5^ s^−1^) back to ^1^CT is large enough to compete with other photodeactivation processes, so TADF is likely. In order to further proof this, we also calculated the phosphorescence rate (*k*_phos_; see Experimental section, Computational details). The computed *k*_phos_ amounts up to 6.85 × 10^−1^ s^−1^, thus confirming that phosphorescence from ^3^CT is not competitive with RISC, also in agreement with the experimental evidences. For **NI-PTZ-O**, the fastest rate of ISC is found for the ^1^CT→^3^LE transition (4.12 × 10^6^ s^−1^). Conversely, the RISC process from the ^3^LE state is very unlikely to occur, as the back process to ^1^CT is characterized by a large energy gap (ca. 0.49 eV, see Table S3 in Supporting Information File 1), thus explaining the different TADF properties experimentally measured for **NI-PTZ-O** and **NI-PTZ**. The analysis of the computed reorganization energies for selected (R)ISC processes of **NI-PTZ-O** and **NI-PTZ** and the activation barriers derived from these values clearly highlight the same trends obtained with the rate calculations [58]. For instance, a negligible RISC decay rate for the ^3^LE→^1^CT process in **NI-PTZ-O** roots on a large adiabatic energy difference and an even larger reorganization energy (0.75 and 1.92 eV, respectively), which lead to an activation barrier of ca. 3.42 eV (see details in Supporting Information File 1). Conversely, the sizable value of the RISC rate for the for the ^3^LE→^1^CT process in **NI-PTZ** roots on a small adiabatic energy difference and a similar value for the reorganization energy (0.23 vs 0.53 eV, respectively). This results in an activation barrier for the RISC process of ca. 0.076 eV, which is small enough to enable the RISC process at room temperature.

**Table 5:** Calculated SOCMEs (cm^−1^) and computed rates (s^−1^) along with selected reorganization energies (values between brackets in eV) for the (R)ISC processes **NI-PTZ** and **NI-PTZ-O**.

Molecule	SOCME (cm^−1^)			*k*_ISC_ (s^−1^)^a^			*k*_RISC_ (s^−1^)^a^	
					
	^1^CT–^3^LE	^1^CT–^3^CT		^1^CT–^3^LE	^1^CT–^3^CT		^1^CT–^3^LE	^1^CT–^3^CT

**NI-PTZ**	0.33	0.03		2.92 × 10^7^	8.51 × 10^5^		3.79 × 10^5^ (0.53)^b^	2.79 × 10^5^
**NI-PTZ-O**	0.47	0.02		4.12 × 10^6^	1.23 × 10^5^		0 (1.92)^b^	8.13 × 10^4^

^a^Rates obtained by making use of the vertical hessian model as implemented in FCclasses. All computations are performed in the gas phase. ^b^Reorganization energies (in eV) for the RISC processes.

**Scheme 2:**
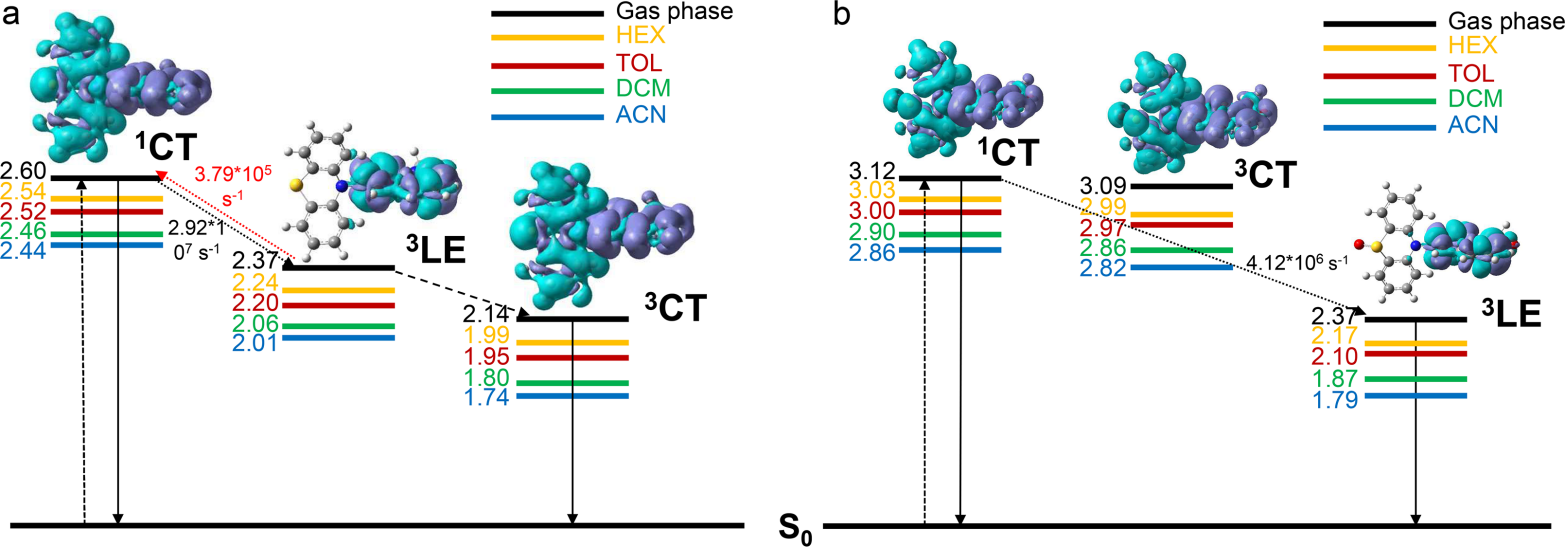
Jablonski diagram of (a) **NI-PTZ** and (b) **NI-PTZ-O**, including electron density difference (EDD) at T_2_ (^3^LE) geometry and computed in gas phase. EDD isovalues are 0.02 au. The cyan and blue lobes of the EDD indicate increase and decrease of electron density, respectively. The energies in eV were obtained with single point calculations on optimized S_1_, T_1_, and T_2_ geometry in HEX, DCM, TOL, and ACN.

In Scheme 2, a summary of the photodeactivation pathways for **NI-PTZ** and **NI-PTZ-O** is presented. In addition, the impact of solvent on the energy of the states is included. When moving from apolar to polar solvents a little increase in the energy gap between ^1^CT and ^3^LE is observed (amounting to up to 0.13 eV in acetonitrile), which is mostly due to the stabilization of ^3^LE (0.23 eV for **NI-PTZ** and 0.30 eV for **NI-PTZ-O**) while the ^1^CT state is only stabilized by 0.10 eV and 0.17 eV, respectively for both **NI-PTZ** and **NI-PTZ-O**. Comparing the computed energies (Scheme 2) with the experimental energies (see Table 4) results in a reasonable agreement, especially in a polar solvent (ACN), where the experimental energy of **NI-PTZ** for the ^1^CT state is 1.71 eV and the computed energy, 1.74 eV. For **NI-PTZ-O** the experimental energy of the ^3^CT in ACN is 2.58 eV, while the computed energy, 2.82 eV, deviates more from the experiment (0.24 eV), but remains in a reasonable agreement with the measured values. The CT character of the ^1^CT and ^3^CT states is visible in the electronic density difference (EDDs) plots shown in Scheme 2, which clearly indicates the flow of electron density from the PTZ to the NI moieties. Conversely, the LE states are fully localized within the acceptor fragment.

## Conclusion

In order to study the impact of the energy matching between the charge-transfer (CT) and localized triplet excited (^3^LE) states on the thermally activated delayed fluorescence (TADF), a series of compact electron donor–acceptor dyads and a triad were prepared. In the dyads and the triad, naphthalimide (NI) was used as electron acceptor and phenothiazine (PTZ) as electron donor. The NI and PTZ moieties are either directly connected at the 3-position of NI and the *N*-position of the PTZ moiety via a C–N single bond, or connected through an intervening phenyl linker. Tuning the electron-donating ability of the PTZ unit, and consequently the CT state energy, was achieved by its oxidation to yield the corresponding sulfoxide. The conformation restriction was imposed through introducing *ortho*-methyl substituents on the phenyl linker. TADF was observed for the dyads and the triad, indicated by the biexponential fluorescence decay, for instance 16.0 ns (99.9%)/14.4 μs (0.1%). Singlet oxygen photosensitizing experiments showed that the Φ_Δ_ of **NI-PTZ** is moderate in HEX (Φ_Δ_ = 19%), but that upon oxidation of the PTZ unit in the dyad much larger values were observed for the resulted dyad **NI-PTZ-O** (up to 90% in DCM) due to the increase of the CT state energy. In nanosecond transient absorption spectra in HEX, in general a ^3^LE state was observed (lifetime: 16–48 μs). For all the compounds, CT emission bands were observed in HEX. In polar solvents, CT state was observed for **NI-PTZ**, **NI-N-PTZ**, and **NI-PTZ_2_** (lifetime: 156–365 ns). Computational investigations unambiguously unraveled the origins of TADF in **NI-PTZ**. Our investigations also underpin the striking photophysical behavior of **NI-PTZ-O** (i.e., phosphorescence and absence of TADF) which originates from a different excited state ordering between the ^3^CT and ^3^LE states in **NI-PTZ** and **NI-PTZ-O**. The tuning of the energy order of the ^3^CT and ^3^LE state is achieved by the feasible oxidation of the PTZ unit in the dyads, while the other factors kept intact; this approach may become a promising methodology in the study of the entangled excited states and the photophysical processes of TADF molecules based on the electron donor–acceptor dyads structure motif. These studies are also useful to understand the subtle entanglement of the ^1^LE, ^1^CT, ^3^CT, and ^3^LE states of TADF based on electron donor–acceptor dyads, as well as the photophysical processes of these dyads upon photoexcitation.

## Experimental

### General methods

All the chemicals used in synthesis are analytical pure and were used as received without purification. UV–vis absorption spectra were measured on a UV-2550 spectrophotometer (Shimadzu Ltd., Japan). Fluorescence emission spectra were recorded with an FS5 spectrofluorometer (Edinburgh instruments Ltd., U.K.). Fluorescence quantum yields (Φ_F_) were measured by an absolute photoluminescence quantum yield spectrometer (Quantaurus-QY Plus C13534-11, Hamamatsu Ltd., Japan). Luminescence lifetimes of compounds were recorded with an OB920 luminescence lifetime spectrometer (Edinburgh Instruments Ltd., U.K.). **NI-PTZ**, **NI-PTZ-O**, **NI-PTZ_2_**, **NI-Ph-PTZ**, and **NI-PhMe_2_-PTZ** were prepared according to the literature methods [21,59].

### Synthesis of **NI-PTZ**

Compound **NI-PTZ** was synthesized in a manner similar to [21]. Under N_2_ atmosphere, **NI-3Br** (468.0 mg, 1.209 mmol), phenothiazine (289.0 mg, 1.452 mmol), Pd(OAc)_2_ (49.2 mg, 0.219 mmol) and sodium *tert*-butoxide (760.0 mg, 7.908 mmol) were dissolved in dry toluene (22 mL). Then tri-*tert*-butylphosphine tetrafluoroborate (66.4 mg, 0.229 mmol) was added. The mixture was refluxed and stirred for 8 h under N_2_. After cooling, water (20 mL) was added, and the mixture was extracted with ethyl acetate (80 mL). The organic layer was separated and washed with water and brine (3 × 30 mL), respectively. The organic layer was combined, dried over anhydrous Na_2_SO_4_, and the solvent was evaporated under reduced pressure. The crude product was purified by column chromatography (silica gel, DCM/PE 1:3, v:v). Compound **NI-PTZ** was obtained as orange solid. Yield: 570 mg (93.1%). Mp 61.9–62.7 °C; ^1^H NMR (CDCl_3_, 400 MHz) δ 0.88 (t, *J* = 14.17 Hz, 3H), 0.94 (t, *J* = 14.89 Hz, 3H), 1.29–1.34 (m, 4H), 1.36–1.41 (m, 4H), 1.93–1.97 (m, 1H), 4.07–4.17 (m, 2H), 6.61 (d, *J* = 7.99 Hz, 2H), 6.97–7.03 (m, 4H), 7.22 (d, *J* = 7.63 Hz, 2H), 7.75–7.77 (m, 1H), 8.08 (s, 1H), 8.13 (d, *J* = 8.18 Hz, 1H), 8.57 (s, 1H), 8.58 (s, 1H); ^13^C NMR (CDCl_3_, 125 MHz) δ 164.40, 163.96, 141.60, 138.00, 133.34, 130.84, 130.44, 129.54, 127.72, 127.22, 126.37, 125.31, 125.01, 124.16, 122.85, 119.77, 44.36, 37.97, 30.77, 29.70, 28.71, 24.08, 23.10, 10.66; HRMS–MALDI (*m*/*z*): [M + H]^+^ calcd for C_32_H_30_N_2_O_2_S, 506.2028; found, 506.2023.

### Synthesis of **NI-PTZ-O**

Compound **NI-PTZ-O** was synthesized in a manner similar to [59]. Compound **NI-PTZ** (200 mg, 0.4 mmol) was dissolved in glacial acetic acid (28 mL), H_2_O_2_ (8.2 mL, 30%, 6.5 mmol) was added dropwise. The mixture was stirred at 40 °C overnight. The mixture was poured into water and the pH of the mixture was brought to 7 with a saturated aqueous solution of Na_2_CO_3_. After cooling, water (20 mL) was added, and the mixture was extracted with ethyl acetate (80 mL). The organic layer was separated and washed with water and brine solution (3 × 30 mL), respectively. The organic layer was dried over anhydrous Na_2_SO_4_ and the solvent was evaporated under reduced pressure. The crude product was purified by column chromatography (silica gel, DCM/MeOH 50:1, v:v). **NI-PTZ-O** was obtained as yellow solid. Yield: 180 mg (87.2%). Mp 176.2–177.2 °C; ^1^H NMR (CDCl_3_, 400 MHz) δ 0.88–0.98 (m, 6H), 1.27–1.43 (m, 8H), 1.95–2.01 (m, 1H), 4.11–4.22 (m, 2H), 6.67 (d, *J* = 8.26 Hz, 2H), 7.29 (d, *J* = 7.38 Hz, 2H), 7.38–7.42 (m, 2H), 7.88–7.92 (m, 1H), 8.05 (d, *J* = 7.38 Hz, 2H), 8.28 (d, *J* = 8.00 Hz, 1H), 8.42 (s, 1H), 8.62 (s, 1H), 8.76 (d, *J* = 7.13 Hz, 1H); ^13^C NMR (CDCl_3_, 125 MHz) δ 164.51, 139.11, 138.73, 138.17, 133.68, 133.51, 132.44, 131.95, 131.22, 130.44, 127.36, 123.27, 122.83, 121.64, 44.27, 37.98, 30.75, 28.71, 24.07, 23.10, 20.81, 10.66; HRMS–MALDI (*m*/*z*): [M + H]^+^ calcd for C_32_H_30_N_2_O_3_S, 522.1977; found, 523.2050.

### Synthesis of **NI-PTZ_2_**

Compound **NI-PTZ_2_** was synthesized in a manner similar to [21]. Under N_2_ atmosphere, compound **1** (190.0 mg, 0.409 mmol), phenothiazine (294.5 mg, 1.478 mmol), Pd(OAc)_2_ (36 mg, 0.160 mmol), and sodium *tert*-butoxide (317.0 mg, 3.299 mmol) were dissolved in dry toluene (12 mL). Then, tri-*tert*-butylphosphine tetrafluoroborate (53.0 mg, 0.183 mmol) was added. The mixture was refluxed and stirred for 24 h under N_2_. After cooling, water (20 mL) was added, and the mixture was extracted with ethyl acetate (80 mL). The organic layer was separated and washed with water and brine (3 × 30 mL), respectively. The organic layer was dried over anhydrous Na_2_SO_4_ and the solvent was evaporated under reduced pressure. The crude product was purified by column chromatography (silica gel, DCM/PE 1:4, v:v). Compound **NI-PTZ_2_** was obtained as orange solid. Yield: 230 mg (80.0%). Mp 100.1–101.0 °C; ^1^H NMR (CDCl_3_, 400 MHz) δ 0.86–0.94 (m, 6H), 1.30–1.41 (m, 8H), 1.90–1.96 (m, 1H), 4.03–4.14 (m, 2H), 6.77 (m, 4H), 7.01–7.11 (m, 8H), 7.25 (d, *J* = 1.51 Hz, 2H), 7.86 (d, *J* = 2.12 Hz, 2H), 8.46 (d, *J* = 2.12 Hz, 2H); ^13^C NMR (CDCl_3_, 125 MHz) δ 163.94, 142.79, 135.00, 127.96, 127.82, 126.96, 126.23, 124.62, 123.50, 121.13, 44.52, 37.95, 30.75, 29.70, 28.72, 24.06, 23.10, 10.66; HRMS–MALDI (*m*/*z*): [M + H]^+^ calcd for C_44_H_37_N_3_O_2_S_2_, 703.2327; found, 703.2322.

### Synthesis of **NI-Ph-PTZ**

Compound **NI-Ph-PTZ** was synthesized in a manner similar to [21]. Under N_2_ atmosphere, compound **2** (51 mg, 0.110 mmol), phenothiazine (26.3 mg, 0.132 mmol), Pd(OAc)_2_ (4.5 mg, 0.020 mmol), and sodium *tert*-butoxide (69.1 mg, 0.720 mmol) were dissolved in dry toluene (3 mL). Then, tri-*tert*-butylphosphine tetrafluoroborate (6.1 mg, 0.021 mmol) was added. The mixture was refluxed and stirred for 8 h under N_2_. After cooling, water (20 mL) was added, and the mixture was extracted with ethyl acetate (80 mL). The organic layer was separated and washed with water and brine solution (3 × 30 mL), respectively. The organic layer was dried over anhydrous Na_2_SO_4_ and the solvent was evaporated under reduced pressure. The crude product was purified by column chromatography (silica gel, DCM/PE 6:1, v:v). The product **NI-Ph-PTZ** was obtained as yellow solid. Yield: 40 mg (62.4%). Mp 126.7–128.3 °C; ^1^H NMR (CDCl_3_, 400 MHz) δ 0.94–0.97 (m, 6H), 1.33–1.42 (m, 8H), 1.97 (s, 1H), 4.12–4.22 (m, 2H), 6.41 (d, *J* = 6.75 Hz, 2H), 6.87–6.93 (m, 4H), 7.10 (d, *J* = 6.63 Hz, 2H), 7.54 (d, *J* = 7.13 Hz, 2H), 7.80–7.83 (m, 1H), 7.99 (d, *J* = 6.63 Hz, 2H), 8.29 (d, *J* = 8.13 Hz, 1H), 8.46 (s, 1H), 8.62 (d, *J* = 6.38 Hz, 1H), 8.94 (s, 1H); ^13^C NMR (CDCl_3_, 125 MHz) δ 164.54, 139.04, 138.63, 133.96, 132.17, 131.18, 130.58, 129.86, 127.54, 127.46, 126.82, 123.47, 122.80, 117.10, 44.25, 38.01, 30.80, 29.36, 28.75, 24.11, 23.09, 10.69; HRMS–MALDI (*m*/*z*): [M + H]^+^ calcd for C_38_H_34_N_2_O_2_S, 582.2341; found, 582.2336.

### Synthesis of **NI-PhMe_2_-PTZ**

Compound **NI-PhMe_2_-PTZ** was synthesized in a manner similar to [21]. Under N_2_ atmosphere, compound **3** (142 mg, 0.290 mmol), phenothiazine (69.3 mg, 0.348 mmol), Pd(OAc)_2_ (11.7 mg, 0.052 mmol), and sodium *tert*-butoxide (182.4 mg, 1.898 mmol) were dissolved in dry toluene (5 mL). Then, tri-*tert*-butylphosphine tetrafluoroborate (16.1 mg, 0.055 mmol) was added. The mixture was refluxed and stirred for 8 h under N_2_. After cooling, water (20 mL) was added and the mixture was extracted with ethyl acetate (80 mL). The organic layer was separated and washed with water and brine solution (3 × 30 mL), respectively. The organic layer was dried over anhydrous Na_2_SO_4_ and the solvent was evaporated under reduced pressure. The crude product was purified by column chromatography (silica gel, DCM/PE 1:5, v:v). The product **NI-PhMe_2_-PTZ** was obtained as yellow solid. Yield: 50 mg (28.3%). Mp 121.2–122.4 °C; ^1^H NMR (CDCl_3_, 400 MHz) δ 0.89–0.97 (m, 6H), 1.33–1.43 (m, 8H), 1.94–2.01 (m, 1H), 1.98 (t, 1H), 2.12 (s, 6H), 4.11–4.22 (m, 2H), 6.36 (s, 2H), 6.91 (s, 3H), 7.00–7.05 (m, 3H), 7.20 (s, 2H), 7.80–7.84 (m, 1H), 8.12 (s, 1H), 8.25 (d, *J* = 8.13 Hz, 1H), 8.50 (s, 1H), 8.65 (d, *J* = 7.26 Hz, 1H); ^13^C NMR (CDCl_3_, 125 MHz) δ 164.55, 139.69, 139.45, 138.85, 133.63, 132.71, 132.03, 131.20, 127.35, 126.84, 123.29, 122.89, 116.24, 44.24, 38.01, 30.78, 29.70, 28.71, 24.11, 23.09, 21.19, 10.67; HRMS–MALDI (*m*/*z*): [M + H]^+^ calcd for C_40_H_38_N_2_O_2_S, 610.2654; found, 610.2649.

### Electrochemical studies

The cyclic voltammetry curves were recorded with a CHI610D electrochemical workstation (CHI instruments, Inc., Shanghai, China) using N_2_-purged saturated solutions (**NI-PTZ**, **NI-PTZ_2_**, **NI-Ph-PTZ**, and **NI-PhMe_2_-PTZ** in deaerated dichloromethane, **NI-PTZ-O** in deaerated acetonitrile) containing 0.10 M Bu_4_NPF_6_ as a supporting electrolyte, a platinum electrode as counter electrode, a glassy carbon electrode as working electrode, and the Ag/AgNO_3_ (0.1 M in ACN) couple as the reference electrode. The ferrocenium/ferrocene (Fc^+^/Fc) redox couple was used as an internal reference. Spectroelectrochemistry was performed using a 0.1 cm path length quartz electrochemical cell equipped with gauze platinum as working electrode, a platinum wire as counter electrode, and Ag/AgNO_3_ as reference electrode. Bu_4_N[PF_6_] was used as the supporting electrolyte. The potential was regulated with a CHI610D electrochemical workstation (CHI instruments, Inc., Shanghai, China), and the spectra were recorded with an Agilent 8453E UV–vis spectrophotometer (Agilent Technologies Inc., USA). Samples were deaerated with N_2_ for ca. 5 min before measurement and the N_2_ atmosphere was kept during the measurements.

### Nanosecond transient absorption spectroscopy

The nanosecond transient absorption spectra were recorded on a LP920 laser flash photolysis spectrometer (Edinburgh Instruments, Ltd., U.K.). The data (kinetic decay traces and the transient difference absorption spectra) were analyzed with the L900 software. All samples were deaerated with N_2_ for ca. 15 min in collinear configuration of the pump and probe beams measurements before measurement, and excited with a nanosecond pulsed laser (Quantel Nd: YAG nanosecond pulsed laser). The typical laser power is 65 mJ per pulse.

### Computational study

The ground state (S_0_) geometries of compounds **NI-N-PTZ**, **NI-PTZ**, **NI-PTZ-O**, **NI-PTZ_2_**, **NI-Ph-PTZ**, and **NI-PhMe_2_-PTZ**, were optimized with Density Functional Theory (DFT) using the CAM-B3LYP rane-separated hybrid functional in combination with the 6-31G(d) atomic basis set [60]. The excited states geometries of S_1_, T_1_, and T_2_ were optimized with time-dependent DFT (TD-DFT) in its Tamm–Dancoff approximation (TDA) using the same functional and basis sets as in the ground-state optimizations [61]. TDA-TD-DFT is preferred over standard (or “full”) TD-DFT as the former is often more reliable for triplet excited states. Solvent effects were included using the polarizable continuum model (PCM) [62–64]. The above calculations were performed with Gaussian 16 [65]. The spin–orbit matrix elements (SOCME) between the manifold of singlet and triplet excited states were calculated with the pSOC-TD-DFT method, as implemented in ORCA 5.0.2. [61,66–67]. The latter calculations were also performed with CAM-B3LYP using the TDA approximation [61]. In the pSOC-TD-DFT calculations, relativistic effects were considered using the zero-order relativistic approximation (ZORA) and the ZORA-*def2*-TZVP basis sets [66,68–69]. pSOC-TD-DFT calculations were performed at the S_1_ optimized geometry [66] for the rate calculations and at the T_1_ optimized geometry in the case of the phosphorescence rate calculation. The rates of intersystem crossing (ISC) and reverse ISC (RISC) along with the reorganization energies were calculated with FCclasses making use of the vertical hessian vibronic model [70–71]. For the phosphorescence rate (*k*_phos_) calculation a simplified Einstein-based expression, i.e,


[1] $$k_{r}\approx \frac{v^{2}\cdot f}{1.5}$$

was used, where ν is the energy gap between the involved states (in cm^−1^) and *f* is the oscillator strength for the T_1_→S_0_ process, which was obtained from the pSOC-TD-DFT calculations.

## Supporting Information

File 1 General experimental methods, ^1^H NMR, ^13^C NMR, HRMS spectra of the compounds, theoretical computation data and the photophysical data.
