# Supplementary material for: Naphthalimide-phenothiazine dyads: effect of conformational flexibility and matching of the energy of the charge-transfer state and the localized triplet excited state on the thermally activated delayed fluorescence
Source: Beilstein J Org Chem. 2022 Oct 11;18:1435–53. doi: 10.3762/bjoc.18.149 (PMC9577389; doi:10.3762/bjoc.18.149)
Supplement: File 1 — General experimental methods, 1H NMR, 13C NMR, HRMS spectra of the compounds, theoretical computation data and the photophysical data. [file Beilstein_J_Org_Chem-18-1435-s001.pdf]

BEILSTEIN JOURNAL OF ORGANIC CHEMISTRY

# Supporting Information

for

# Naphthalimide-phenothiazine dyads: effect of conformational flexibility and matching of the energy of the charge-transfer state and the localized triplet excited state on the thermally activated delayed fluorescence

Kaiyue Ye, Liyuan Cao, Davita M. E. van Raamsdonk, Zhijia Wang, Jianzhang Zhao, Daniel Escudero and Denis Jacquemin

*Beilstein J. Org. Chem.* **2022,** *18,* 1435–1453. doi:10.3762/bjoc.18.149

# General experimental methods, $^{1}$H NMR, $^{13}$C NMR, HRMS spectra of the compounds, theoretical computation data and the photophysical data

License and Terms: This is a supporting information file under the terms of the Creative Commons Attribution License (https://creativecommons.org/licenses/by/4.0). Please note that the reuse, redistribution and reproduction in particular requires that the author(s) and source are credited and that individual graphics may be subject to special legal provisions.
The license is subject to the *Beilstein Journal of Organic Chemistry* terms and conditions: (https://www.beilstein-journals.org/bjoc/terms)

## Table of contents

1. General information······S2

2. Synthesis of the compounds······S2

3. Molecular structure characterization data······S5

4. Steady state UV−vis absorption and luminescence spectra······S20

5. Fluorescence lifetimes······S22

6. Phosphorescence spectra······S22

7. Phosphorescence lifetimes······S23

8. Spectroelectrochemistry measurement······S24

9. Nanosecond transient absorption spectroscopy······S25

10.Theoretical computation······S30

# 1. General information

All the chemicals used in synthesis are analytically pure and were used as received. Solvents were dried and distilled prior to use. $^{1}H$ and $^{13}C$ NMR spectra were recorded on Bruker Avance spectrometers (400/500/600 MHz). $^{1}H$ and $^{13}C$ chemical shifts are reported in parts per million (ppm) relative to TMS, with the residual solvent peak used as an internal reference. The mass spectra were measured by HRMS (MALDI-TOF, recorded on a Bruker Ultraflextreme mass spectrometer) and HRMS (ESI-TOF, recorded on an Agilent G6224A mass spectrometer). UV–vis absorption spectra were measured on a UV-2550 UV–vis spectrophotometer (Shimadzu Ltd., Japan). Fluorescence spectra were recorded with an FS5 spectrofluorometer （Edinburgh instruments, UK）. Luminescence lifetimes of compounds were recorded with an OB920 luminescence lifetime spectrometer (Edinburgh Instruments, U.K.). All these calculations were performed with Gaussian 09W [1]. Natural transition orbital analysis were performed by the Multiwfn program [2].

# 2. Synthesis of the compounds

**2.1. Synthesis of 2.** Compound **2** was synthesized in a manner similar to [3]. A mixture of **NI-3Br** (3.0 g, 7.750 mmol), bis(pinacolato)diboron (2.0 g, 7.750 mmol), KOAc (1.5 g, 15.5 mmol) and Pd(dppf)$Cl_2$ (257.4 mg, 0.320 mmol) in toluene (77mL) was degassed by gently bubbling $N_2$ for 30 min. The mixture was then stirred at 110 °C for 16 h. After cooling, the mixture was extracted with $CH_2Cl_2$ (100 mL × 3). The combined organic layer was washed with water (100 mL), brine solution (100 mL), dried over anhydrous $Na_2SO_4$, filtered, and evaporated to dryness. The crude product was purified by column chromatography (silica gel, DCM/PE 1:6, v/v) to give product **2** as white solid (400 mg, 11.9%). $^{1}H$ NMR (400 MHz $CDCl_3$) $\delta$: 0.86–0.94 (m, 6H), 1.30–1.37 (m, 8H), 1.40 (s, 12H), 1.92–1.96 (m, 1H), 4.08–4.19 (m, 2H), 7.75 (m, $J$ = 15.51 Hz, 1H), 8.23 (d, $J$ = 8.13 Hz, 1H), 8.62 (d, $J$ = 25.01 Hz, 1H), 8.67 (s, 1H), 8.99 (s, 1H). $^{13}C$ NMR (125 MHz, $CDCl_3$) $\delta$: 164.63, 164.46, 138.00, 136.49, 134.07, 132.22, 131.73, 130.20, 127.84, 126.86, 123.82,

122.79, 84.56,44.18, 37.96, 30.80, 28.77, 24.95, 24.13, 23.09, 10.58. HRMS (MALDI, *m/z*) calcd for $C_{38}H_{34}N_2O_2S$ $[M+H]^+$, 435.2581, found 436.2665.

**2.2. Synthesis of NI-Ph-Br.** Under a $N_2$ atmosphere, compound **2** (61.1 mg, 0.141 mmol) and 1-bromo-4-iodobenzene (75.0 mg, 0.265 mmol) were dissolved in mixed solvent of toluene (3 mL), ethanol (0.8 mL) and water (0.5 mL), followed by the addition of $K_2CO_3$ (70.0 mg, 0.506 mmol). After bubbling with $N_2$ for 20 min, $Pd(PPh_3)_4$ (30 mg, 0.026 mmol) was added, and the mixture was refluxed for 8 h. Then, the reaction mixture was cooled to room temperature and extracted with dichloromethane. The organic layer was dried over anhydrous $Na_2SO_4$ and concentrated under reduced pressure to attain the crude product. The residue was purified by column chromatography (silica gel, DCM/PE 1:2, v:v) to afford compound **NI-Ph-Br** as pale white solid (64 mg, 92.9%). $^1$H NMR (400 MHz, $CDCl_3$) *δ*: 0.88–0.96 (m, 6H), 1.31–1.41 (m, 8H), 1.94–1.97 (m, 1H), 4.10–4.20 (m, 2H), 7.66 (s, 4H), 7.79 (m, *J* = 15.25 Hz, 1H), 8.25 (d, *J* = 8.00 Hz, 1H), 8.35 (s, 1H), 8.60 (d, *J* = 7.25 Hz, 1H), 8.83 (s, 1H). $^{13}$C NMR (125 MHz, $CDCl_3$,) *δ*: 164.46, 138.85, 138.20, 133.88, 132.36, 132.12, 131.19, 130.93, 130.28, 128.98, 127.53, 123.46, 122.80, 44.25, 38.00, 30.80, 29.71, 28.75, 10.68, 8.83. HRMS (MALDI, *m/z*) calcd for $C_{26}H_{26}BrNO_2$ $[M+H]^+$, 463.1147, found 464.1220.

**2.3. Synthesis of 3.** Compound **2** (300.0 mg, 0.690 mmol) and 5-bromo-2-iodo-1,3-dimethylbenzene (300 mg, 0.965 mmol) were dissolved in a deaerated mixed solvent containing toluene (7 mL), ethanol (2 mL), and water (1 mL). After the addition of the catalysts $Pd(PPh_3)_4$ (79.6 mg, 0.069 mmol) and potassium carbonate (285.7 mg, 2.067 mmol), the mixture was stirred for 15 min at room temperature. Then, the reaction suspension was heated to 78 °C and this reaction temperature was kept for 1 h. Following that the reaction temperature was further increased to 110 °C and stirring continued for additional 9 h. Then, the reaction mixture was allowed to cool to room temperature, extracted with chloroform, washed with water and brine. The combined organic layer was dried by anhydrous $Na_2SO_4$ and concentrated under reduced pressure. The residue was purified by column chromatography (silica gel, DCM/PE 1:4, v:v). Compound **3** was obtained as pale white solid **3** (150 mg, 60.6%). $^1$H NMR (400 MHz, $CDCl_3$,) *δ*: 0.86–0.96 (m, 6H), 1.29–1.42 (m, 8H), 1.93–1.98 (m, 1H), 2.02

(s, 6H), 4.08–4.19 (m, 2H), 7.33 (s, 2H), 7.80 (m, *J* = 15.51 Hz, 1H), 7.96 (d, *J* = 1.50 Hz, 1H), 8.20 (d, *J* = 8.13 Hz, 1H), 8.38 (d, *J* = 1.50 Hz, 1H), 8.63 (d, *J* = 7.26 Hz, 1H). $^{13}C$ NMR (125 MHz, $CDCl_{3,}$) *δ*: 164.51, 138.88, 138.23, 133.89, 132.37, 131.20, 130.31, 128.99, 127.54, 127.44, 123.48, 122.85, 44.25, 38.01, 30.81, 28.75, 24.11, 23.09, 14.11, 10.68. HRMS (MALDI, *m/z*) calcd for $C_{28}H_{30}BrNO_2$ $[M+H]^+$, 491.1460, found 492.1533.

# 3. Molecular structure characterization data

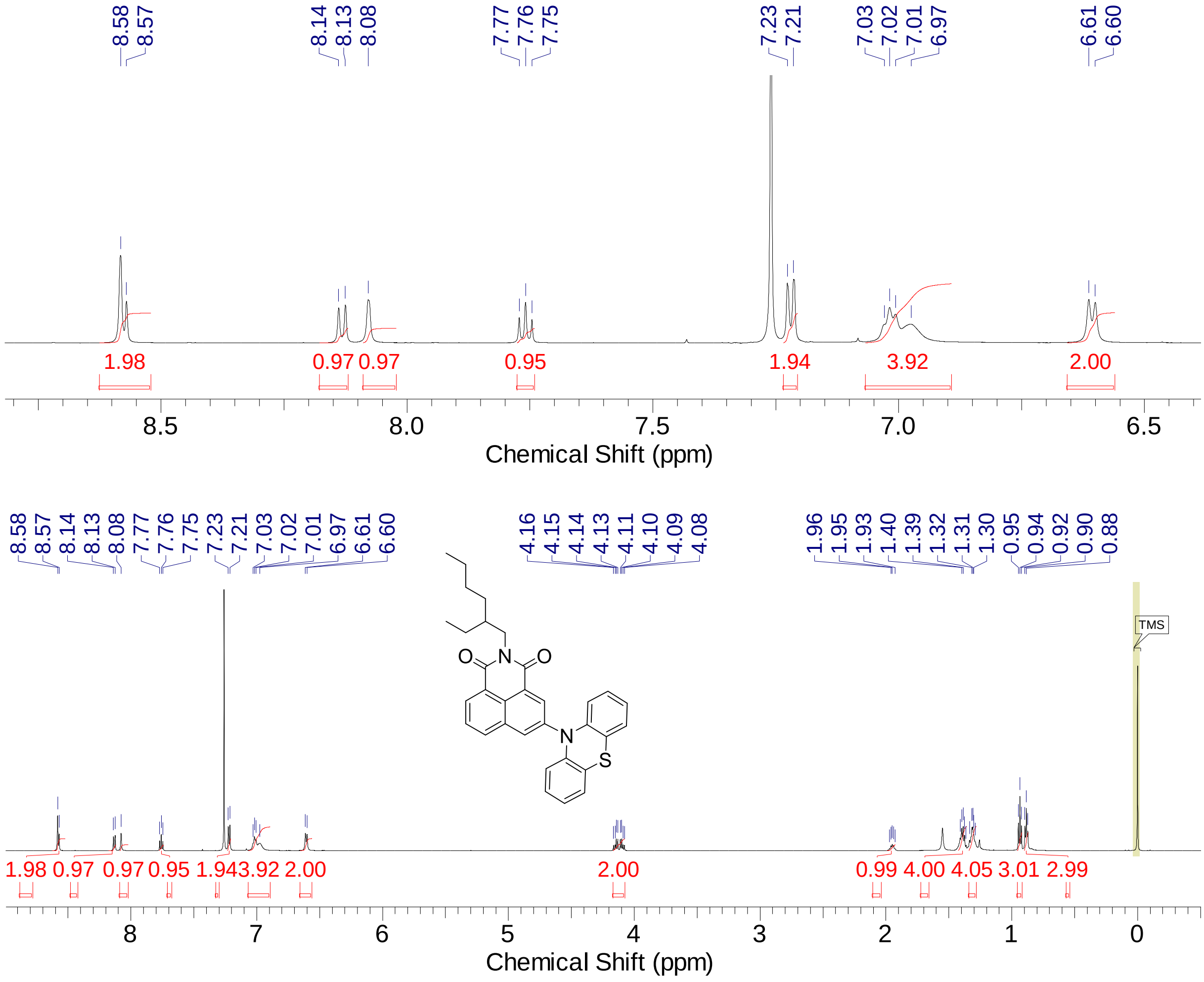


**Figure S1.** $^1$H NMR spectrum of **NI-PTZ** in $CDCl_3$ (400 MHz), 25 °C.

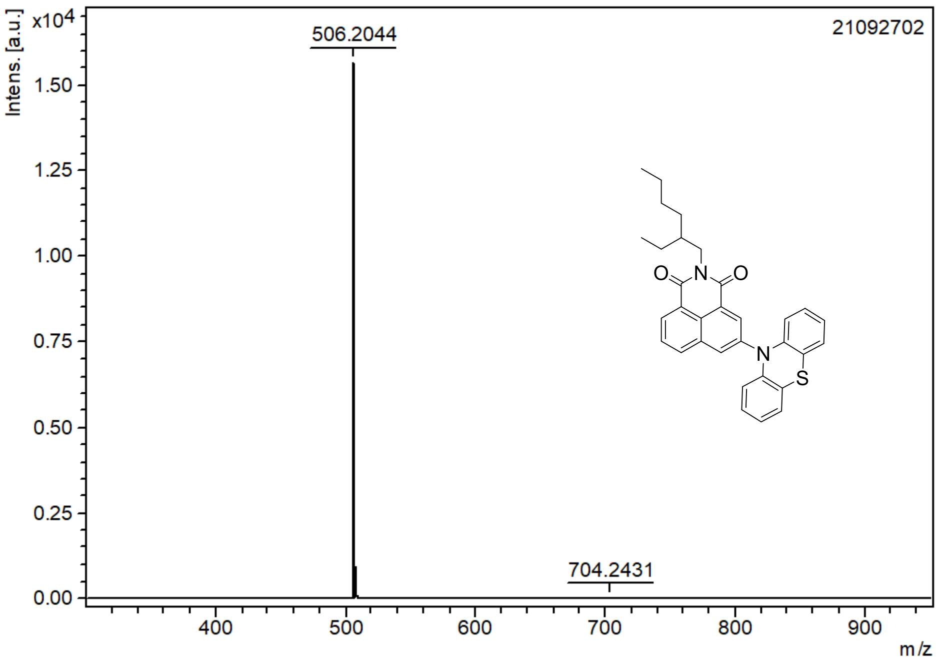


**Figure S2.** MALDI-HRMS spectrum of **NI-PTZ**, 25 °C.

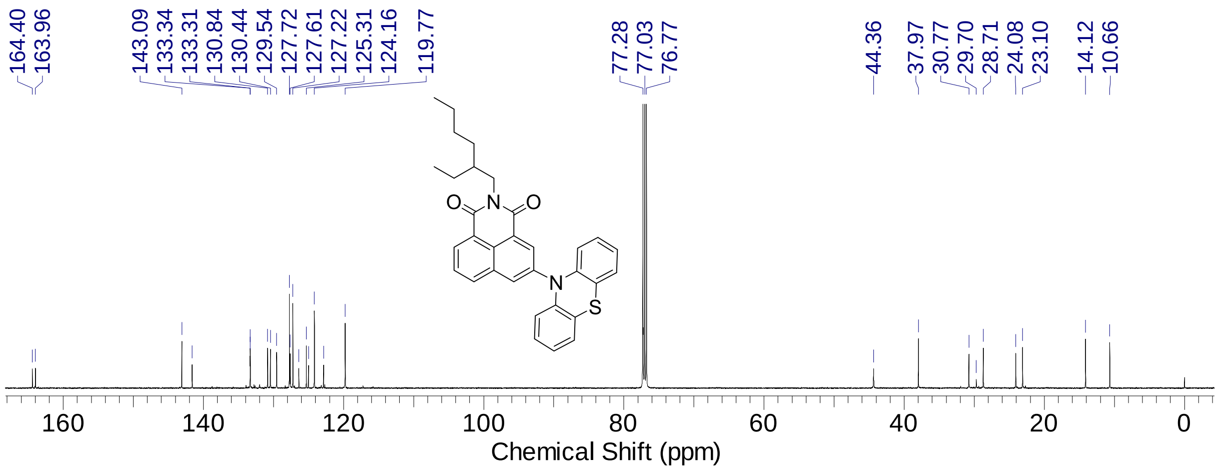


**Figure S3.** $^{13}$C NMR spectrum of **NI-PTZ** in $CDCl_3$ (125 MHz), 25 °C.

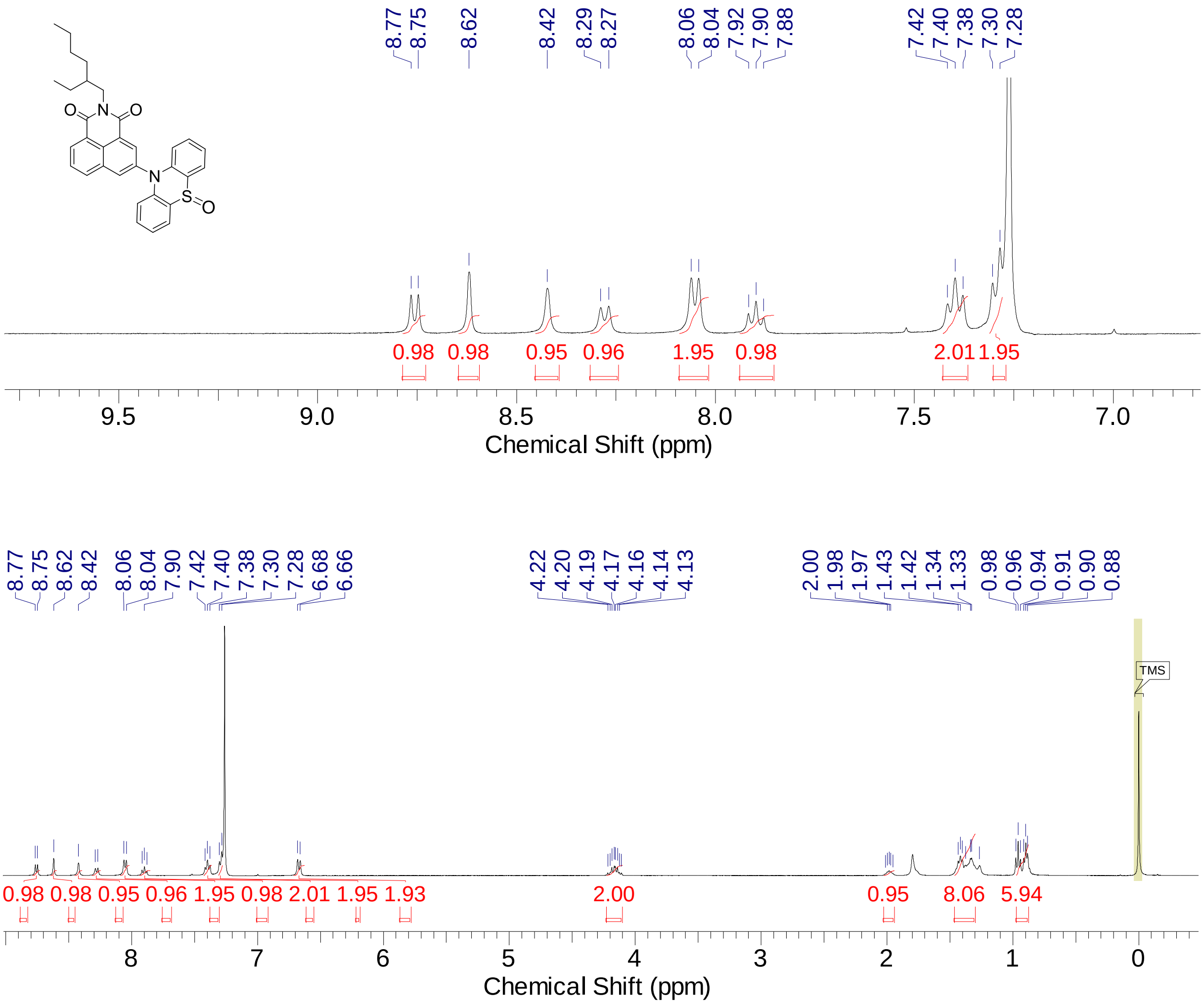


**Figure S4.** $^{1}$H NMR spectrum of **NI-PTZ-O** in $CDCl_3$ (400 MHz), 25 °C.

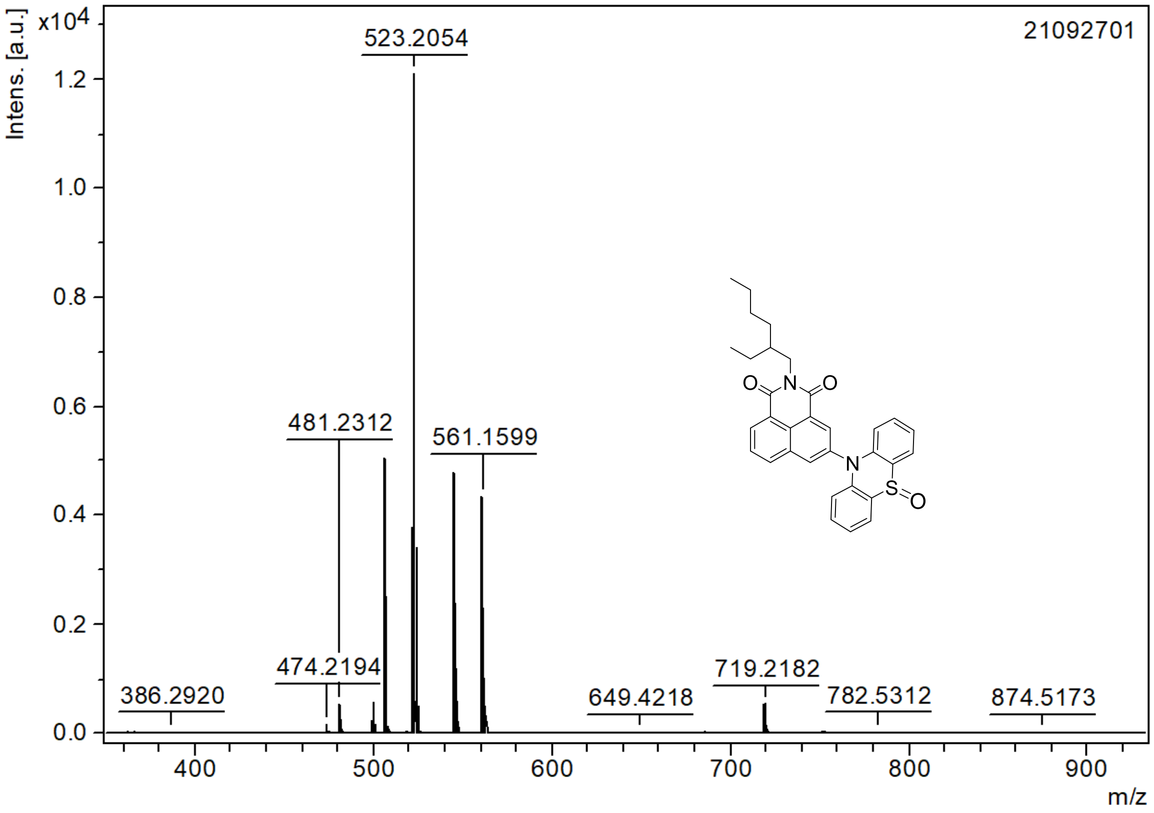


**Figure S5.** MALDI-HRMS spectrum of **NI-PTZ-O**, 25 °C.

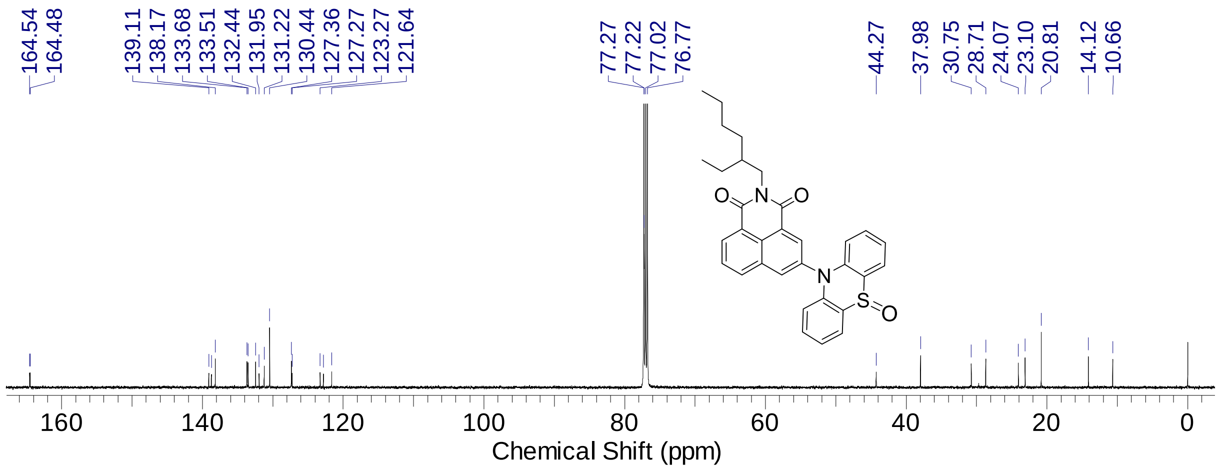


**Figure S6.** $^{13}C$ NMR spectrum of **NI-PTZ-O** in $CDCl_3$ (125 MHz), 25 °C.

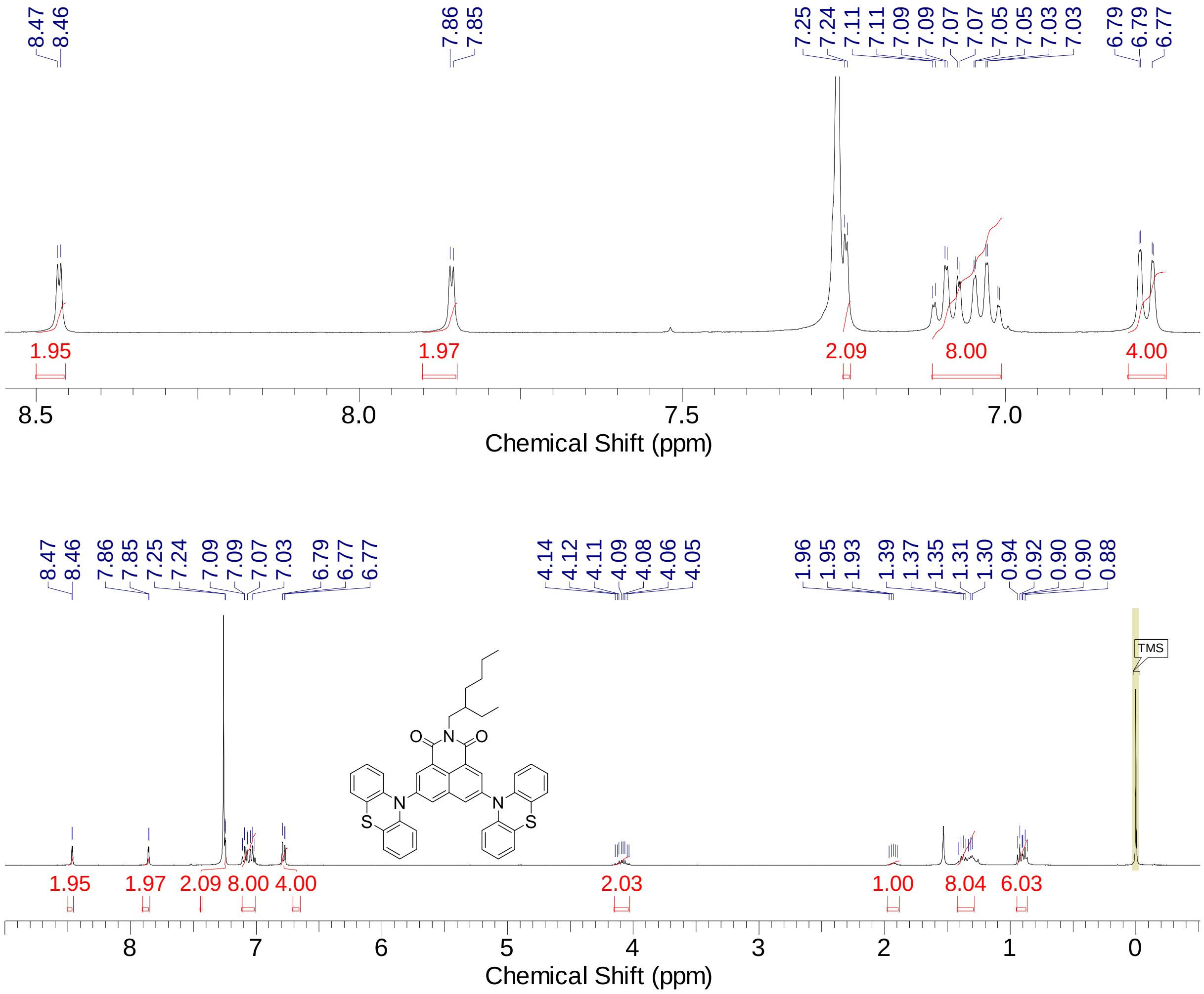


**Figure S7.** $^{1}H$ NMR spectrum of **NI-PTZ$_2$** in $CDCl_3$ (400 MHz), 25 °C.

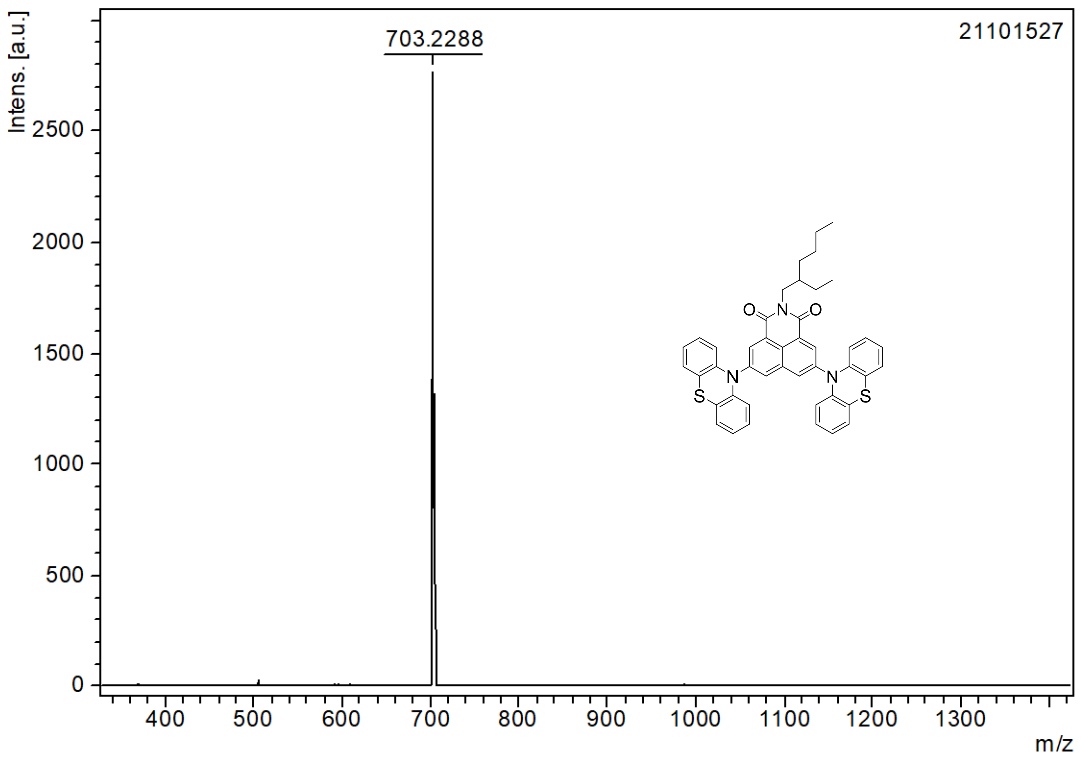


**Figure S8.** MALDI-HRMS spectrum of **NI-PTZ$_2$**, 25 °C.

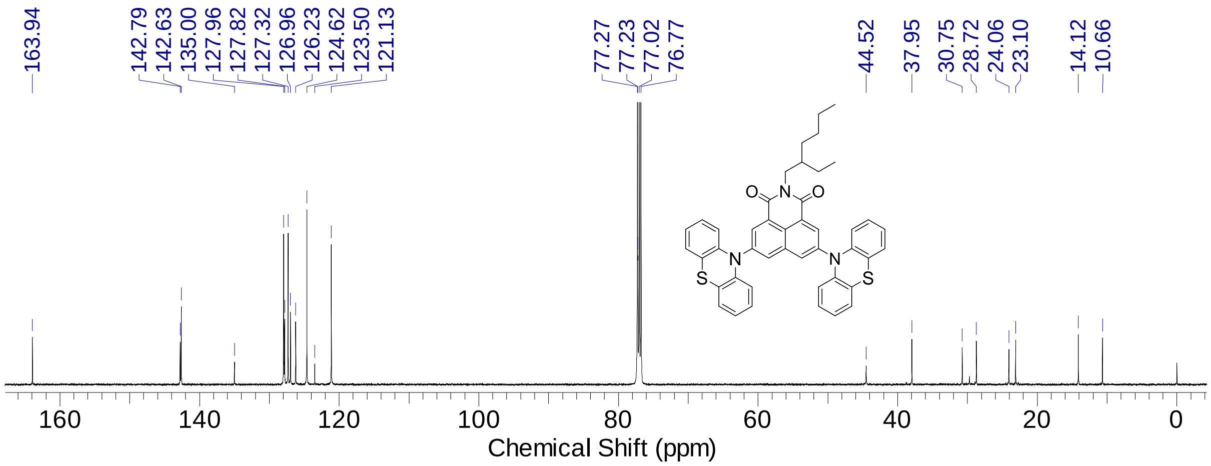


**Figure S9.** $^{13}$C NMR spectrum of **NI-PTZ$_2$** in $CDCl_3$ (125 MHz), 25 °C.

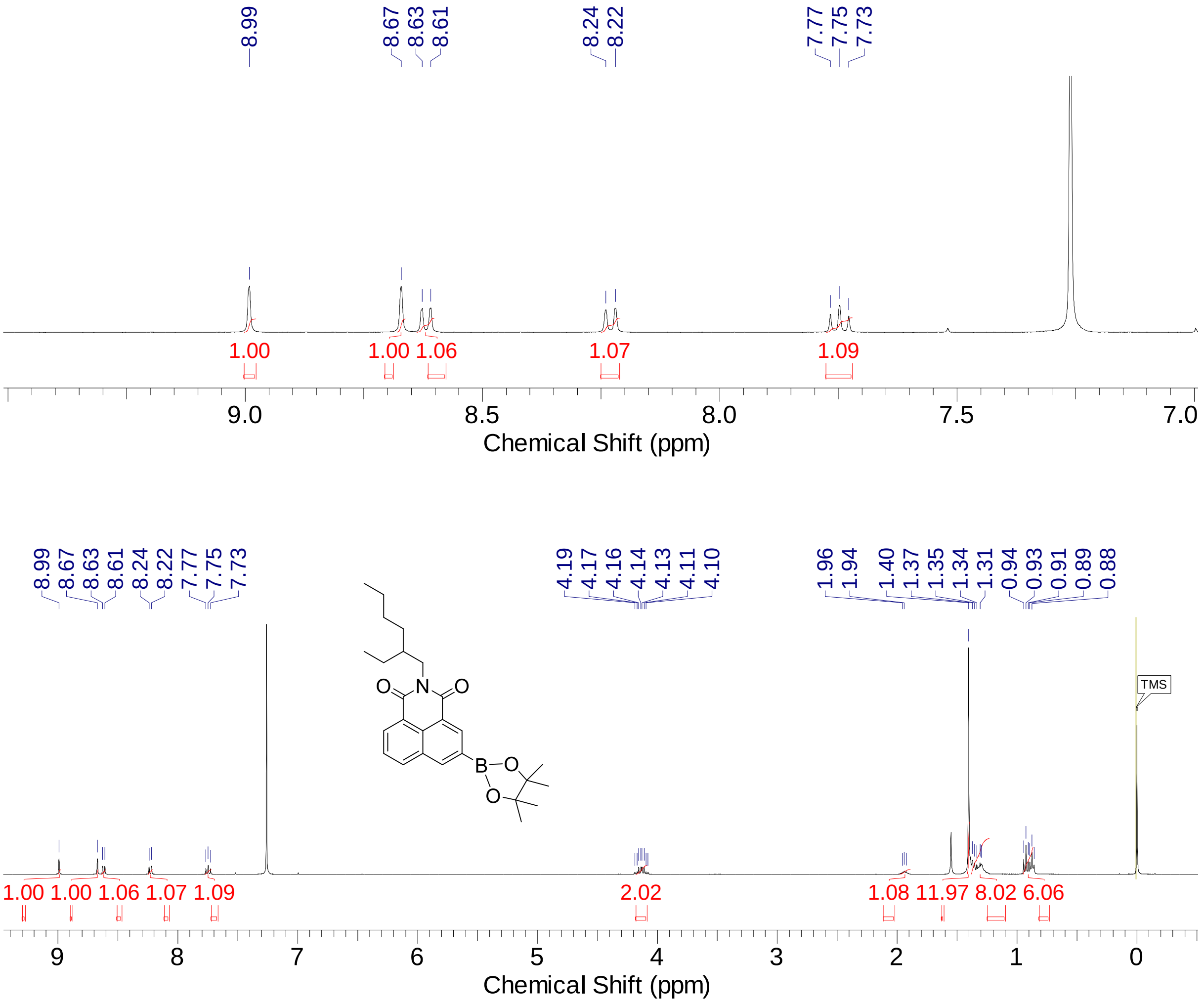


**Figure S10.** $^1$H NMR spectrum of **2** in $CDCl_3$ (400 MHz), 25 °C.

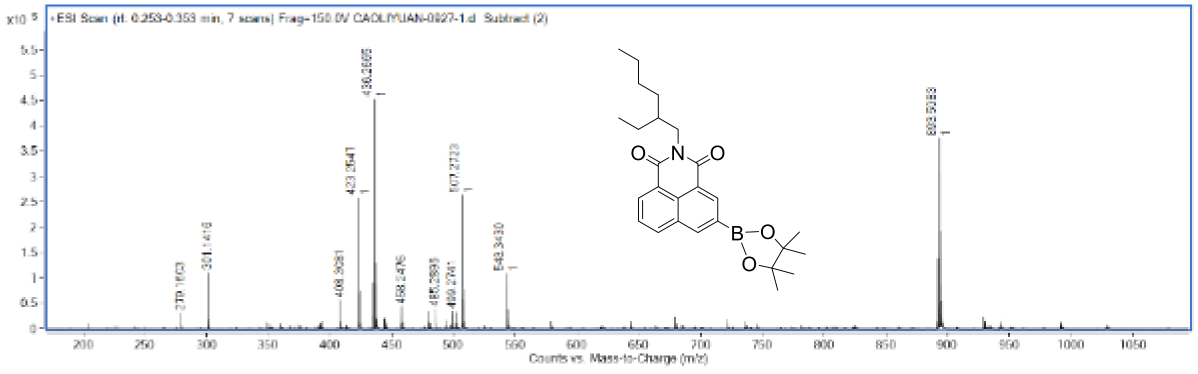


**Figure S11.** ESI-HRMS spectrum of **2**, 25 °C.

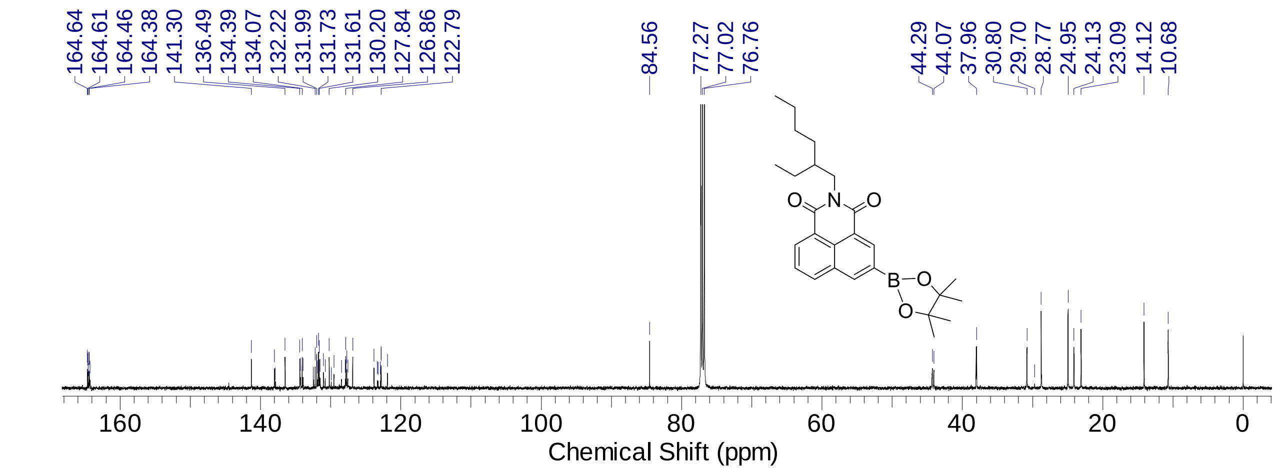


**Figure S12.** $^{13}$C NMR spectrum of **2** in $CDCl_3$ (125 MHz), 25 °C.

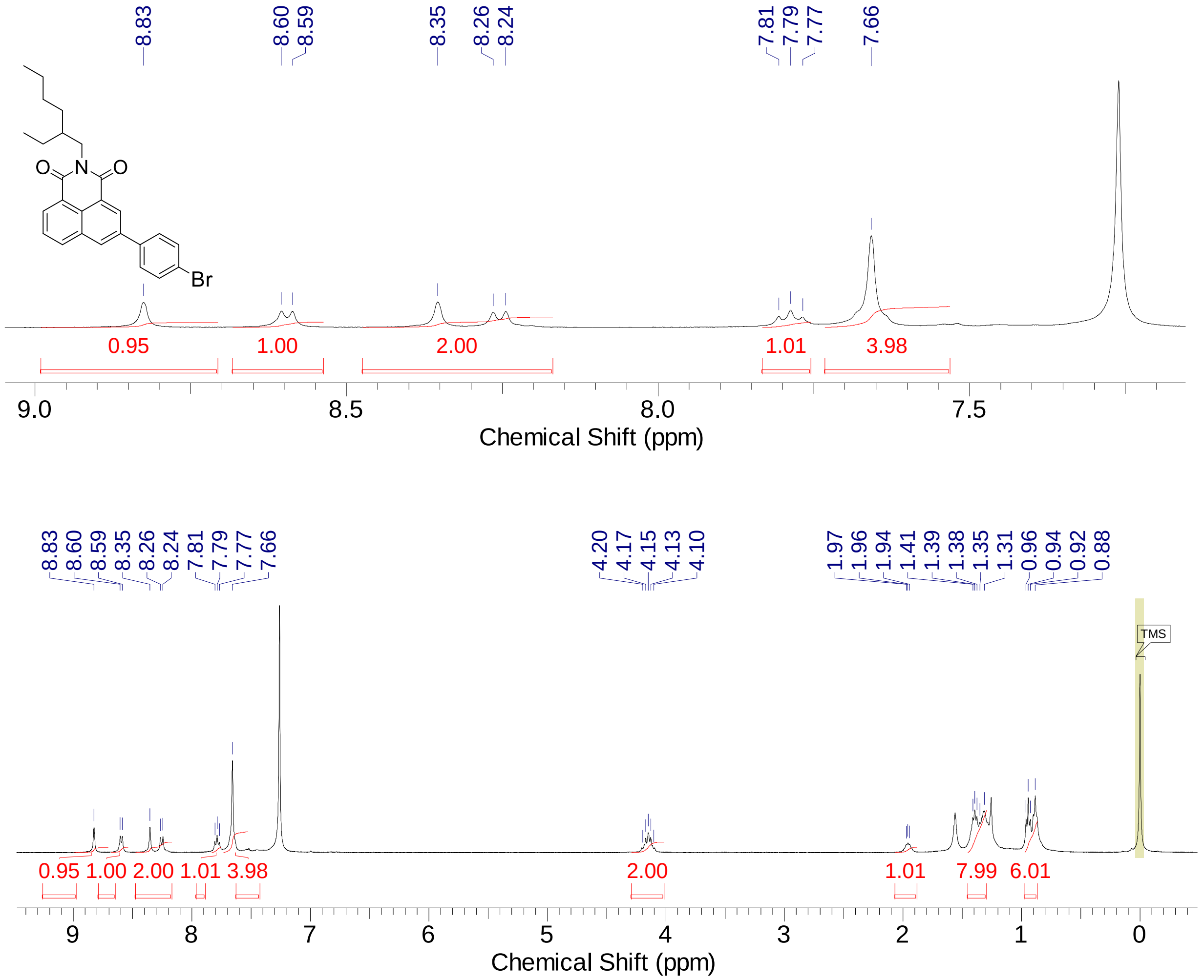


**Figure S13.** $^{1}$H NMR spectrum of **NI-Ph-Br** in $CDCl_3$ (400 MHz), 25 °C.

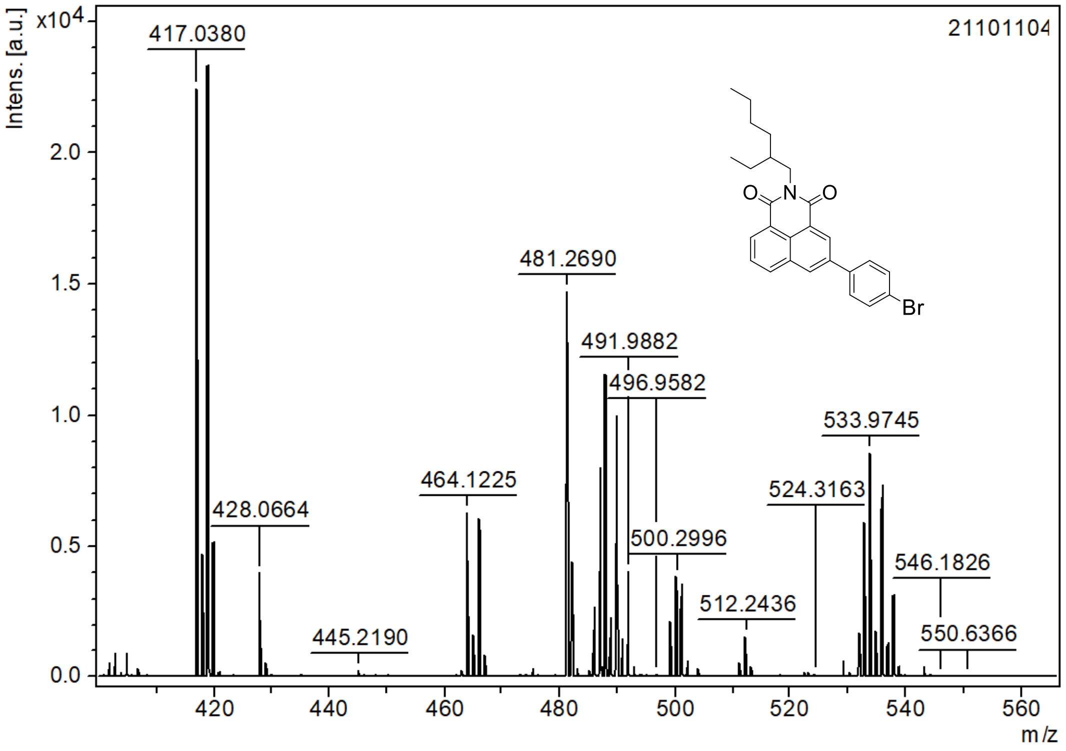


**Figure S14.** MALDI-HRMS spectrum of **NI-Ph-Br**, 25 °C.

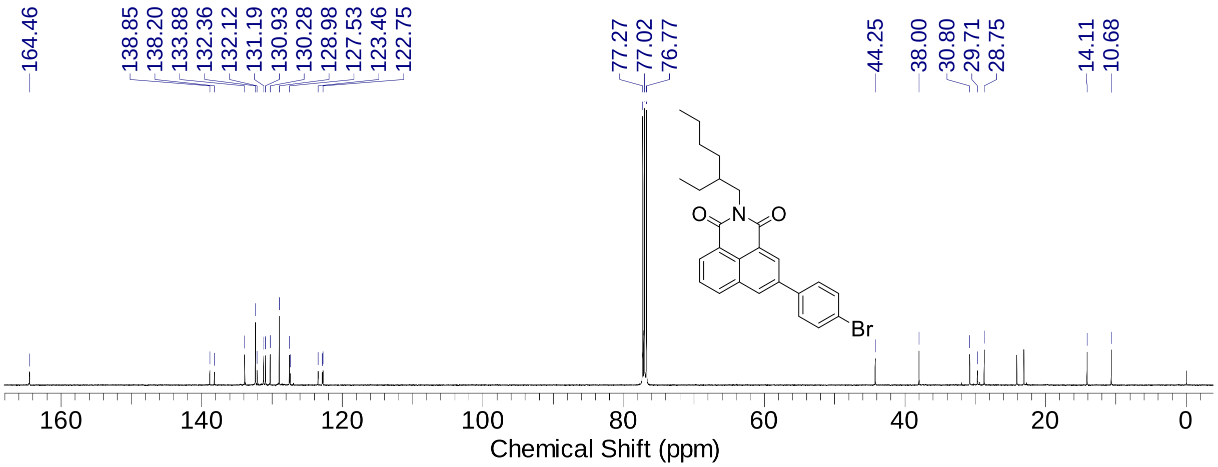


**Figure S15**. $^{13}C$ NMR spectrum of **NI-Ph-Br** in $CDCl_3$ (125 MHz), 25 °C.

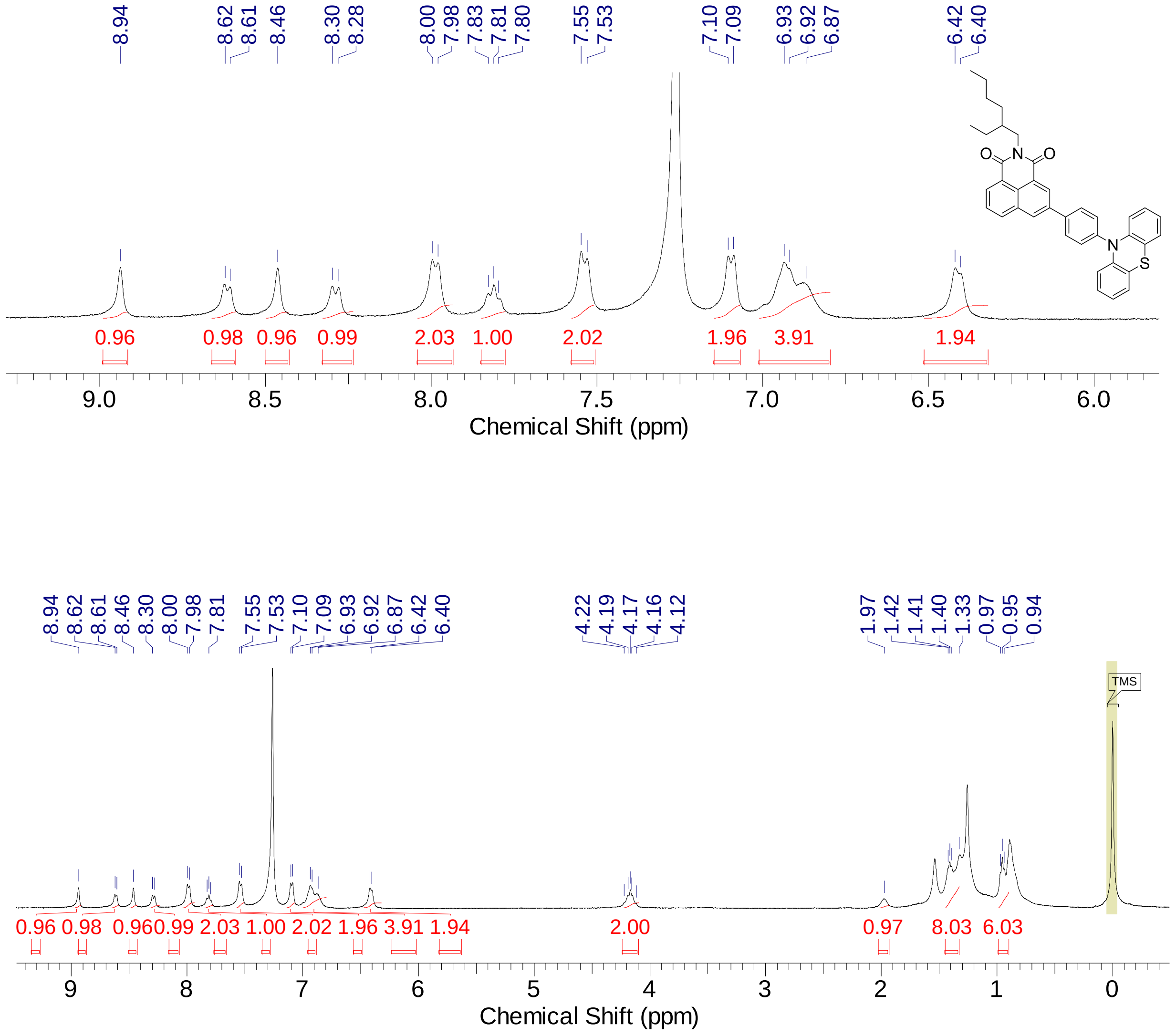


**Figure S16.** $^{1}H$ NMR spectrum of **NI-Ph-PTZ** in $CDCl_3$ (400 MHz), 25 °C.

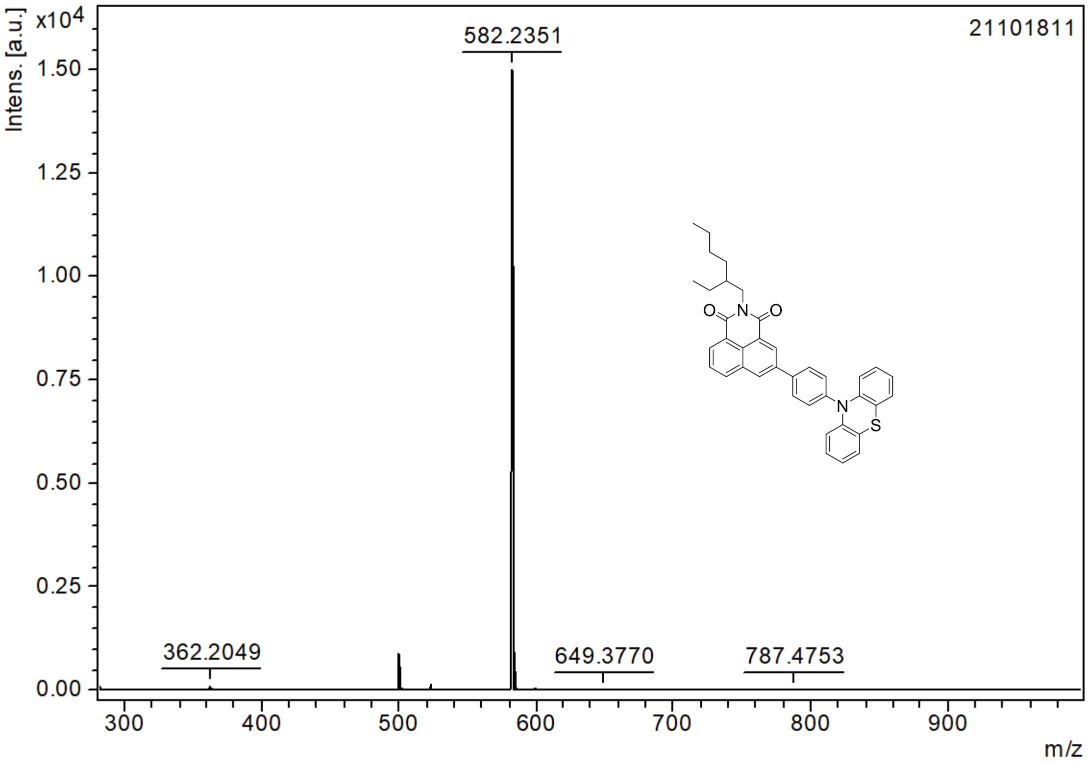


**Figure S17.** MALDI-HRMS spectrum of **NI-Ph-PTZ**, 25 °C.

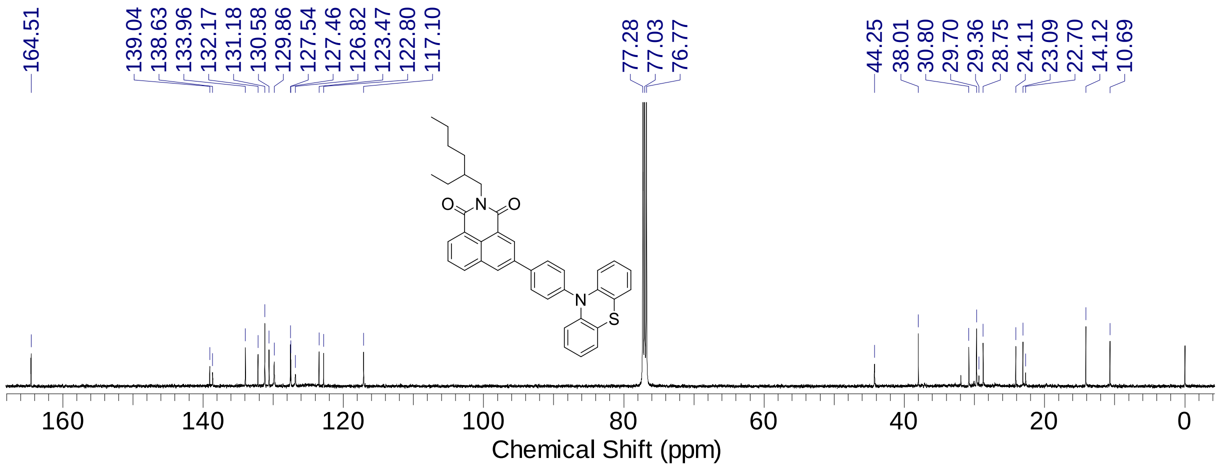


**Figure S18.** $^{13}$C NMR spectrum of **NI-Ph-PTZ** in $CDCl_3$ (125 MHz), 25 °C.

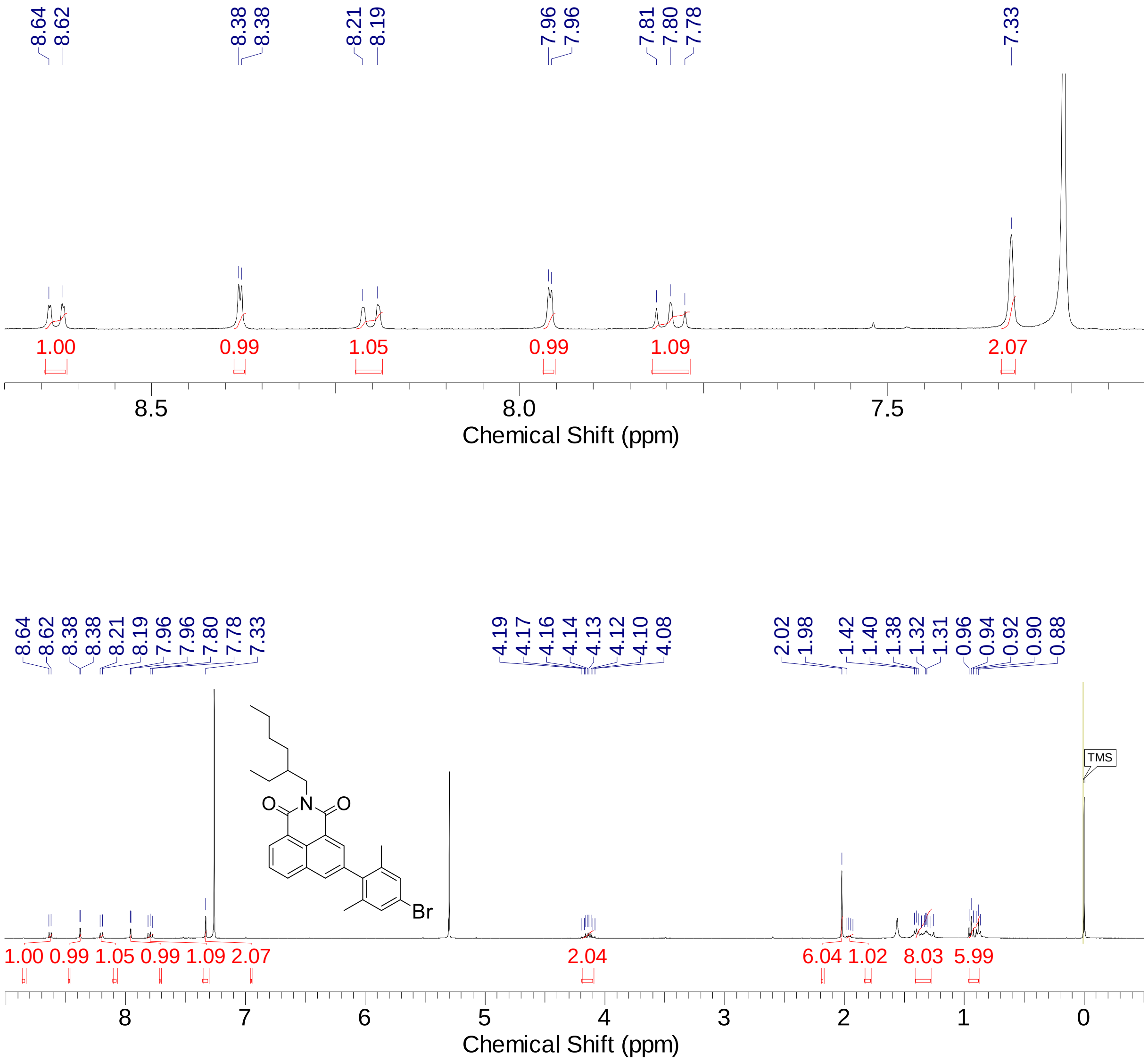


**Figure S19.** $^1$H NMR spectrum of **3** in $CDCl_3$ (400 MHz), 25 °C.

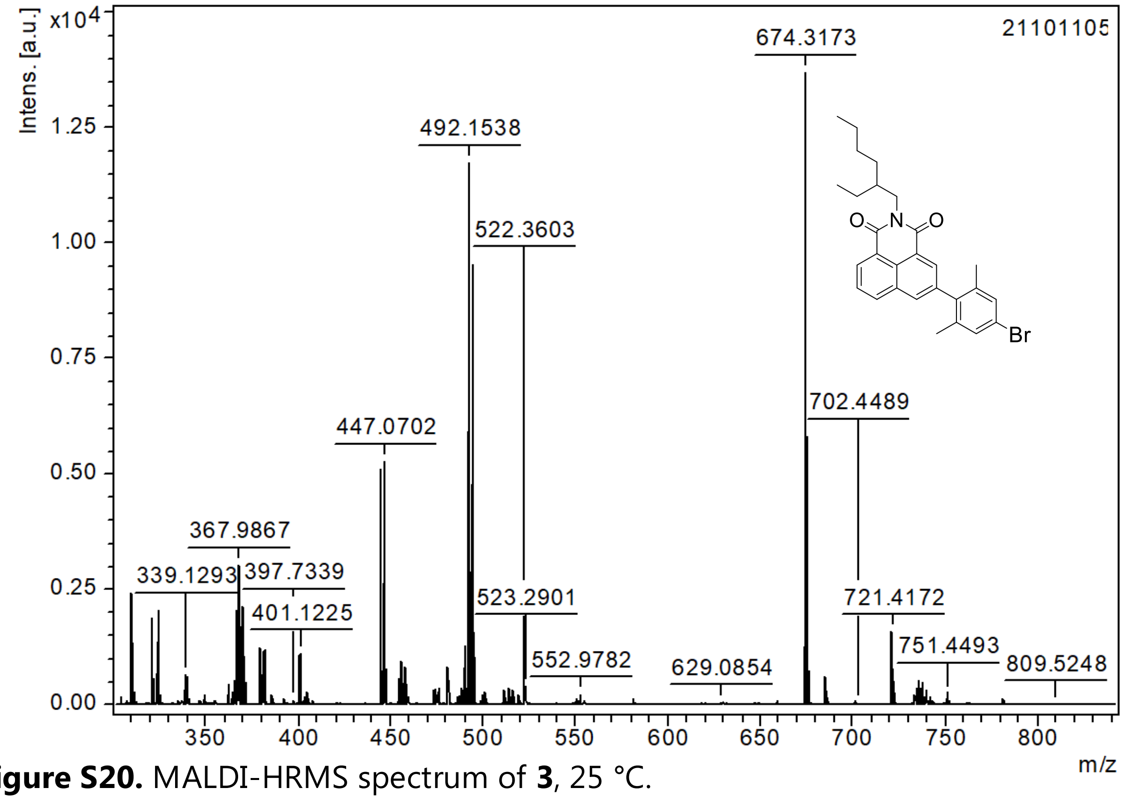


**Figure S20.** MALDI-HRMS spectrum of **3**, 25 °C.

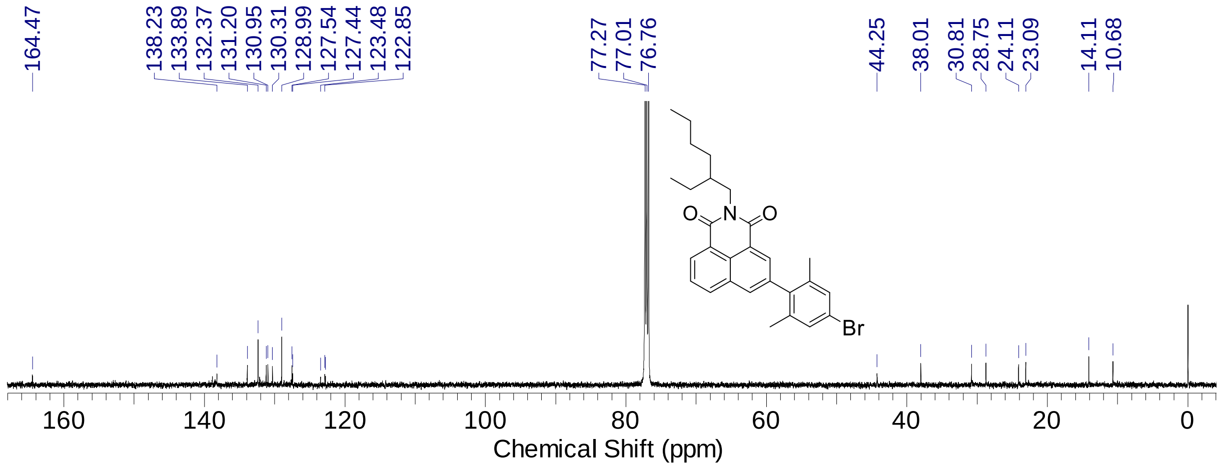


**Figure S21.** $^{13}C$ NMR spectrum of **3** in $CDCl_3$ (125 MHz), 25 °C.

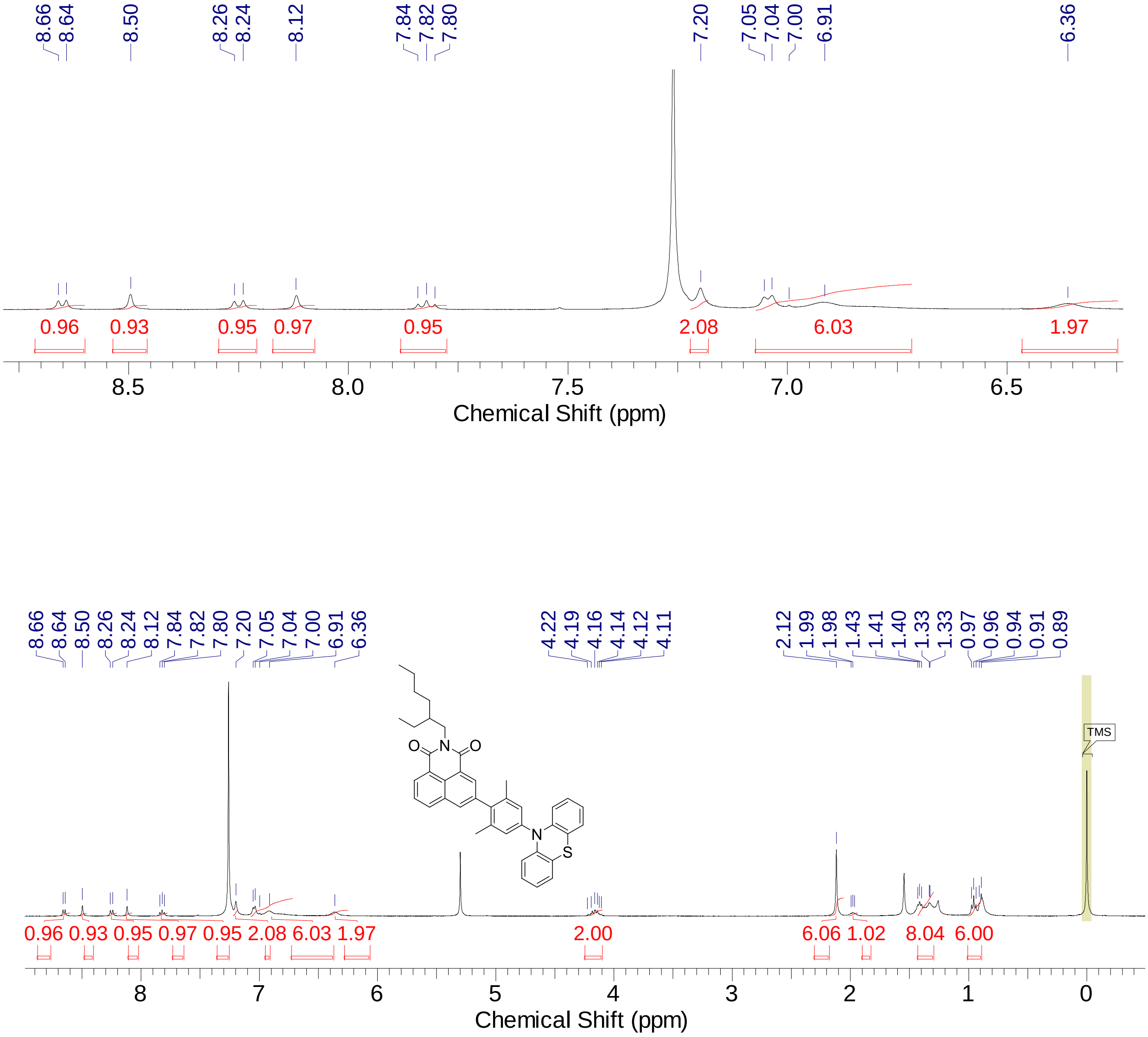


**Figure S22.** $^{1}H$ NMR spectrum of **NI-PhMe$_2$-PTZ** in $CDCl_3$ (400 MHz), 25 °C.

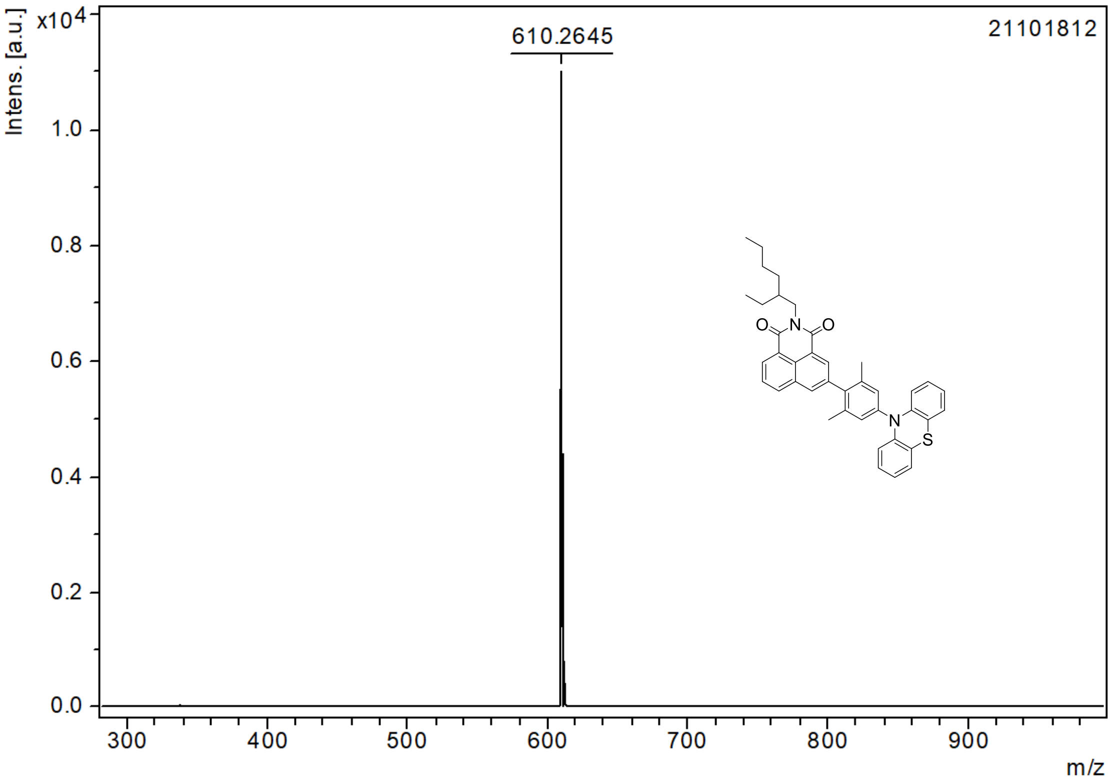


**Figure S23.** MALDI-HRMS spectrum of **NI-PhMe$_2$-PTZ**, 25 °C.

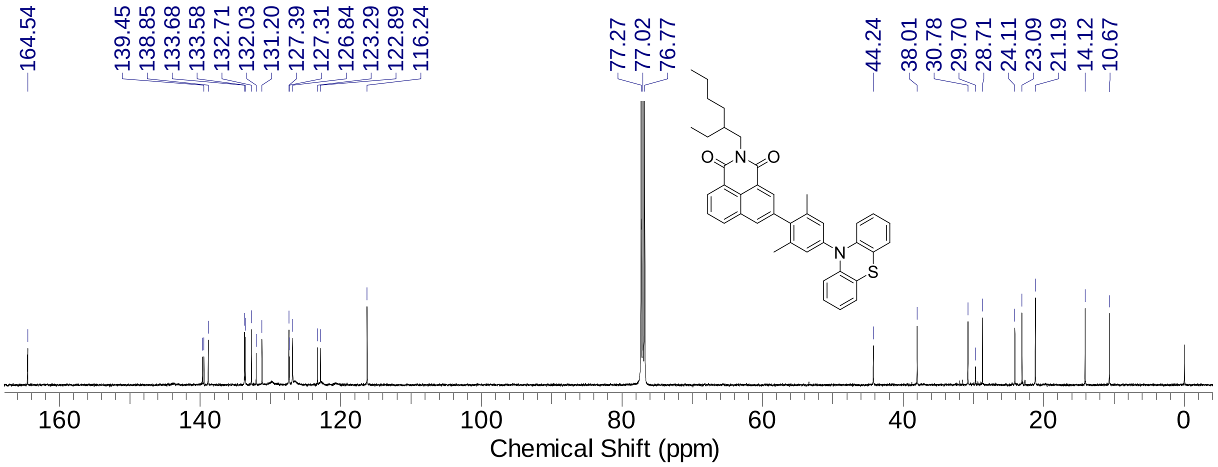


**Figure S24.** $^{13}$C NMR spectrum of **NI-PhMe$_2$-PTZ** in $CDCl_3$ (125 MHz), 25 °C.

## 4. Steady state UV–vis absorption and luminescence spectra

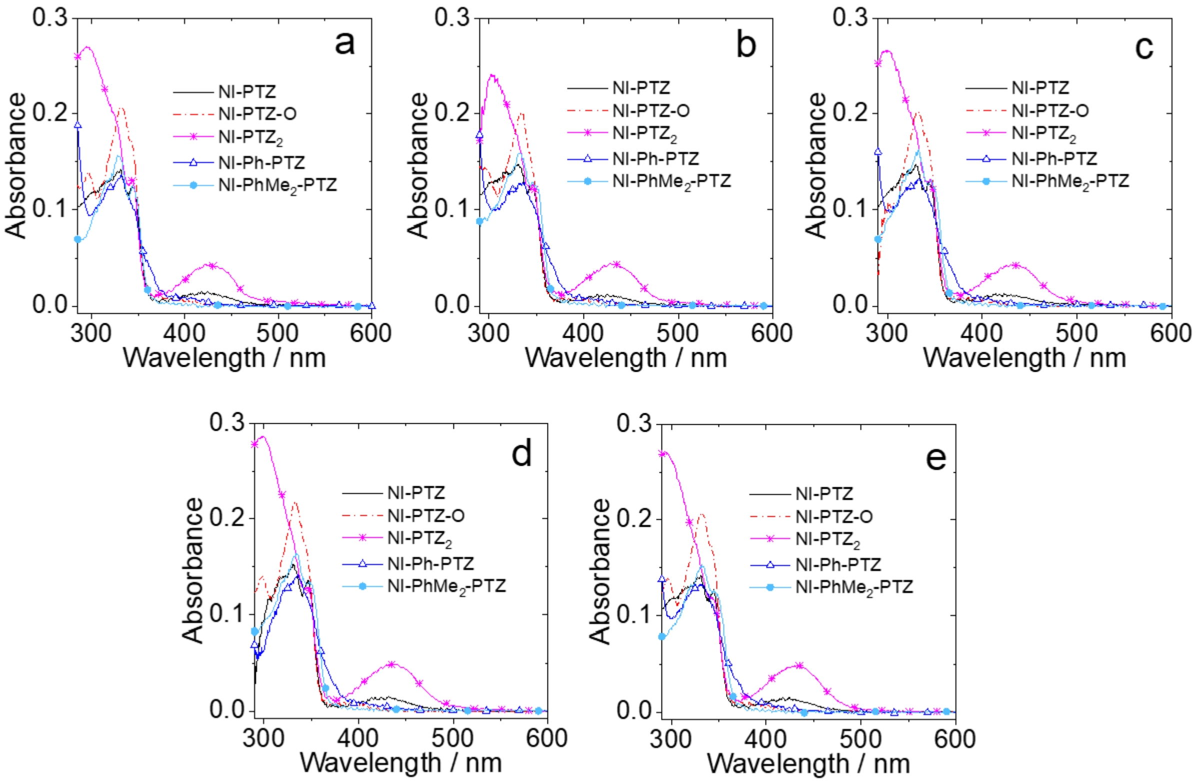


**Figure S25.** UV–vis absorption spectra of **NI-PTZ**, **NI-PTZ-O**, **NI-PTZ₂**, **NI-Ph-PTZ**, and **NI-PhMe₂-PTZ** in (a) cyclohexane (CHX); (b) toluene (TOL); (c) tetrahydrofuran (THF); (d) dichloromethane (DCM); (e) acetonitrile (ACN). $c = 1.0 \times 10^{-5}$ M, 20 °C.

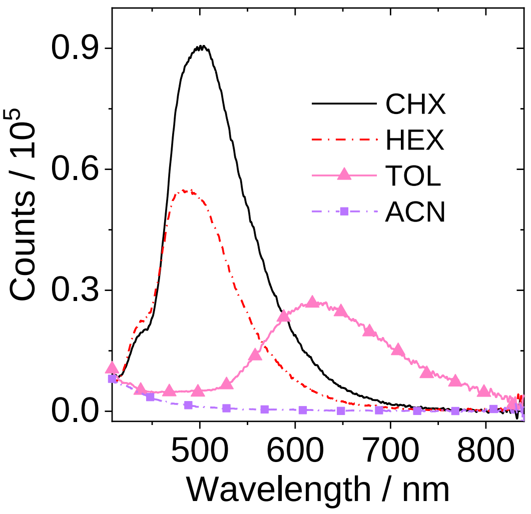


**Figure S26.** Fluorescence emission spectra of **NI-PhMe$_2$-PTZ** in different solvents. $A$ = 0.146, $\lambda_{ex}$ = 330 nm, 20 °C.

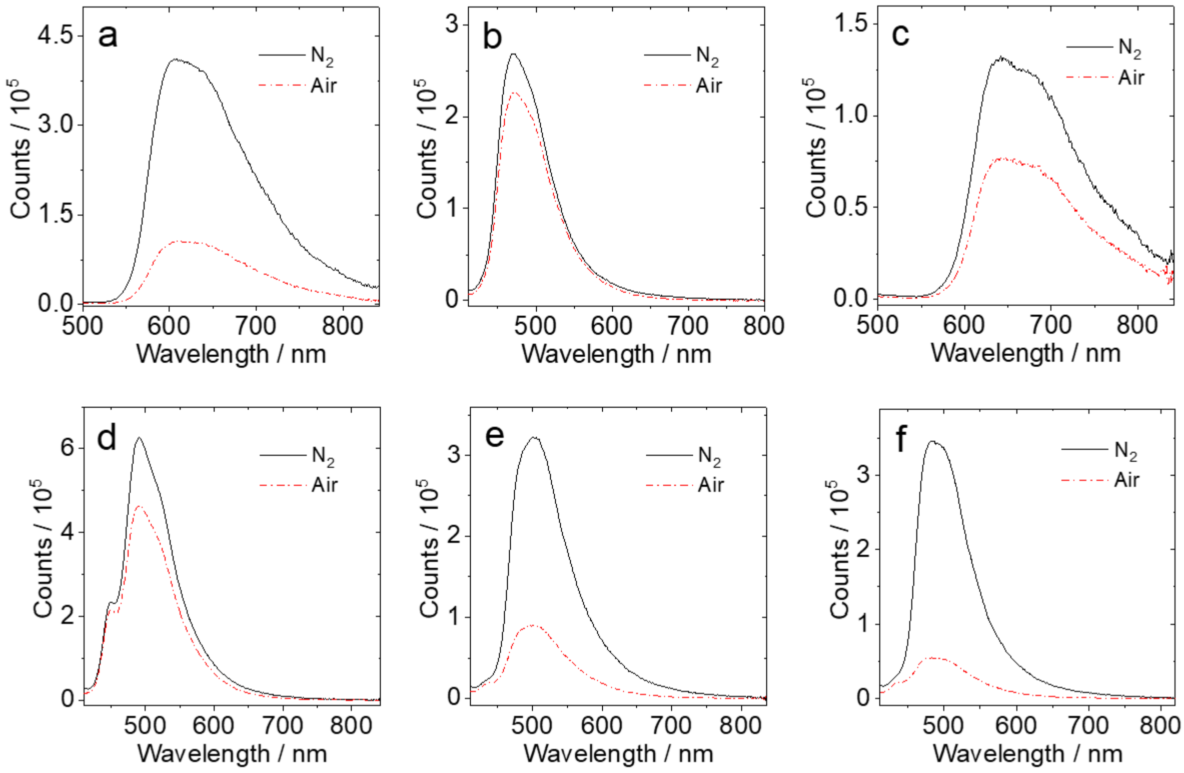


**Figure S27.** Fluorescence emission spectra of (a) **NI-PTZ**, (b) **NI-PTZ-O**, (c) **NI-PTZ$_2$**, (d) **NI-Ph-PTZ**, (e) **NI-PhMe$_2$-PTZ** in CYC, and (f) NI-PhMe$_2$-PTZ in *n*-HEX. Under different atmospheres ($N_2$, air). $A$ = 0.146, $\lambda_{ex}$ = 330 nm, 20 °C.

## 5. Fluorescence lifetimes

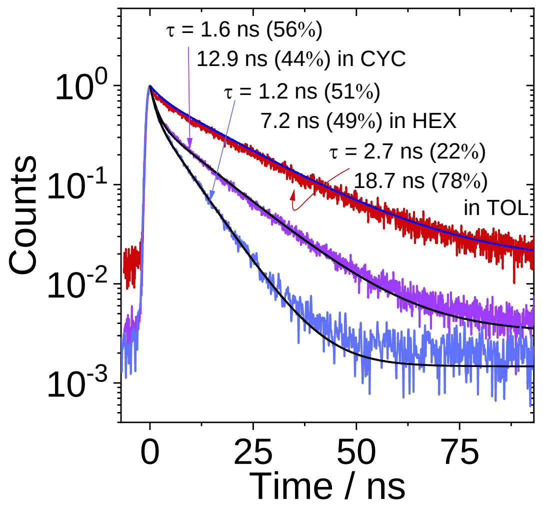


**Figure S28.** Decay traces of the luminescence of **NI-PhMe$_2$-PTZ** in different solvents. Excited with picoseconds pulsed laser for fluorescence band ($\lambda_{ex}$ = 479 nm), $c$ = 1.0 × $10^{-5}$ M, 20 °C.

## 6. Phosphorescence spectra

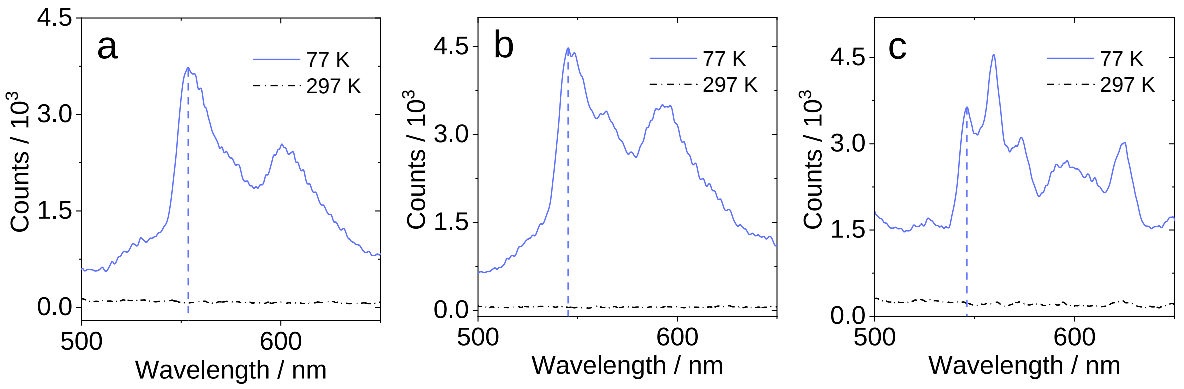


**Figure S29.** Phosphorescence spectra of the compounds (a) **NI-Ph-PTZ** ($\lambda_{ex}$ = 554 nm), (b) **NI-PhMe$_2$-PTZ** ($\lambda_{ex}$ = 545 nm), and (c) **NI-3Br** ($\lambda_{ex}$ = 547 nm) at 77 K, in 2-methyltetrahydrofuran, $c$ = 1.0 × $10^{-5}$ M.

## 7. Phosphorescence lifetimes

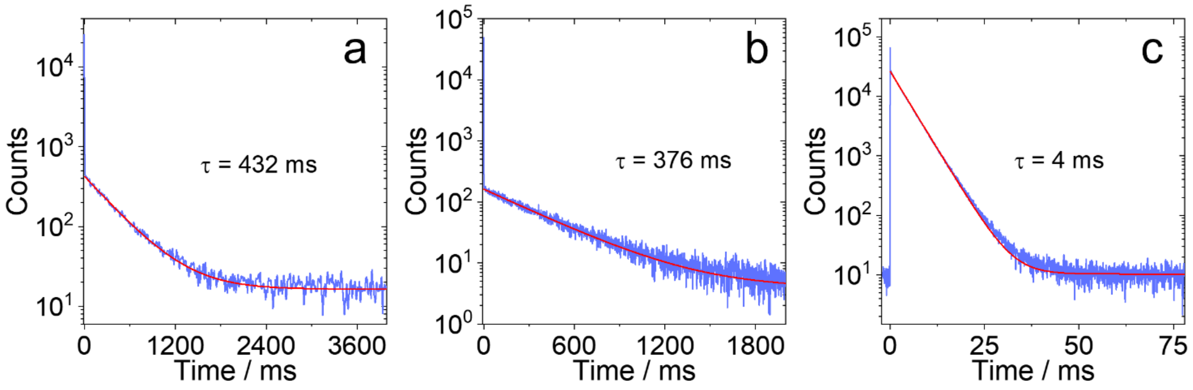


**Figure S30.** Decay traces of the phosphorescence of the compounds (a) **NI-Ph-PTZ** ($\lambda_{ex}$ = 554 nm), (b) N**I-PhMe$_2$-PTZ** ($\lambda_{ex}$ = 545 nm), and (c) **NI-3Br** ($\lambda_{ex}$ = 547 nm) in 2-methyltetrahydrofuran, $c$ = 1.0 × $10^{-5}$ M, 77 K.

## 8. Spectroelectrochemistry measurement

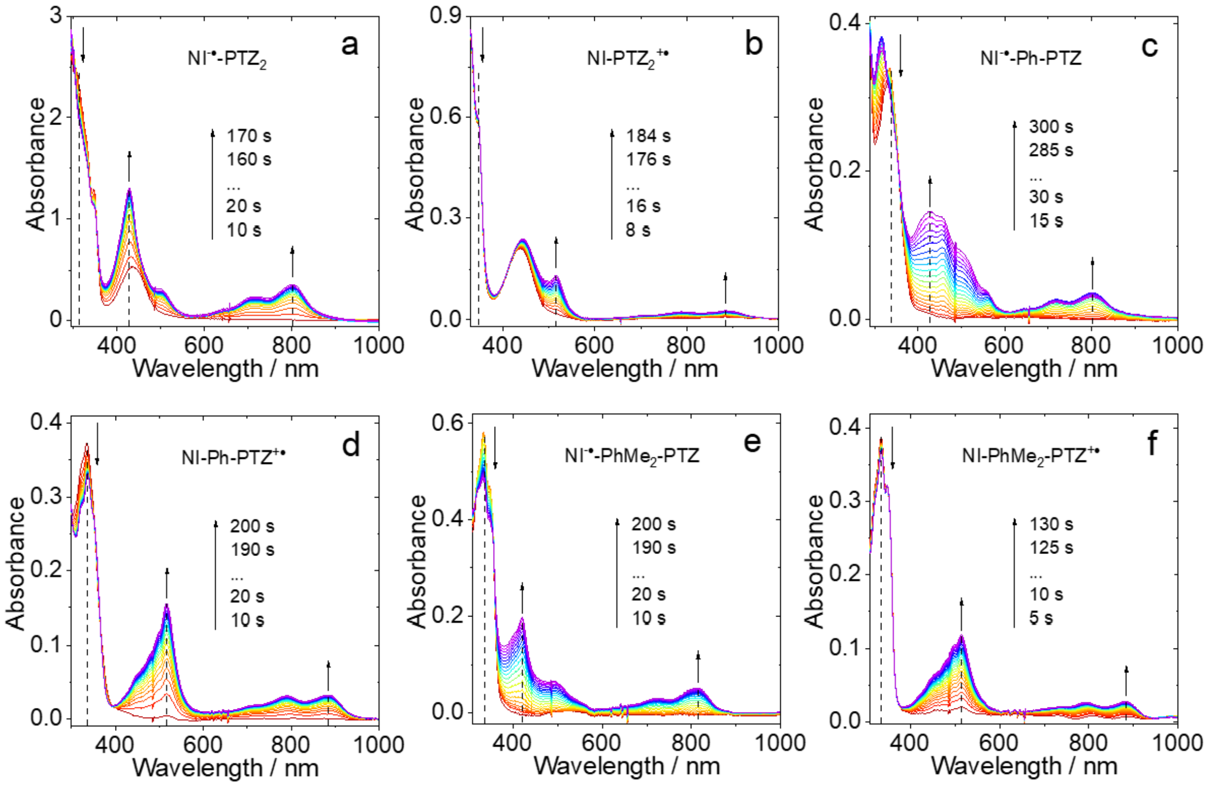


**Figure S31.** Spectroelectrochemistry traces of the UV–vis absorption spectra for (a) **NI-PTZ₂** observed from neutral (red) to monoanion (purple) at controlled potential of −1,83 V (vs. Ag/AgNO3), (b) **NI-PTZ2** observed from neutral (red) to monocationic (purple) at controlled-potential of 0.53 V (vs. Ag/AgNO3), (c) **NI-Ph-PTZ** observed from neutral (red) to monoanion (purple) at controlled potential of −1.85 V (vs. Ag/AgNO3), (d) **NI-Ph-PTZ** observed from neutral (red) to monocationic (purple) at controlled-potential of 0.55 V (vs. Ag/AgNO3), (e) **NI-PhMe2-PTZ** observed from neutral (red) to monoanion (purple) at controlled potential of −1,85 V (vs. Ag/AgNO3), (f) **NI-PhMe2-PTZ** observed from neutral (red) to monocationic (purple) at controlled potential of 0.60 V (vs. Ag/AgNO3). In deaerated dichloromethane containing 0.10 M Bu4[NPF6] as supporting electrolyte and with Ag/AgNO3 as reference electrode, 20 °C.

# 9. Nanosecond transient absorption spectroscopy

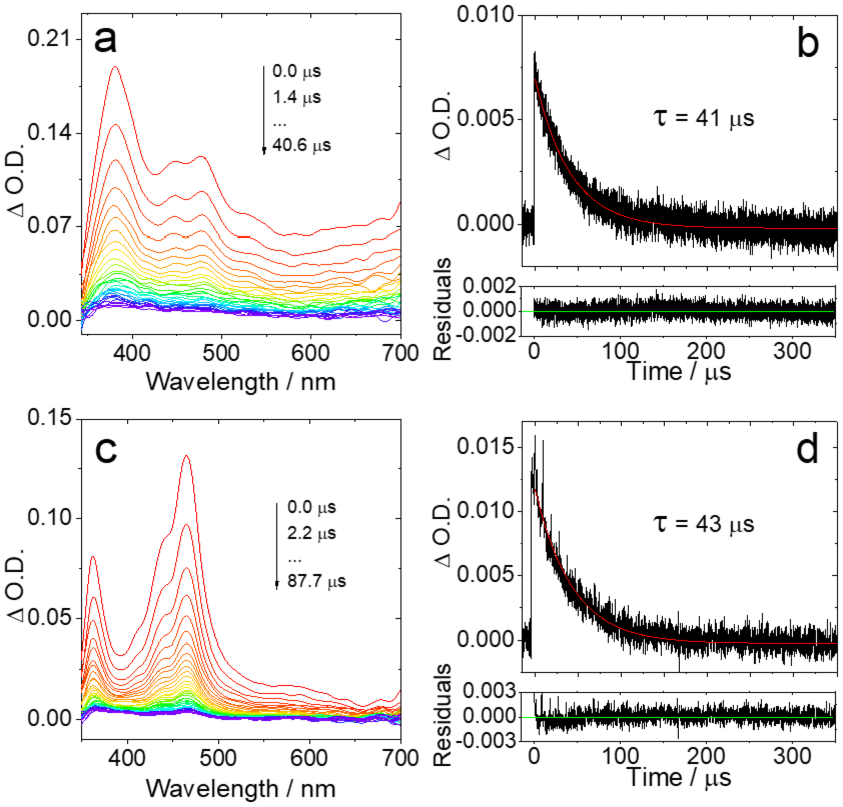


**Figure S32.** Nanosecond transient absorption of (a) **NI-Ph-PTZ** ($c$ = 5 × $10^{-5}$ M) and (c) **NI-PhMe$_2$-PTZ** ($c$ = 5 × $10^{-5}$ M). Decay traces of (b) **NI-Ph-PTZ** ($c$ = 2 × $10^{-6}$ M) at 380 nm and (d) **NI-PhMe$_2$-PTZ** ($c$ = 2 × $10^{-6}$ M). In deaerated HEX, $\lambda_{ex}$ = 355 nm, 20 °C.

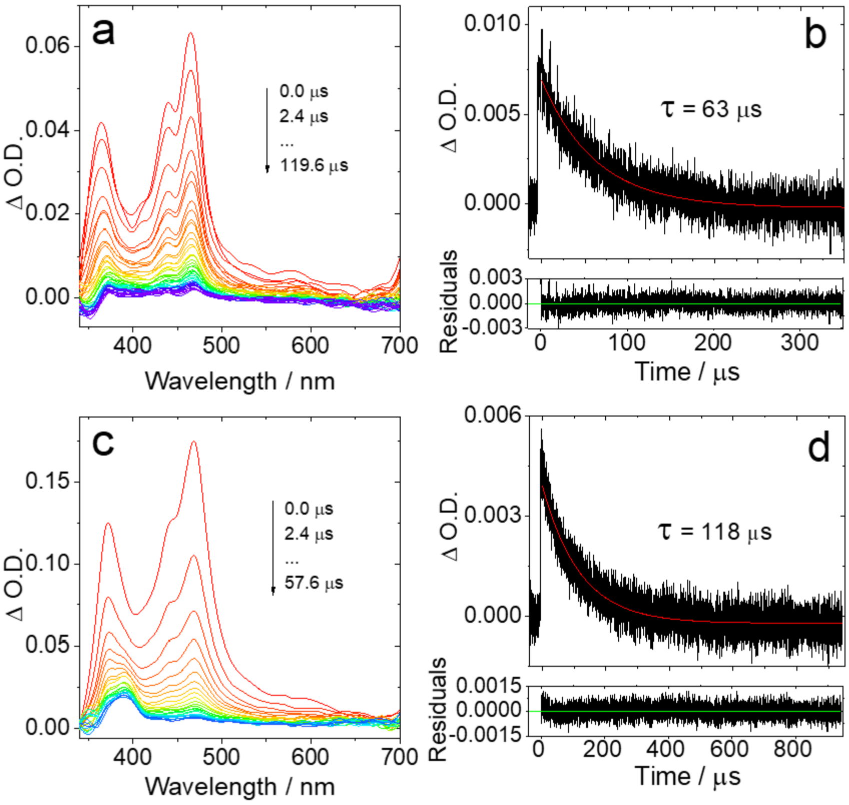


**Figure S33.** Nanosecond transient absorption of **NI-3Br** (a) $c$ = 3.0 × $10^{-5}$ M in deaerated HEX and (c) $c$ = 1.0 × $10^{-4}$ M in deaerated ACN. Decay traces of (b) $c$ = 2.0 × $10^{-6}$ M in deaerated HEX and (d) $c$ = 1.0 × $10^{-6}$ M in deaerated ACN at 470 nm. $\lambda_{ex}$ = 355 nm, 20 °C.

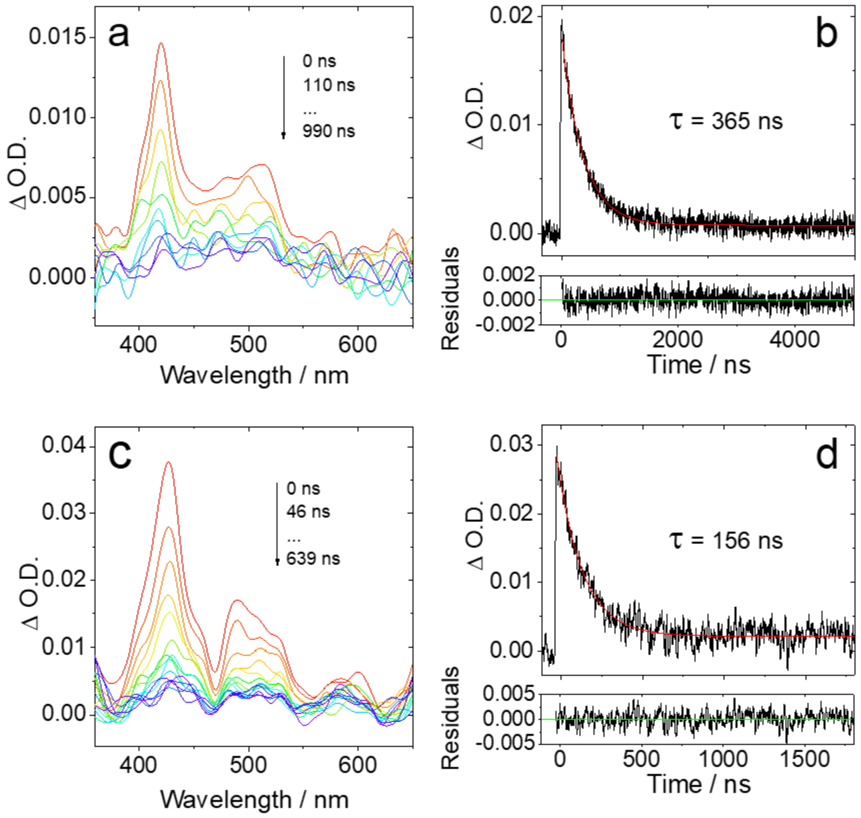


**Figure S34.** Nanosecond transient absorption of (a) **NI-PTZ** and (c) **NI-N-PTZ**. Decay trace of (b) **NI-PTZ** and (d) **NI-N-PTZ** at 420 nm. In deaerated ACN, $c = 1 \times 10^{-4}$ M, $\lambda_{ex}$ = 355 nm, 20 °C.

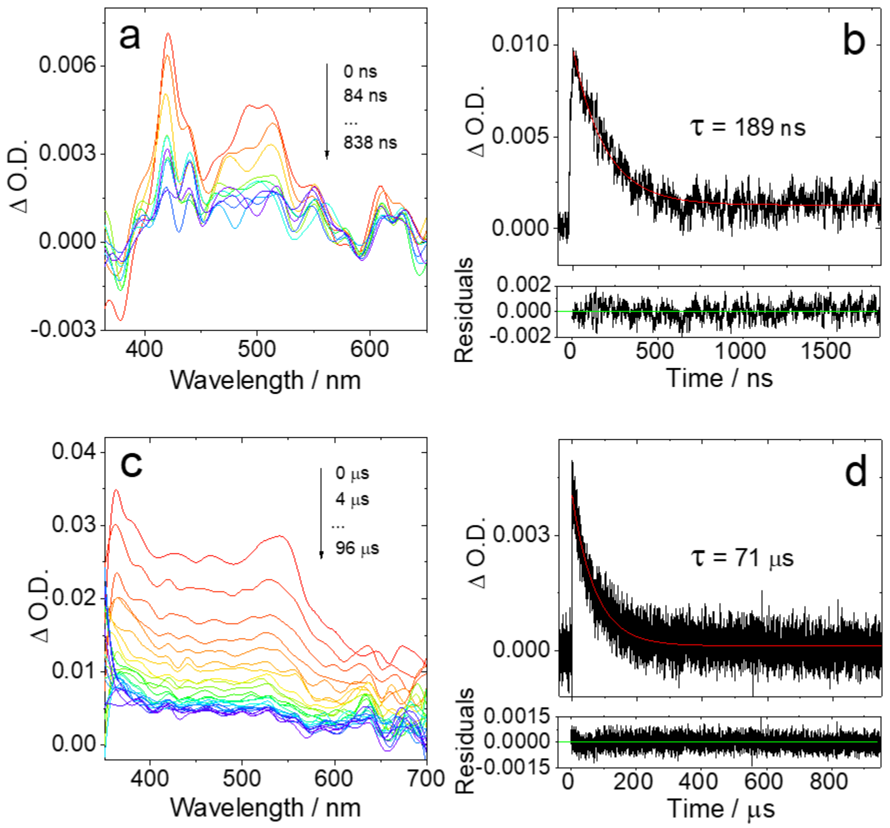


**Figure S35.** Nanosecond transient absorption of (a) **NI-PTZ$_2$** ($c$ = 1.0 × 10$^{-4}$ M) and (c) **NI-PTZ-O** ($c$ = 1.0 × 10$^{-5}$ M). Decay traces of (b) **NI-PTZ$_2$** ($c$ = 1.0 × 10$^{--4}$ M) at 420 nm and (d) **NI-PTZ-O** ($c$ = 1.0 × 10$^{-5}$ M) at 540 nm. In deaerated ACN, $\lambda_{ex}$ = 355 nm, 20 °C.

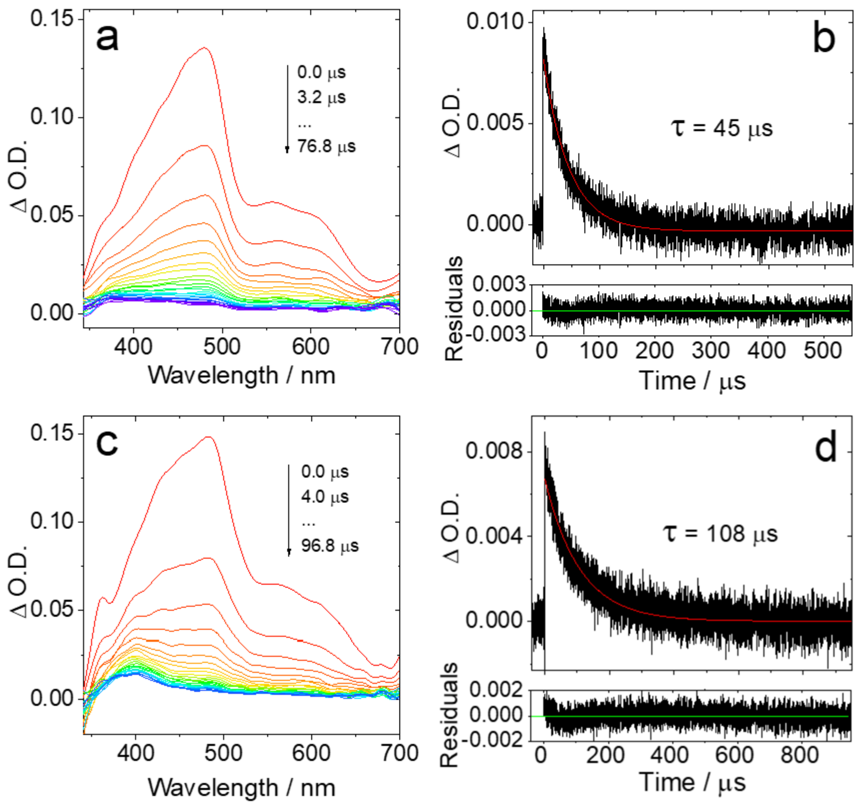


**Figure S36.** Nanosecond transient absorption of **NI-Ph-Br** (a) in HEX and (c) in ACN, $c = 5.0 \times 10^{-5}$ M. Decay traces of (b) in HEX and (d) in ACN, $c = 2.0 \times 10^{-6}$ M, $\lambda_{em}$ = 490 nm. $\lambda_{ex}$ = 355 nm, 20 °C.

## 10. Theoretical computation

**Table S1.** Vertical absorption of **NI-N-PTZ**, **NI-PTZ**, **NI-PTZ-O**, **NI-PTZ$_2$**, **NI-Ph-PTZ**, and **NI-PhMe$_2$-PTZ** (TDA-CAM-B3LYP/6-31G(d) in the gas phase, in eV) along with their oscillator strengths (*f*) and major orbital contributions. The energy levels and major orbital contributions for $T_1$ and $T_2$ at the Franck–Condon region are also given.

| Compounds | $S_1$ (absorption) | | | $T_1$ | | $T_2$ | |
|---|---|---|---|---|---|---|---|
| | $E_{abs}$ (eV) | *f* (a.u.) /$10^{-4}$ | C[a] MC[b,c] | $\Delta E_{vert}$ (eV)[d] | C[a] MC[b,c] | $\Delta E_{vert}$ (eV)[d] | C[a] MC[b,c] |
| **NI-N-PTZ** | 3.07 | 0 | CT H–L (0.67) | 2.69 | LE H-2–L (0.65) | 3.04 | CT H–L (0.66) |
| **NI-PTZ** | 3.17 | 1 | CT H–L (0.67) | 2.73 | LE H-2–L (0.67) | 3.12 | CT H–L (0.64) |
| **NI-PTZ-O** | 3.59 | 21 | CT H–L (0.68) | 2.74 | LE H-2–L (0.67) | 3.55 | CT H–L (0.66) |
| **NI-PTZ$_2$** | 3.06 | 1 | CT H–L (0.67) | 2.72 | LE H-4–L (0.64) | 3.00 | CT H–L (0.65) |
| **NI-Ph-PTZ** | 3.92 | 3 | CT H–L (0.61) | 2.67 | LE H-2–L (0.61) | 3.45 | LE H–L+4 (0.36) |
| **NI-PhMe$_2$-PTZ** | 3.90 | 3 | CT H–L (0.64) | 2.71 | LE H-2–L (0.66) | 3.65 | – H-4–L (0.56) |

[a] Character; [b] Major contributions; [c] H and L represent HOMO and LUMO, respectively. Predominant HOMO→LUMO transitions for $S_1$, $T_1$, and $T_2$, between brackets the corresponding coefficient; [d] $\Delta_{vert}$ corresponds to the energy difference between GS and $T_1$ and $T_2$ at the FC region.

**Table S2.** Emission energies (TDA-CAM-B3LYP/6-31G(d) in the gas phase, in eV) along with their oscillator strengths (*f*) and major orbital contributions. The energy levels and major orbital contributions for $S_1$, $T_1$ and $T_2$ are given at their respective optimal energy geometries.

| Compounds | $S_1$ (emission) | | | $T_1$ (emission)[e] | | $T_2$ (emission)[e] | |
|---|---|---|---|---|---|---|---|
| | $E_{ems}$ /eV | C[a] MC[b,c] | $f^d$ /$10^{-4}$ | $E_{ems}$ /eV | C[a] MC[b,c] | $E_{ems}$ /eV | C[a] MC[b,c] |
| **NI-N-PTZ** | 2.22 | CT H–L (0.69) | 0 | 1.93 | LE/CT H-1–L (c: 0.5)/H-L (0.4) | - | - |
| **NI-PTZ** | 2.21 | CT H–L (0.69) | 2 | 1.84 | CT H–L (0.64) | 2.01 | LE H(-2)–L (0.68) |
| **NI-PTZ-O** | 2.86 | CT H–L (0.68) | 1 | 2.02 | LE H(-2)–L (0.68) | 2.83 | CT H–L (0.67) |
| **NI-PTZ$_2$** | 2.09 | CT H–L (0.69) | 1 | 1.80 | LE/CT H-1–L (0.63) | - | - |
| **NI-Ph-PTZ** | 3.00 | CT H–L (0.65) | 0 | 2.01 | LE H(-1)–L (0.66) | - | - |
| **NI-PhMe$_2$-PTZ** | 3.24 | CT H–L (0.68) | 447 | 2.02 | LE H(-1)–L (0.60) | - | - |

[a]Character; [b]Major contributions; [c]H and L represent HOMO and LUMO, respectively. Predominant HOMO→LUMO transitions for $S_1$, $T_1$, and $T_2$, between brackets the corresponding coefficient; [d]oscillator strengths, eV; [e]$f = 0$.

**Table S3.** Calculated energy gaps at different regions of the photo deactivation decay (in eV) for **NI-PTZ** and **NI-PTZ-O**.

| Molecule | $\Delta E_{S, T\text{-}GS}$ (eV)[a] | | | $\Delta E_{S\text{-}T}$ (eV) | | $\Delta E_{T\text{-}GS}$ ($S_1$, eV)[b] | | $\Delta E_{S\text{-}T}$ ($S_1$, eV) | |
|---|---|---|---|---|---|---|---|---|---|
| | $^1$CT | $^3$LE | $^3$CT | $^1$CT–$^3$LE | $^1$CT–$^3$CT | $^3$LE | $^3$CT | $^1$CT–$^3$LE | $^1$CT–$^3$CT |
| **NI-PTZ** | 2.60 | 2.37 | 2.14 | 0.23 | 0.46 | 2.74 | 2.57 | -0.14 | 0.03 |
| **NI-PTZ-O** | 3.12 | 2.37 | 3.09 | 0.75 | 0.03 | 2.63 | 3.09 | 0.49 | 0.03 |

[a]$\Delta E_{S, T\text{-}GS}$ corresponds to the energy difference between the singlet (S) or triplet (T) minimum and the ground state (GS) minimum, i.e., adiabatic energy difference; [b]Relative energies at the $S_1$ optimized geometry, i.e., vertical energy difference.

**Vibronic models**

For the rate calculations, the Franck–Condon factors, $\langle \nu_i | \nu_f \rangle$, are computed by constructing the vibrational wavefunction as a sum of harmonic oscillators for both the initial and final state. Both these states are considered in the same reference frame, making it possible to connect them through translation and rotation of one of the states. This is accomplished by using the Duschinsky rotation effect (DRE), mapping the normal modes of one state onto the modes of the other state [4,5]. Here, a distinction can be made depending on how to approximate the harmonic normal modes from each state [6]. In vertical models, the same geometry is used for both initial and final state modes, while in adiabatic models the respective minima of both states are used for such a purpose. In addition, one could choose to calculate the gradient and/or hessian at only one or at both states. Representing a final state hessian by an initial state hessian will result in major errors, such that the adiabatic and vertical hessian (AH and VH, see exemplarily for ground and first excited state in Scheme S1) models, which

both use the Hessians from both the initial and the final states, are the only ones suitable for accurate calculations [6,7].

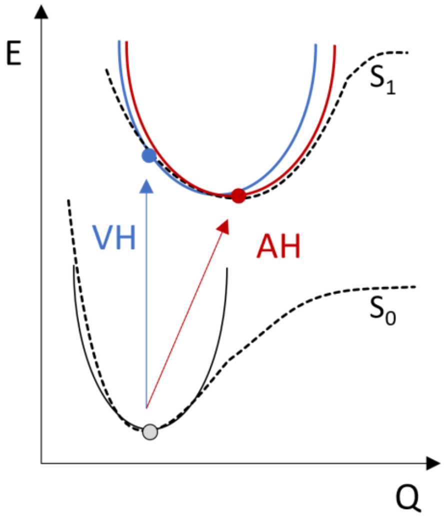


Scheme S1. Visualization of the adiabatic (AH) and vertical hessian (VH) models.

**Activation energies from the computed adiabatic energy difference gaps and reorganization energies**

According to [8], the activation energy for the RISC process ($\Delta G$) can be obtained with

$$\Delta \boldsymbol{G} = \frac{(\Delta \boldsymbol{E}_{if} - \boldsymbol{\lambda})^2}{4\boldsymbol{\lambda}}$$

where for a RISC $\Delta E_{\text{if}}$ corresponds to -$\Delta E_{\text{S-T}}$ (see values in Table S3) and $\lambda$ corresponds to the reorganization energies between the initial and final states.

## References

1) Gaussian 16, Gaussian, Inc: Wallingford, CT, 2016.
2) Multiwfn – A multifunctional Wavefunction analyzer; http://sobereva.com/multiwfn/
3) Boonnab, S.; Chaiwai, C.; Nalaoh, P.; Manyum, T.; Namuangruk, S.; Chitpakdee, C.; Sudyoadsuk, T.; Promarak, V. *Eur. J. Org. Chem.* **2021**, *2021*, 2402. doi: org/10.1002/ejoc.202100134.

4) Niu, Y.; Peng, Q.; Deng, C.; Gao, X.; Shuai, Z. Theory of Excited State Decays and Optical Spectra: Application to Polyatomic Molecules. *J. Phys. Chem. A* **2010**, *114* (30), 7817–7831. https://doi.org/10.1021/jp101568f.
5) Shuai, Z.; Peng, Q. Excited States Structure and Processes: Understanding Organic Light-Emitting Diodes at the Molecular Level. *Phys. Rep.* **2014**, *537* (4), 123–156. https://doi.org/10.1016/j.physrep.2013.12.002.
6) Humeniuk, A.; Bužančić, M.; Hoche, J.; Cerezo, J.; Mitrić, R.; Santoro, F.; Bonačić-Koutecký, V. Predicting Fluorescence Quantum Yields for Molecules in Solution: A Critical Assessment of the Harmonic Approximation and the Choice of the Lineshape Function. *J. Chem. Phys.* **2020**, *152* (5), 054107. https://doi.org/10.1063/1.5143212.
7) Avila Ferrer, F. J.; Santoro, F. Comparison of Vertical and Adiabatic Harmonic Approaches for the Calculation of the Vibrational Structure of Electronic Spectra. *Phys. Chem. Chem. Phys.* **2012**, *14* (39), 13549. https://doi.org/10.1039/c2cp41169e.
8) Wang, L.; Ou, Q.; Peng, Q.; Shuai, Z. Theoretical Characterizations of TADF Materials: Roles of $\Delta G$ and the Singlet–Triplet Excited States Interconversion. *J. Phys. Chem. A* **2021**, *125* (7), 1468.
